# Africa-specific human genetic variation near *CHD1L* associates with HIV-1 load

**DOI:** 10.1038/s41586-023-06370-4

**Published:** 2023-08-02

**Authors:** Paul J. McLaren, Immacolata Porreca, Gennaro Iaconis, Hoi Ping Mok, Subhankar Mukhopadhyay, Emre Karakoc, Sara Cristinelli, Cristina Pomilla, István Bartha, Christian W. Thorball, Riley H. Tough, Paolo Angelino, Cher S. Kiar, Tommy Carstensen, Segun Fatumo, Tarryn Porter, Isobel Jarvis, William C. Skarnes, Andrew Bassett, Marianne K. DeGorter, Mohana Prasad Sathya Moorthy, Jeffrey F. Tuff, Eun-Young Kim, Miriam Walter, Lacy M. Simons, Arman Bashirova, Susan Buchbinder, Mary Carrington, Andrea Cossarizza, Andrea De Luca, James J. Goedert, David B. Goldstein, David W. Haas, Joshua T. Herbeck, Eric O. Johnson, Pontiano Kaleebu, William Kilembe, Gregory D. Kirk, Neeltje A. Kootstra, Alex H. Kral, Olivier Lambotte, Ma Luo, Simon Mallal, Javier Martinez-Picado, Laurence Meyer, José M. Miro, Pravi Moodley, Ayesha A. Motala, James I. Mullins, Kireem Nam, Niels Obel, Fraser Pirie, Francis A. Plummer, Guido Poli, Matthew A. Price, Andri Rauch, Ioannis Theodorou, Alexandra Trkola, Bruce D. Walker, Cheryl A. Winkler, Jean-François Zagury, Stephen B. Montgomery, Angela Ciuffi, Judd F. Hultquist, Steven M. Wolinsky, Gordon Dougan, Andrew M. L. Lever, Deepti Gurdasani, Harriet Groom, Manjinder S. Sandhu, Jacques Fellay

**Affiliations:** 1 Sexually Transmitted and Blood-Borne Infections Division at JC Wilt Infectious Diseases Research Centre, National Microbiology Laboratory Branch, Public Health Agency of Canada, Winnipeg, Manitoba, Canada; 2 Department of Medical Microbiology and Infectious Diseases, University of Manitoba, Winnipeg, Manitoba, Canada; 3 Wellcome Trust Sanger Institute, Hinxton, UK; 4 Department of Medicine, University of Cambridge, Cambridge, UK; 5 Peter Gorer Department of Immunobiology, School of Immunology and Microbial Sciences, King’s College London, London, UK; 6 Institute of Microbiology, Lausanne University Hospital and University of Lausanne, Lausanne, Switzerland; 7 Global Health Institute, School of Life Sciences, École Polytechnique Fédérale de Lausanne, Lausanne, Switzerland; 8 Swiss Institute of Bioinformatics, Lausanne, Switzerland; 9 Precision Medicine Unit, Biomedical Data Science Center, Lausanne University Hospital (CHUV) and University of Lausanne, Lausanne, Switzerland; 10 The African Computational Genomics (TACG) Research Group, MRC/UVRI and LSHTM Uganda Research Unit, Entebbe, Uganda; 11 Department of Non-Communicable Disease Epidemiology, Faculty of Epidemiology and Population Health, London School of Hygiene and Tropical Medicine, London, UK; 12 The Jackson Laboratory for Genomic Medicine, Farmington, CT, USA; 13 Department of Pathology, Stanford University School of Medicine, Stanford, CA, USA; 14 Department of Genetics, Stanford University School of Medicine, Stanford, CA, USA; 15 Division of Infectious Diseases, Feinberg School of Medicine, Northwestern University, Chicago, IL, USA; 16 Basic Science Program, Frederick National Laboratory for Cancer Research, National Cancer Institute, Frederick, MD, USA; 17 Laboratory of Integrative Cancer Immunology, Center for Cancer Research, National Cancer Institute, Bethesda, MD, USA; 18 Bridge HIV, San Francisco Department of Public Health, San Francisco, CA, USA; 19 Ragon Institute of MGH, MIT and Harvard, Boston, MA, USA; 20 Department of Medical and Surgical Sciences for Children and Adults, University of Modena and Reggio Emilia, Modena, Italy; 21 University Division of Infectious Diseases, Siena University Hospital, Siena, Italy; 22 Department of Medical Biotechnologies, University of Siena, Siena, Italy; 23 Epidemiology and Biostatistics Program, Division of Cancer Epidemiology and Genetics, National Cancer Institute, National Institutes of Health, Bethesda, MD, USA; 24 Institute for Genomic Medicine, Columbia University, New York, NY, USA; 25 Department of Medicine, Vanderbilt University School of Medicine, Nashville, TN, USA; 26 Department of Global Health, University of Washington, Seattle, WA, USA; 27 GenOmics and Translational Research Center and Fellow Program, RTI International, Research Triangle Park, NC, USA; 28 Medical Research Council/ Uganda Virus Research Institute & London School of Hygiene and Tropical Medicine, Uganda Research Unit, Entebbe, Uganda; 29 London School of Hygiene and Tropical Medicine, London, UK; 30 Center for Family Health Research—Zambia, Lusaka, Zambia; 31 Department of Epidemiology, Johns Hopkins University, Baltimore, MD, USA; 32 Department of Experimental Immunology, Amsterdam UMC, University of Amsterdam, Amsterdam, The Netherlands; 33 Community Health Research Division, RTI International, Berkeley, CA, USA; 34 Université Paris Saclay, Inserm UMR1184, CEA, Le Kremlin-Bicêtre, France; 35 APHP, Department of Clinical Immunology, Bicêtre Hospital, Le Kremlin-Bicêtre, France; 36 Vaccine and Therapeutics Laboratory, Medical and Scientific Affairs, National Microbiology Laboratory Branch, Public Health Agency of Canada, Winnipeg, Manitoba, Canada; 37 Institute for Immunology & Infectious Diseases, Murdoch University, Perth, Western Australia, Australia; 38 University of Vic—Central University of Catalonia, Vic, Spain; 39 IrsiCaixa AIDS Research Institute, Badalona, Spain; 40 Catalan Institution for Research and Advanced Studies, Barcelona, Spain; 41 CIBERINFEC, Instituto de Salud Carlos III, Madrid, Spain; 42 INSERM U1018, Université Paris-Saclay, Le Kremlin Bicêtre, France; 43 AP-HP, Hôpital de Bicêtre, Département d’Épidémiologie, Le Kremlin Bicêtre, France; 44 Infectious Diseases Service, Hospital Clinic—Institut d’Investigacions Biomèdiques August Pi I Sunyer (IDIBAPS), University of Barcelona, Barcelona, Spain; 45 National Health Laboratory Service, South Africa and University of KwaZulu-Natal, Durban, South Africa; 46 Department of Diabetes and Endocrinology, School of Clinical Medicine, University of KwaZulu-Natal, Durban, South Africa; 47 Department of Microbiology, University of Washington, Seattle, WA, USA; 48 Department of Infectious Diseases, Copenhagen University Hospital, Rigshospitalet, Copenhagen, Denmark; 49 Division of Immunology, Transplantation and Infectious Diseases, San Raffaele Scientific Institute, Milan, Italy; 50 School of Medicine, Vita-Salute San Raffaele University, Milan, Italy; 51 International AIDS Vaccine Initiative, New York, NY, USA; 52 Department of Epidemiology and Biostatistics, University of California, San Francisco, CA, USA; 53 Department of Infectious Diseases, Inselspital, Bern University Hospital, University of Bern, Bern, Switzerland; 54 Laboratoire d’Immunologie, Hôpital Robert Debré Paris, Paris, France; 55 Institute of Medical Virology, University of Zurich, Zurich, Switzerland; 56 Howard Hughes Medical Institute, Chevy Chase, MD, USA; 57 Basic Research Laboratory, Molecular Genetic Epidemiology Section, Frederick National Laboratory for Cancer Research and Cancer Innovative Laboratory, Center for Cancer Research, National Cancer Institute, Frederick, MD, USA; 58 Laboratoire Génomique, Bioinformatique et Chimie Moléculaire, EA7528, Conservatoire National des Arts et Métiers, HESAM Université, Paris, France; 59 Department of Medicine, National University of Singapore, Singapore, Singapore; 60 Queen Mary University of London, London, UK; 61 Kirby Institute, University of New South Wales, Sydney, New South Wales, Australia; 62 Department of Epidemiology & Biostatistics, School of Public Health, Imperial College London, London, UK; 63 MRC Centre for Environment and Health, School of Public Health, Imperial College London, London, UK; 64 Omnigen Biodata, Cambridge, UK

## Abstract

HIV-1 remains a global health crisis^1^, highlighting the need to identify new targets for therapies. Here, given the disproportionate HIV-1 burden and marked human genome diversity in Africa^2^, we assessed the genetic determinants of control of set-point viral load in 3,879 people of African ancestries living with HIV-1 participating in the international collaboration for the genomics of HIV^3^. We identify a previously undescribed association signal on chromosome 1 where the peak variant associates with an approximately 0.3 log_10_-transformed copies per ml lower set-point viral load per minor allele copy and is specific to populations of African descent. The top associated variant is intergenic and lies between a long intergenic non-coding RNA (*LINC00624*) and the coding gene *CHD1L*, which encodes a helicase that is involved in DNA repair^4^. Infection assays in iPS cell-derived macrophages and other immortalized cell lines showed increased HIV-1 replication in *CHD1L-*knockdown and *CHD1L-* knockout cells. We provide evidence from population genetic studies that Africa-specific genetic variation near *CHD1L* associates with HIV replication in vivo. Although experimental studies suggest that CHD1L is able to limit HIV infection in some cell types in vitro, further investigation is required to understand the mechanisms underlying our observations, including any potential indirect effects of CHD1L on HIV spread in vivo that our cell-based assays cannot recapitulate.

Despite advances in treatment and improved access to therapy, HIV-1 continues to be a global health problem, affecting an estimated 37.7 million people worldwide^1^. Although the annual incidence of HIV-1 has been declining since the advent of widespread antiretroviral therapy, this decline has slowed substantially since 2005, with notable increases in the number of newly infected adults in some regions^5^. Owing to the difficulties in developing an HIV-1 vaccine, eradicating established infection and avoiding drug resistance, combinations of current and new drug classes will be required for effective viral control, prevention and potential cure. Thus, a critical need remains to investigate new strategies to fight HIV-1.

In people who are not receiving antiretroviral therapy, set-point viral load (spVL)—defined as the mean log_10_[HIV-1 RNA copies per ml of plasma] during the chronic phase of infection—is an established correlate of disease progression^6,7^ and transmission potential^8,9^. HIV-1 spVL varies widely in the infected population and several intrinsic and extrinsic factors influence this variation, including host genetics^10^. Genome-wide association studies (GWAS) have consistently demon-strated that the major host genetic determinants of HIV-1 spVL in populations of European ancestries are the class-I human leukocyte antigen (HLA) and C-C motif chemokine receptor 5 (*CCR5*) genes, explaining around 15% of spVL variability^11–13^. Small GWAS analyses in individuals of African ancestries have recapitulated the HLA region as a key spVL determinant^14–16^, but have been relatively underpowered to detect more modest genetic effects. Owing to the disproportionate effect of HIV-1 and the high level of human genomic diversity within Africa^17^, large genome-wide studies of spVL control in populations of African descent are critical to enhance discovery and address existing health inequities.

To assess the host genetic contribution to HIV-1 spVL control in individuals of African ancestries, we performed a GWAS analysis in the component of the International Collaboration for the Genomics of HIV^3^ with individuals with African ancestries (*n* = 2,682), that is, those who present high genetic similarity to the African subset of the 1000 Genomes Project^18^ (Supplementary Fig. 1). This sample includes 7 genotype groups contributed by 11 cohorts/collaborating centres (Supplementary Table 1) with the majority (*n* = 2,535) being African American (groups 1–6) and a minority from Kenya (group 7, *n* = 147). After quality control and genome-wide imputation using a reference panel enriched with African haplotypes^17^, around 10 million common variants (minor allele frequency > 1%) were tested for association with spVL (Supplementary Fig. 2) per group, using linear regression models including sex and principal components to control for population stratification as covariates (Supplementary Fig. 3). After meta-analysis^19^, we observed two genomic regions with associated variants exceeding our screening significance threshold (*P* < 5 × 10^−8^; Extended Data Fig. 1a). The most strongly associated variant, rs1131446-T (*P* = 1.1 × 10^−39^, *β* = −0.683, s.e. = 0.052), is located on chromosome 6 and confers a synonymous change at amino acid 291 in HLA-B. Assessment of linkage disequilibrium between rs1131446 and sequence-based HLA types in a subset of our sample (*n* = 789) demonstrated that the T allele is linked to the presence of a valine residue at position 97 (Val97) within the HLA-B peptide-binding groove (*R*^2^ = 0.81; Supplementary Table 2). Val97 is exclusively carried by B*57 haplotypes, therefore, the association at rs1131446 is consistent with the known spVL decreasing effect of HLA-B*57:03 in African populations^15,20–22^.

We also observed a locus of association on chromosome 1, with the top variant, rs73001655-A, associating with a reduction in HIV-1 spVL of −0.298 (s.e. = 0.054) log_10_-transformed RNA copies per ml of plasma per allelic copy (*P* = 3.2 × 10^−8^). This variant falls upstream of a long intergenic non-coding RNA (*LINC00624*) and the region of association includes four protein coding genes (*PRKAB2, FMO5, CHD1L* and *BCL9*; Extended Data Fig. 1b). Analysis of the population distribution of rs73001655-A in the 1000 Genomes project^18^ showed that the A allele is observed only in individuals of African ancestries (Supplementary Table 3), with population frequencies ranging between 0.036 and 0.126 depending on the geographical region (Supplementary Table 4). The same genomic region was not associated with spVL in our previous analysis of 6,315 individuals of European ancestries^13^ (Supplementary Fig. 4), suggesting that the association signal is specific to populations of African descent.

To extend the statistical evidence for this association signal, we generated genome-wide genotype data from four additional groups of people living with HIV from three studies (Supplementary Fig. 1 and Supplementary Table 1). These included individuals living in east and southern Africa and individuals from Africa residing in Switzerland (*n*_replication_ = 1,197). The combined meta-analysis (*n*_combined_ = 3,879) sub-stantiated the locus of HIV-1 control on chromosome 1 (Fig. 1a and Extended Data Fig. 2), with 16 correlated variants exceeding the threshold for genome-wide significance in African populations^2^ (*P* < 5 × 10^−9^; Extended Data Table 1). The most significant variant in the combined dataset, rs59784663-G (*P*_combined_ = 6.4 × 10^−10^, *β*_combined_ = −0.304, s.e. = 0.049), is in strong linkage disequilibrium with rs73001655-A, the top variant in the discovery set (*R*^2^ = 0.839), and is situated between *LINC00624* and *CHD1L* (Fig. 1b). Consistent with the results from the discovery analysis, rs59784663-G is observed only in individuals of African ancestries with a frequency range of 0.04–0.12 across sub-populations (Supplementary Table 5). Indeed, all 16 variants exceeding genome-wide significance are exclusively observed in populations with African ancestries (Supplementary Table 6), underscoring the Africa-specific nature of this locus. The rs59784663-G allele conferred a reduction in spVL of 0.17–0.57 log_10_-transformed RNA copies per ml across all groups (mean = 0.30; Fig. 2a) with an approximately additive effect (Fig. 2b). Importantly, we did not observe any evidence for heterogeneity at rs59784663-G (Cochran’s *Q* = 4.87, *P* = 0.90) between groups in this study, suggesting a broad mechanism of action across viral subtypes and African populations. Notably, this reduction in spVL is of the same magnitude as that conferred by the CCR5Δ32 allele. CCR5Δ32 reduces the viral load and slows disease progression in heterozygote individuals, confers resistance to infection in homozygote individuals^23^ and is almost exclusively observed in populations of European ancestries^24^.

To assess whether the association was confounded by population structure, we performed sensitivity analyses using linear mixed models with genetic relatedness as a random effect, as well as linear regression accounting for inferred local ancestry in the African American subsample. These results were consistent with the meta-analysis (Supplementary Table 7 and Supplementary Fig. 5), demonstrating that the signal is not confounded by admixture, differential ancestry or cryptic relatedness.

To assess whether rs59784663-G may have reported effects beyond HIV-1, we performed a phenome-wide association study using the National Human Genome Research Institute GWAS catalogue^25^ and a GWAS analysis of 13 haematological traits in more than 2,600 individuals from East Africa^26^. The rs59784663-G variant is not associated with any other trait or disease in the NHGRI catalogue and was not associated with any of the haematological traits. Furthermore, none of the strongest signals observed in the haematological trait analysis was associated with HIV-1 spVL (Supplementary Table 8). Taken together, these data suggest that the association at rs59784663-G is specific to HIV-1 control.

We next examined the impact of variants in the region on gene expression. Owing to the Africa-specific nature of this signal, and limited representation of individuals with African ancestries in expression quantitative trait locus (eQTL) resources, we used RNA-sequencing (RNA-seq) data generated in lymphoblastoid cell lines from 600 individuals representing six African populations from the 1000 Genomes Study (Supplementary Table 9). In this sample, we did not detect any significant eQTLs for the transcripts underlying the association peak (*CHD1L* and *LINC00624*) nor did we find any evidence for colocalization between the spVL GWAS and eQTL signals (Supplementary Fig. 6). This result is consistent with other transcriptomic resources, as rs59784663-G is not a reported eQTL in GTEx^27^ or other studies including individuals of African ancestries^28–32^. Although this result precludes a large impact of rs59784663-G on basal gene expression, we note that the small sample size of individuals of African ancestries in these datasets does not rule out a more modest effect nor can we exclude an impact on gene expression in cell types and cell states relevant to HIV-1 infection.

To further examine the functional relevance of single-nucleotide polymorphisms (SNPs) at this locu*s*, we used PAINTOR^33^ to identify a set of variants that are likely to be causal. We identified 17 variants between *CHD1L* and *LINC00624* making up the 95% credible set (Supplementary Table 10). The most likely annotations assigned to this region were transcriptional activation in naive CD4^+^ T cells and transcription factor binding of *ZNF274* (Supplementary Fig. 7). We also performed a gene-level analysis using MAGMA^34^ to assess the enrichment of associated variants across the closest genes (*FMO5, PRKAB2, BCL9* and *CHD1L*). Of these, *CHD1L* showed the strongest aggregate signal of association (*P* = 6.2 × 10^−9^) with weaker statistical support for *FMO5* (*P* = 4.4 × 10^−4^), *PRKAB2* (*P* = 2.1 × 10^−3^) and *BCL9* (*P* = 0.06). CHD1L is a helicase involved in DNA repair through mediating chromatin relaxation after DNA damage^35^. CHD1L interacts with PARP1, an enzyme that is implicated in HIV-1 integration^36^ and Tat activation^37^. Taken together, these results suggest that *CHD1L* is the most likely causal gene in the region.

Given its proximity to the association peak and plausible functional connection to viral replication, we sought to assess the biological relevance of *CHD1L* in HIV-1 infection. We first assessed the dynamics of viral replication in U2OS cells, which have previously been used to investigate the stages of HIV-1 replication^38^ and host–viral protein interactions^39^. Wild-type (WT) and knockout (KO) U2OS cells were infected with the single-round pseudotyped HIV-1-based vector, NL4-3-deltaEnv-GFP/VSV-G. After 48 h, we observed a twofold increase in the percentage of GFP-positive cells in the *CHD1L*-KO cells compared with in the WT cells (Fig. 3a), suggesting that CHD1L may limit HIV replication in this model. We next assessed whether exogenous expression of CHD1L in KO cells could rescue the phenotype of reduced GFP expression to the levels observed in WT cells. *CHD1L-*KO U2OS cells were transfected with increasing amounts of *CHD1L* expression plasmid and infected with HIV-1 vector as above. We observed a stepped decrease in HIV-1-encoded GFP after increasing exogenous expression of CHD1L, supporting CHD1L as an inhibitor of HIV-1 infection (Fig. 3b and Supplementary Fig. 8).

To address the role of CHD1L in HIV infection in more physiologically relevant models, we screened several myeloid and T cell lines for CHD1L expression. We found that CHD1L is expressed in T cell lines at a similar level to that observed in U2OS cells, with lower expression in myeloid cell lines (Supplementary Fig. 9). Correspondingly, we performed infection assays in WT (CHD1L expressing) and KO Jurkat T cells and in monocytic THP-1 cells exogenously expressing CHD1L. In the Jurkat T cells, we did not observe any difference in HIV infection or p24 production in WT cells compared with in KO cells (Extended Data Fig. 3). However, consistent with results from U2OS cells, we observed lower levels of infection and a significant reduction in p24 production in monocytic THP-1 cells overexpressing *CHD1L* (Extended Data Fig. 4). These results suggest that CHD1L may function to reduce HIV infection and replication in a cell-type-specific manner.

The results from THP-1 cells suggest that the impact of CHD1L on HIV-1 may mainly occur in monocytic cells. Macrophages are a well-characterized HIV-1 reservoir^40^ and have a role in maintaining viraemia, particularly as CD4^+^ T cells decline^41,42^. To further investigate this, we performed KO and infection studies in macrophages derived from human induced pluripotent stem cells (iPS cells). One benefit of this model is that the parental iPS cells are amenable to genetic manipulation and do not induce a strong interferon response to transgenes, in contrast to other models. Moreover, these cells may follow the same developmental lineage of tissue-resident macrophages^43^, making them relevant to HIV infection^44^. To understand HIV-1 replication in this system, we generated one heterozygous (termed A12) and two homozygous (C11 and C12) *CHD1L*-KO clones using a CRISPR-based strategy (Fig. 4a,b and Supplementary Figs. 10 and 11) and differentiated the *CHD1L*^*+/+*^, *CHD1L*^*−/+*^ and *CHD1L*^*−/−*^ iPS cell clones into macrophages^45^ (Supplementary Fig. 12). Cells were then infected with HIV NL4-3-deltaEnv-GFP/ VSV-G (Extended Data Fig. 5a) and the GFP expression was assessed at 2 and 3 days after infection (Extended Data Fig. 5b,c). We observed a higher proportion of GFP-positive cells in the *CHD1L*^*−/−*^ clones, C11 and C12, compared with in the *CHD1L*^*+/+*^ cells, with the *CHD1L*^*−/+*^ clone A12 demonstrating an intermediate phenotype (Fig. 4c,d and Extended Data Fig. 5d,e). *CHD1L*-depleted cells also produced higher intracellular levels of p24, its full-length precursor Gag and the intermediate products compared with *CHD1L*^*+/+*^ cells (Fig. 4e,f). This was confirmed by the observation of increased viral Gag release—a by-product of single-round infection and a correlate of infection rate—from *CHD1L*-depleted cells (Fig. 4g and Extended Data Fig. 6). We next assessed the ability of KO of *CHD1L* to affect replication-competent virus. *CHD1L*^*+/+*^ or *CHD1L*^*−/−*^ (C12) iPS cell-derived macrophages were infected with HIV.BE_GIN (HIV-1 NL4-3 virus carrying a BaL envelope and a GFP-IRES-Nef cassette). A significant increase in p24 was observed in the supernatants from *CHD1L*^*−/−*^ cells compared with in the supernatants from *CHD1L*^*+/+*^ cells (Fig. 4h and Extended Data Fig. 7). These results demonstrate that CHD1L-expressing macrophages have a reduced ability to support HIV-1 replication compared with cells that are deficient in CHD1L expression.

Finally, we assessed the impact of CHD1L on HIV-1 replication in primary cells. CD14^+^ monocytes were isolated from donor blood and electroporated with Cas9 ribonuclear particles specific for *CHD1L* using 5 unique guides (gRNAs) alone and in combination and a guide targeting *CYPA* as a positive control. We observed substantial knockdown of *CHD1L* with three of the gRNAs and the multiplexed pool (Extended Data Fig. 8a). Polyclonal KO pools were then differentiated into monocyte-derived macrophages over 7 days. Macrophages were pretreated with SIV Vpx (to overcome SAMHD1 restriction and facilitate HIV-1 infection) and infected with HIV-1 NL4-3 dEnv Nef:IRES:GFP. The levels of p24 in the supernatant were measured 2 and 4 days after infection to monitor viral release, and GFP production was measured at day 4 to monitor the proportion of infected cells. At day 2, we observed a decrease in p24 production in both *CYPA-*KO and *CHD1L-*KO cells compared with in the non-targeting control cells, with *CHD1L-*KO cells producing comparable levels to non-targeting control cells at day 4 (Extended Data Fig. 8c,d). At day 4, GFP measurements showed an increased proportion of infected *CHD1L-*KO cells relative to non-targeting control cells for two out of the three gRNAs and the multiplex pool (Extended Data Fig. 8b). However, despite being largely consistent with the results from U2OS, THP-1 and iPS cells, this difference was not significant, possibly due to variability in KO and/or infection efficiency in the technical replicates and a low number of successfully collected cells.

African populations are still substantially under-represented in human genomic studies^46^ and experience the highest burden of HIV-1 infection. Here we provide strong genetic evidence for a region of association linked to host control of HIV-1 replication that is only variable in populations of African ancestries. This result underscores the importance of performing genomic studies in diverse ancestral populations to better address their specific medical needs and global health inequities.

*CHD1L*, the most likely causal gene at the locus, is involved in DNA repair and interacts with *PARP1*—a known HIV-1 host dependency factor that is involved in viral integration and transcription^36,37^. KO of *CHD1L* expression in in vitro HIV-1 infection models enhanced viral replication, and rescue experiments showed a decrease in viral infection with exogenous *CHD1L* expression. These results support the hypothesis that *CHD1L* acts to limit HIV-1 replication after entry, although the precise mechanism of action remains to be defined.

Notably, we observed the inhibitory effect of *CHD1L* only in some cell types—U2OS cells, macrophage-like THP-1 cells and iPS cell-derived macrophages. Monocyte and macrophage lineage cells are the initial target in mucosal HIV-1 infection and may influence viral amplification at a very early stage of infection. Furthermore, macrophages have been shown to sustain HIV-1 replication in the absence of T cells^47^ and tissue-resident macrophages contribute to the long-term maintenance of the viral reservoir (reviewed previously^48^). Although we did not observe a similar effect in T cell models, we did see it in U2OS cells, suggesting that CHD1L restriction may not be limited to macrophages. This cell specificity may be due to experimental differences; however, we cannot exclude a biological basis for these observations. There may also be additional, or alternative indirect effects of CHD1L on viral spread in vivo that cannot be discerned from these experiments.

As recently demonstrated through the response to the COVID-19 pandemic, large genomic studies of diverse populations can expand our knowledge of how host genetic variability impacts response to infection^49^. With around 1.5 million new HIV infections annually, the majority in sub-Saharan Africa, it is imperative to expand our understanding of how to restrict viral replication. Fully characterizing how *CHD1L* mediates HIV-1 control could lead to the development of therapeutics that improve treatment options for infected individuals.

## Online content

Any methods, additional references, Nature Portfolio reporting summaries, source data, extended data, supplementary information, acknowledgements, peer review information; details of author contributions and competing interests; and statements of data and code availability are available at https://doi.org/10.1038/s41586-023-06370-4.

## Methods

### GWAS

#### Samples, clinical values and ethics

Genomic data for the discovery set of 2,682 individuals were collected as part of seven independent genome-wide association studies combined under the International Collaboration for the Genomics of HIV (ICGH) as previously described^3^ (Supplementary Table 1 and Supplementary Note 2). Genome-wide genotype data for the replication set were collected from three additional, independent cohorts. The Rural Clinical Cohort (RCC) is a subset of the General Population Cohort (GPC), a population-based open cohort study based in Kyamulibwa sub-county, Uganda, established in 1989 by the Medical Research Council, UK in collaboration with the Uganda Virus Research Institute to examine trends in the prevalence and incidence of HIV infection and their determinants, previously described elsewhere^50^. The RCC included a combination of incident and prevalent cases of HIV infection, identified through screening within the GPC, and has been characterized previously^50^. The International AIDS Vaccine Initiative is a non-profit organization dedicated to accelerating the development of vaccines to prevent AIDS. Participants with HIV infection were genotyped using the Illumina 1M Duo genotyping array in two groups. The Swiss HIV Cohort Study (SHCS) is an ongoing multicentre research project dealing with adults infected with HIV aged 16 years or older residing in Switzerland. Participants with African ancestries were genotyped using the specialized Human Heredity and Health in Africa (H3Africa) Illumina genotyping array designed by H3ABioNet (https://h3abionet.org/h3africa-chip). In two studies, the International HIV Controllers Study/AIDS Clinical Trials Group and the International AIDS Vaccine Initiative, genotyping was performed in multiple batches (Supplementary Table 1). In these instances, the batches were analysed independently and combined through meta-analysis.

All of the patients were infected with HIV-1 as confirmed by the initial study. spVL was defined as the log_10_-transformed average number of HIV-1 RNA copies per ml of plasma from at least two viral load measurements during the chronic phase of infection and before the initiation of antiretroviral therapy. All of the participants gave written informed consent for genetic testing and ethical approval was obtained from institutional review boards for each of the respective contributing centres (Supplementary Note 2).

#### Genotyping and quality control

Genome-wide genotyping was performed by each centre using various platforms (Supplementary Table 1). Per group, variants were removed that displayed high missingness (>2%), low minor allele frequency (<1%) or deviation from Hardy–Weinberg equilibrium (*P* < 5 × 10^−6^). Samples were excluded if they exhibited high missingness (>2%), high heterozygosity (Inbreeding coefficient >0.1 or <−0.1) or shared greater than 12.5% similarity in an identity by descent analysis. In the absence of strand information for several genotype arrays, strand-ambiguous SNPs were excluded across all cohorts for consistency. Where necessary, genotype mapping files were lifted over using the UCSC Liftover tool onto GRCh37, and any unmapped regions were excluded. Variant and sample quality control was performed using plink (v.1.9)^51^. We inferred participant ancestries using principal components analysis comparing patient genotype data to the 1000 Genomes phase 3 reference sample^18^. For the present analysis, only samples clustering with the 1000 Genomes African populations were included (Supplementary Fig. 1). Principal components were calculated using EIGENSTRAT within EIGENSOFT (v.7.2.1)^52^.

#### Phasing and imputation

Imputation of ungenotyped variants was performed using a reference panel constructed of data from the African genome variation project^17^ merged with the 1000 Genomes project phase 3 sample. Evaluation of this panel has demonstrated improved performance in African populations compared to the 1000 Genomes data alone. Details regarding the generation of panel, and SNP content have been previously published^17^. For the RCC cohort alone, given that this cohort was a subset of the much larger GPC cohort (*n* = 4,778) including HIV uninfected individuals, pre-phasing with SHAPEIT2 (v.2.12)^53^ was performed to maximize phasing accuracy, after which imputation was performed using IMPUTE2 (v.2.3.2)^54^. For the other cohorts, phasing and imputation were performed together using the IMPUTE2 pipeline.

#### Association testing and meta-analysis

Per genotype group, association was tested between variant dosage and spVL using linear regression models including principal components to control for population structure. Principal components were calculated per group using EIGENSTRAT on a set of high-quality variants (minor allele frequency > 0.05, missing < 0.02, hwe *P* > 0.000005) pruned for linkage disequilibrium (*R*^2^ < 0.5). Per group, principal components were tested for association with spVL and were included in the genetic analysis if they associated with the phenotype at *P* < 0.05. In all of the groups, this was sufficient to account for inflation in the test statistic (Supplementary Fig. 3). Evidence for association was combined across groups using inverse-variance-weighted meta-analysis in a fixed-effects framework. Only variants with imputation quality scores >0.6 in all cohorts were considered for analysis. Linear regression was performed using plink (v.1.9)^51^ and meta-analysis was performed using meta (v.1.7)^19^.

#### Sensitivity analysis of association tests

To ensure that the results were not confounded by admixture, ancestry or cryptic relatedness, the following sensitivity analyses were conducted. (1) We performed association testing using linear mixed models as implemented in GCTA (v.1.25.3), including a genetic relatedness matrix (GRM) as a random effect^55^. Given the limited overlap across genotype groups, we selected a common set of variants that were imputed with high confidence in all of the groups (imputation info score > 0.9) to construct the GRM. These variants were then filtered for frequency (>0.01), missingness (<0.02), Hardy–Weinberg equilibrium (*P* > 0.000001) and were pruned for linkage disequilibrium (*R*^2^ < 0.5). Moreover, we removed variants within the associated regions on chromosome 6 (29–34 Mb, corresponding to the MHC region) and chromosome 1 (146.5–147.1 Mb) to avoid proximal contamination. Association was then tested genome-wide using the mixed-linear-model-based association analysis (mlma) with all groups combined, including the GRM as a random effect. (2) For all African American individuals, local ancestry was calculated across chromosome 1 using Efficient Local Ancestry Inference (ELAI v.1.0)^56^ using the 1000 Genomes CEU and YRI samples as reference populations. Per group, association was tested between variants at the chromosome 1 locus and spVL including the average predicted dosage of African haplotypes (that is, carrying 0, 1 or 2 African haplotypes) across the chromosome 1 region as a covariate. Individuals from African cohorts (groups 7–11; Supplementary Table 1) were assigned a dosage of 2. Association results were then meta-analysed across all groups using inverse-variance weighting. Association results for rs59784663 in both analyses were highly consistent with the linear regression plus meta-analysis results (Supplementary Table 7).

#### Assessment of correlation between variants

To address the relationship between the top associated variant in the MHC region and known causal alleles at HLA class I genes, we performed a linkage analysis between rs1131446-T and classical HLA types and protein sequences available in a subset of our African American sample^15^. In this sample, we have previously inferred local ancestry across the MHC and identified 789 individuals predicted to carry only African haplotypes. To eliminate confounding due to admixture, we performed *R*^2^ calculations using only these individuals. Correlation was calculated between dosage of rs1131446-T and classical class I HLA alleles (4-digit resolution) and variable amino acid positions within class I proteins using R (v.3.4.3).

Correlation between variants in the chromosome 1 region was calculated in the African subset of the 1000 Genomes Project Phase3 reference data. We extracted the 1 Mb region around rs59784663 (146.4–147.4 Mb, GRCh37) and calculated *R*^2^ and *D*′ using plink (v.1.9).

#### Phenome-wide association study

To assess the specificity of the results in the *CHD1L* region to HIV viral load, we examined the association between relevant SNPs and phenotypes in the NHGRI catalogue and multiple haematological traits across the GPC from Uganda^57^, including 2,744 individuals participating in a large genomic study investigating the heritability of cardiometabolic traits^58^.

#### Identification of the 95% credible set

To determine the posterior probability of one or more genetic variants being causal for our phenotype, we used PAINTOR (v.3.1)^33^ incorporating linkage disequilibrium patterns, GWAS summary statistics, and functional variant annotations in an empirical Bayes framework. All of the variants in the associated region (chromosome 1: 146.47–147.2 Mb, build 37) in linkage disequilibrium (*R*^2^ > 0.2) with rs59784663 were included in this analysis. Functional annotations were included from the Epigenetics RoadMap^59^, a database of transcriptional enhancers^60^, DNase I hypersensitivity screens^61^ and the Functional Annotation of Mammalian Genomes 5 (FANTOM5) consortium^62^. Annotations were selected from epigenetic markers in CD4^+^ T cells, monocytes, peripheral blood mononuclear cells and transcription-factor-binding sites (*n* = 418) with 260 annotations mapping to at least 1 variant.

#### Gene-level analysis

We used Multi-marker Analysis of GenoMic Annotation (MAGMA v.1.10)^34^ to conduct a gene-level analysis. SNPs were assigned on the basis of the NCBI 37.3 human reference build with a 10 kb window. To account for diverse genetic architectures in the chromosome 1 region, we specified multiple gene analysis models (--gene-model multi=all), which aggregated *P* values generated from the principal component regression, mean SNP association and top SNP association models. We allowed the model to use adaptive permutation for determination of *P* values for each gene. We applied MAGMA using individual-level genotype data, spVL phenotypes and the first principal component as a covariate.

### Identifying eQTLs in lymphoblastoid cell lines from African populations

#### Populations included

We performed RNA-seq analysis of lymphoblastoid cell lines (EBV-transformed peripheral B lymphocytes) from 596 individuals from 6 diverse populations of Africa. These include 5 populations from the 1000 Genomes Project Phase 3 (ESN, *n* = 99; GWD, *n* = 112; LWK, *n* = 97; MSL, *n* = 83; YRI, *n* = 41) with publicly available genotype data and an additional population of Maasai individuals from the International HapMap Project (MKK, *n* = 164) not included in the 1000 Genomes Project for whom we performed full genome sequencing to determine the genotype. For the Maasai set, whole-genome sequencing was performed to high coverage (30×) at the Wellcome Trust Sanger Institute. Variants (SNPs and small indels) were called using the GATK’s HaplotypeCaller following best practices in an identical manner as was used in the 1000 Genomes Project. Variant qualities were generated by the VQSR method using the suggested 1000 Genomes variants and gold standard INDEL call sets. The tranche thresholds for the final set of the filtered variants were 99.5 for the SNPs and 99.0 for the indels as suggested by the GATK best practices guidelines.

#### RNA extraction and sequencing

RNA was extracted from lymphoblastoid cell lines obtained from the Coriell Cell Repository. The samples were selected from unrelated individuals with no total RNA-seq data available to date. Cell lines were processed at Coriell in batches in which each population was represented per-batch to reduce the batch effect. Cell cultures were expanded and 10^7^cells per line were pelleted, treated with RNAProtect (Qiagen) and stored at −80 °C until shipment. After a second randomization of samples, RNA was extracted by Hologic/ Tepnel Pharma Services using the RNeasy PLUS mini kit (Qiagen).

Library preparation for RNA-seq was performed using the standard automated Kapa stranded mRNA library preparation protocol, followed by RNA-seq analysis on the HiSeq 2500 at the Wellcome Trust Sanger Institute using paired-end sequencing with 75 bp reads and 12 samples per sequencing lane. Per lane, samples were randomized with respect to their population, Coriell batches and Hologic RNA extraction batches.

#### Read mapping, quantification and normalization

RNA-seq reads were inspected using the FastQC tool for quality control. Reads were trimmed using Cutadapt for poly(A) and adaptors before mapping. Reads were mapped to the human reference sequence v38 using the STAR alignment tool in two-pass mode. Quantification of gene expression was performed with htseq-count (v.0.9.1) using genes as features. The gene interval was defined as the union of all exons. Reads mapping to the gene were counted including partial alignments. Reads mapping to multiple genes were marked as ambiguous and were not included in the final counts. After quantification, there were 58,288 gene counts per sample for which approximately 85% of reads were mapped unambiguously. Normalization was performed using the DESeq2 tool^63^ in which counts were first scaled with respect to the library size for each sample followed by variance-stabilized normalization. Normalization was performed for each population separately.

#### eQTL analysis

eQTL mapping was performed for the 2 Mb region around *CHD1L*, including 33 genes with measurable expression. We restricted our search to *cis*-eQTLs by selecting variants within 50 kb of each gene’s start and end positions. Per population, *cis*-eQTLs were identified by linear regression whereby normalized gene expression was regressed on variant dosage correcting for covariates using Matrix eQTL^64^. Covariates included population principal components calculated from genotype data, metadata on known technical variables and unobserved confounding variables detected using surrogate variable analysis. For each gene, the significance of the most highly associated variant was determined from empirical *P* values from a *β* distribution fitted to an adaptive permutation (1,000 permutations). False-discovery rate values for each variant–gene pair were estimated using the Benjamini–Hochberg procedure. Data were combined across studies using a random-effects model that assumes a different underlying effect size for each population implemented by METASOFT. Variants with a meta-analysis *P <* 0.05 were considered to be significant eQTLs for each gene.

#### Colocalization of genome-wide association and eQTL results

We used eCAVIAR (v.2.1)^65^ to test for GWAS–eQTL colocalization and to calculate the colocalization posterior probability. We selected a locus of 740 kb around *CHD1L* and calculated *z*-scores using the *β* and s.e. from the summary statistics. Colocalization was calculated for each gene in the region using the GWAS and eQTL results for that gene.

### *CHD1L* KO, overexpression and HIV-1 infection in U2OS cells

#### Transfection rescue of *CHD1L*-KO U2OS cells

U2OS WT and *CHD1L*-KO cells were a gift from G. Timinszky^66^ and maintained in DMEM + l-glutamine + 10% FBS + penicillin–streptomycin. pLVX-EF1a-IRES-mCherry-CHD1L was made by cloning the coding sequence for full-length human *CHD1L* into the commercially available pLVX-EF1α-IRES-mCherry vector (TakaraBio) using the EcoRI–XbaI sites. Successful clones were verified by a diagnostic digestion and sequencing. Cells were plated at 1 × 10^5^ cells per well of a 24-well plate. The next day, cells were transfected as follows: 150 μl DMEM without FBS or FCS and penicillin–streptomycin was incubated with 2 μl TransIT-LT1 20 °C for 5 min. The indicated quantity of CHD1L expressor (pLVX EF1a IRES mCherry, *CHD1L*) made up to 100 ng with pBlueScript, was added to the transfection mix and incubated at 20 °C for 30 min. Cells were washed with PBS before the addition of the transfection mix. After 4 h, 1 ml of complete medium was added, and cells were maintained overnight at 37 °C before transduction with HIV vector and analysed as described below.

### *CHD1L* KO and overexpression in Jurkat T cells and THP-1 cells

#### Generation of *CHD1L*^*−/−*^ Jurkat cells

Mono and biallelic *CHD1L* KOs in Jurkat E6.1 (ATCC, TIB-152) were generated under contract by Applied StemCell using CRISPR–Cas9 technology (Supplementary Note 1). Exon 10 was targeted independently with two gRNAs (g2 CRISPR and g4 CRISPR in Supplementary Table 11). In brief, cells were co-electroporated with a proprietary Cas9-expressing plasmid and one of two gRNA expressing plasmids (Supplementary Table 11). Then, 2 days after electroporation, cells were selected with 4 μg ml^−1^ of puromycin for 48 h and then processed for single-cell cloning in a 96-well plate. Cells were propagated to grow for 15 to 20 days. Genomic DNA from single-cell colonies was isolated and the targeted locus was amplified using gene-specific primers (2F and 2R in Supplementary Table 11) and sequenced using a gene-specific primer (F1 in Supplementary Table 11). Positive clones were amplified and subsequently regenotyped.

#### *CHD1L* overexpression in THP-1

THP-1 were maintained in RPMI + 10% FBS + 50 μg ml^−1^ gentamycin (R10). Non-differentiated THP-1 cells were transduced with HIV-based lentiviral vectors carrying either pLVX-EF1α-IRES-mCherry (control) or pLVX-EF1α-IRES-mCherry-CHD1L (*CHD1L* overexpression) as genome of interest. In brief, 2 million cells were transduced in 10 wells of a U-bottomed 96-well plate (200,000 cells per well) with 100 ng of vector in 100 μl of R10 containing 4 μg ml^−1^ of polybrene (Merck, TR-1003) in each well. After spinoculation of 90 min at 1,500*g*, cells were pooled together in a 24-well plate at the concentration of 10^6^ cells per ml. mCherry expression was assessed by flow cytometry 48 h after transduction. mCherry-positive and mCherry-negative cells were sorted and further amplified for 2 weeks. mCherry sorted and unsorted cells were retained for further differentiation and HIV infection.

#### Differentiation and infection of THP-1 cells

A total of 1 million *CHD1L*-overxpressing THP-1 or control cells were differentiated into macrophages by incubating the cells in R10 supplemented with 25 nM PMA (Merk, P8139) in a well of a 24-well plate at 37 °C under 5% CO_2_ for 48 h as previously described^67^. After 48 h differentiation, the culture medium was replaced with fresh R10 and cells were left to recover for 24 h before being infected. Differentiated THP-1 cells were infected with 100 ng of p24 equivalent of HIV NL4.3-deltaEnv-GFP/VSV-G in 500 μl of R10 containing 4 μg ml^−1^ of polybrene and spinoculated for 90 min at 1,500*g*. After spinoculation, the cells were supplemented with 500 μl of R10 culture medium and incubated at 37 °C for 24 h before washing out the virus. Viral particle release was assessed by p24 ELISA (Innotest HIV Antigen monoclonal antibody, Fujirebio) of the supernatant collected at day 3 after infection.

### *CHD1L*-KO and transduction in human iPS cell-derived macrophages

#### Human iPS cell culture

The human iPS cell line KOLF2 was generated by the Human Induced Pluripotent Stem Cells Initiative consortium at the Sanger Institute using fibroblasts from a healthy adult male with the CytoTune 1 Sendai method^68^. Specifically, the subline KOLF2-C1 isolated by single-cell cloning was used for the generation of the *CHD1L-*KO clones. KOLF2-C1 cells were kept under feeder-free conditions in TeSR-E8 medium (StemCell Technologies), on tissue culture plates coated with Synthemax II-SC substrate (Corning). Cells were dissociated from the plates using gentle cell dissociation buffer (StemCell Technologies) and passaged every 3–5 days.

#### Generation of *CHD1L*^*−/−*^ human iPS cells

Monoallelic and biallelic KOs in KOLF2-C1 cells were generated using a synthetic cassette knock-in CRISPR-based strategy that was found to minimize the potential for off-target effects^69^. The synthetic cassette contains a puromycin-resistant gene that, when inserted into the targeted genome, allows the selection of the edited clones. *CHD1L* exon 10 was targeted using two gRNAs and the puromycin-resistance cassette with homologous arms (Supplementary Fig. 10a). Double-stranded breaks at the gRNA target sequences were generated through the introduction of the Cas9 endonuclease in combination with the two gRNAs designed against exon 10, and the puromycin cassette was inserted through the homology-directed repair pathway. For biallelic KO clones, the second allele was repaired using the non-homologous end-joining pathway to introduce small nucleotide insertions or deletions (indels) at the double-stranded-break site leading to the introduction of frameshift mutations and a premature stop-codon. gRNAs were designed using the online software WGE—CRISPR design tool (https://www.sanger.ac.uk/htgt/wge/)^70^.

The following vectors were used for the generation of the *CHD1L*^*−/*−^ iPS cells: (1) the donor DNA template plasmid containing the synthetic cassette; (2) plasmids carrying the gRNA sequences; and (3) the Cas9-expressing vector. In brief, for the generation of the donor DNA template plasmid, an intermediate-targeting vector for *CHD1L* was constructed using GIBSON assembly of four fragments (pUC19 vector, 5′ homology arm, R1-pheS/zeo-R2 cassette and 3′ homology arm). Homology arms upstream and downstream of exon 10 were amplified by PCR from KOLF2 human iPS cell genomic DNA using the primers listed in Supplementary Table 12. The pUC19 vector and the R1-pheS/zeo-R2 cassette cut with EcoRV, and the homology arm PCR were gel-purified (QIAquick, Qiagen). The GIBSON assembly reactions (Gibson Assembly Master Mix, NEB) were transformed into NEB 5-alpha competent cells and the resulting clones were analysed by Sanger sequencing using the primers listed in Supplementary Table 12. Subsequently, the intermediate-targeting vector was converted into a donor plasmid through a Gateway exchange reaction. The LR Clonase II Plus enzyme mix (Invitrogen) was used in a two-way reaction exchanging the R1-pheSzeo-R2 cassette with the pL1-EF1aPuro-L2 cassette, as described previously^71^. The latter was generated by cloning synthetic DNA fragments of the EF1a promoter and puromycin into one of the pL1/L2 vectors as described previously^71^. After the Gateway reaction and selection on YEG + carbenicillin (50 μg ml^−1^) agar plates, the correct donor plasmid was confirmed by Sanger sequencing. Plasmids carrying single gRNA sequences (Supplementary Table 12) were generated by cloning forward and reverse strand oligos (IDT) into the BsaI site of either U6_BsaI_gRNA or p1260_T7_BsaI_gRNA vectors (provided by S. Gerety). Positive clones were verified by Sanger sequencing. The Cas9-coding plasmid (hCas9) was purchased from Addgene (41815).

Human iPS cells were dissociated to single cells and nucleofected (Amaxa2b nucleofector, LONZA) with the Cas9-coding plasmid, sgRNA plasmids and donor plasmid. After nucleofection, cells were selected with 0.25 μg ml^−1^ puromycin for 11 days and single colonies were transferred into two duplicated 96-well plates. Once confluent, colonies from one plate were frozen and those from the second plate were lysed for genotyping. Insertion of the cassette into the correct locus was confirmed by PCR with gene-specific (GF and GR) and cassette-specific (ER and PF) primers (Supplementary Table 12 and Supplementary Fig. 10b). To check the CRISPR site on the non-targeted allele, PCR products were generated using primers PF1 and PR1 (Supplementary Table 12) and Sanger sequenced using primers SF1 and SR1 (Supplementary Table 12 and Supplementary Fig. 10c–e). Positive clones were amplified and subsequently regenotyped.

#### Macrophage differentiation iPS cells

Before initiation of the differentiation protocol, the WT and KO KOLF2-C1 cells were adapted to feeder-dependent conditions for two passages on a monolayer of mitotically inactivated mouse embryonic feeder (MEF) cells in Advanced DMEM/F12 (Gibco), supplemented with 20% KnockOut Serum Replacement (Gibco), 2 mM l-glutamine (Sigma-Aldrich), 0.055 mM β-mercaptoethanol (Sigma-Aldrich) and 4 ng ml^−1^ recombinant human fibroblast growth factor (rhFGF) basic (R&D). Human iPS cells were differentiated into macrophages using an established method^45^ involving three steps: (1) embryoid body (EB) formation; (2) generation of monocyte-like myeloid progenitors from the EBs; and (3) terminal differentiation of the progenitors into macrophages. For EB formation, iPS cells were dissociated from the feeder plates using a mix of collagenase (1 mg ml^−1^) and dispase (1 mg ml^−1^) (both Thermo Fisher Scientific) and transferred to low-adherence plates (Sterilin) in feeder-dependent iPS cell medium without rhFGF for 4 days. On day 4, EBs were transferred to gelatin-treated tissue culture plates in serum-free X-Vivo 15 medium (Lonza) supplemented with 2 mM l-glutamine (Sigma-Aldrich), 0.055 mM β-mercaptoethanol (Sigma-Aldrich), 50 ng ml^−1^ macrophage colony-stimulating factor (M-CSF, R&D) and 25 ng ml^−1^ interleukin-3 (IL-3, R&D), to allow the generation of the myeloid progenitor cells. For the terminal differentiation into mature macrophages, precursor cells were plated in RPMI 1640 (Gibco) supplemented with 10% FBS (Sigma-Aldrich), 2 mM l-glutamine and 100 ng ml^−1^ hM-CSF for 7 days.

#### Macrophage characterization by flow cytometry

Macrophages derived from WT and *CHD1L-*depleted iPS cells (iPSDM) were detached using Lidocaine solution (4 mg ml^−1^ Lidocaine, 5 mM EDTA in PBS, 15 min 37 °C) and subsequently introduced, into 96-well round-bottom plates at a density of 10^5^ cells per well, with 100 μl of FACS blocking buffer (5% FCS in PBS, 0.1% sodium azide) containing 2 μl of Trustain Fc block for 30 min at 4 °C. Conjugated antibodies against the three canonical macrophage antigens CD14, CD16 (FCGR3A/FCGR3B) and CD206 (MRC1) (mouse anti-human CD14 Alexa Fluor 488, CD16 APC-Cy7 and CD206 APC, respectively, Becton Dickinson) were added to each well and the cells were incubated for a further 30 min according to the manufacturer’s instructions. Cells were washed twice with FACS buffer, resuspended in PBS and analysed using the BD LSR Fortessa (BD Biosciences). Data were analysed using FlowJo (v.10.1). Both the homozygous and the heterozygous iPSDM clones expressed the macrophage markers CD14, CD16 and CD206 (Supplementary Fig. 12b).

#### VSV-G-pseudotyped HIV-based vector and replication-competent virus preparation

VSV-G-pseudotyped HIV-GFP used for macrophage transduction was produced by transient co-transfection of HEK293T cells (ATCC) with pNL4.3-deltaEnv-GFP (NIH AIDS Research and Reference Reagent program, 11100) and pVSV-G, using Trans-IT LT1 reagent (Mirus Bio). The medium was changed one day after transfection and the supernatant was collected 48 and 72 h after transfection. Supernatant containing virus-like particles was passed through a 0.45 μm filter to remove cell debris and stored at −70 °C until use. Relative viral titres were determined by p24 ELISA and titration on HEK293T cells. Replication-competent virus was produced as above except that cells were transfected with the single plasmid HIV-1NL4-3 BaL env GFP-IRES-Nef.

VSV-G-pseudotyped HIV-GFP used for Jurkat transduction was produced as described above except transfections were performed using jetPrime (Polyplus transfection) and the VSV-G-envelope-coding plasmid was pMD2G (Addgene, 12259). Viral particles were collected 48 h after transfection, filtered through 0.45 μm filters and concentrated on Centricon filters (Centricon Plus-70/100K, Millipore). Viral titres were measured by HIV-1 p24 ELISA (Innotest HIV Antigen monoclonal antibody, Fujirebio) according to the manufacturer’s recommendations.

#### Vector transduction and post-transduction flow cytometry

Equal numbers of WT, heterozygous and homozygous iPSDMs (1 × 10^5^) were seeded into 12-well plates. To infect cells, the culture medium was removed, and medium containing viral vector was applied to the cells. After 24 h, the medium was replaced with RPMI (Gibco) supplemented with 10% FBS (Sigma-Aldrich), 2 mM l-glutamine (Sigma-Aldrich) and 5% penicillin–streptomycin (Gibco). Infection levels were assessed by flow cytometry analysis of GFP expression. Then, 2 or 3 days after transduction, cells were washed once with PBS and stained with DRAQ-7 (Abcam) according to the manufacturer’s instructions. Cells were detached from the wells for flow cytometry with 5 mM EDTA in PBS, with scraping as necessary. Flow cytometry was conducted on the BD Accuri C6 flow cytometer (BD Biosciences). Data were analysed with FlowJo v.10. Dead cells were excluded by DRAQ-7 staining and debris by light scattering. To circumvent autofluorescence, GFP positivity was controlled through FL1/FL2 comparison (Extended Data Fig. 5b,c).

WT and *CHD1L-*KO Jurkat cells were transduced in the presence of 4 μg ml^−1^ polybrene. For dose–response experiments, 100,000 cells were incubated with increasing concentrations of virus, ranging from 0.1 to 300 ng p24 in 100 μl final volume, for 2 h at 37 °C. After infection, cells were washed once with 100 μl of RPMI supplemented with 10% FBS and collected 48 h after infection. For the kinetic experiments, 100,000 cells were incubated with 300 ng p24 virus for 2 h at 37 °C, washed once with 100 μl of RPMI supplemented with 10% FBS and collected at 12, 24 and 36 h after infection. The collected samples were washed in 100 μl Robosep buffer (STEMCELL technologies) and fixed in 200 μl of BD CellFix 1× (BD-Bioscience). HIV-encoded GFP expression was assessed by flow cytometry using a BDAccuri C6. Data were analysed using FlowJo v.10.

#### Viral particle release from infected iPSDMs

WT or *CHD1L*^−/−^ iPSDMs were infected with equivalent concentrations of HIV NL4.3-deltaEnv-GFP/VSV-G (inter-experimental values ranged between 80 and 150 ng ml^−1^) at 4 °C and moved to 37 °C to synchronize infection. Virus was removed by washing and the medium was replaced at 2–3 h after infection. The supernatants were collected at 6, 24, 36 and 48 h after infection and neutralized with NP40 (0.5%). Neutralized supernatants were diluted 1/1,000 and 100 μl was used for viral Gag particle release assessment by p24 ELISA (Innotest HIV Antigen monoclonal antibody, Fujirebio) according to the manufacturer’s instructions. Statistical analysis was performed using GraphPad Prism (v.9.4.0).

#### RNA extraction, sequencing and qpcr

Total RNA was extracted from three independent samples of WT and KO iPS cells using the RNeasy Mini Kit (Qiagen) according to the manufacturer’s protocol. Genomic DNA removal was performed using the on-column digestion with the RNase-Free DNase Set (Qiagen). RNA concentration and integrity were measured on the Agilent 2100 Bioanalyzer using RNA 6000 Nano total RNA kits.

RNA-seq libraries were constructed using poly(A) selection using the KAPA stranded mRNA-seq kit (KAPA Biosystems). KAPA libraries were quantified using the Quant-iT plate reader and pooled automatically using the Beckman Coulter NX8. The samples were sequenced on the Illumina HiSeq 2500 using V4 chemistry with 75 bp paired-end reads. Approximately 50 million reads were generated for each clone and inspected for quality control using the FastQC tool. All of the samples had the necessary read quality. The adaptors and the over-represented poly(A) sequences were trimmed from the reads using Cutadapt. Reads were mapped to the human genome reference sequence (GRCh38/ hg38) using STAR in two-pass mode. In the first alignment, the default Ensembl human gene annotations for hg38 v83 were used for the mapping. The novel junctions from the first pass and the default Ensembl junctions were merged before the second and final alignment to the reference sequence. The exon read counts were extracted from the mapping files using the feature Counts tool. These counts were transformed to fragments per kilobase per million (FPKM) values that were used for comparing the expression levels between the WT cells and mutant clones.

cDNA for qPCR analysis was synthesized using the QuantiTect Reverse Transcription Kit (Qiagen). TaqMan Fast Advanced Master Mix (Thermo Fisher Scientific) was used for qPCR with the following gene expression assays: Hs00610997_m1 (*CHD1L*, full-length transcript), Hs01019194_m1 (*CHD1L*, truncated transcript) and Hs99999907_m1 (*B2M*). Each reaction was conducted in triplicate on the StepOnePlus System (Thermo Fisher Scientific). The Δ*C*_t_ method was used for determining gene expression values relative to the expression of *B2M*.

#### Protein extraction and western blot

Total proteins were extracted from iPS cells, Jurkat KO clones, induced pluripotent stem-cell-derived macrophages and cell lines using RIPA buffer (Sigma-Aldrich) or cell culture lysis reagent (Promega) and normalized for total protein content where indicated using the Pierce BCA Protein Assay Kit (Thermo Fisher Scientific). Rabbit monoclonal antibodies against human CHD1L (Cell Signaling, 13460), mouse monoclonal antibodies against GAPDH (Thermo Fisher Scientific, MA5-15738) and rabbit monoclonal anti-HIV p24 antibodies (ARP 313, NIH AIDS Reagents Program) were used for western blot detection of the specific proteins. Densitometry analysis was performed using ImageJ.

#### ELISA assay for intracellular p24 determination

CA-p24 concentration was determined by ELISA in reference to known standards using anti-p24 capture antibodies (Aalto Bio Reagents) and alkalin-phosphatase-conjugated anti-p24 monoclonal antibodies (EH12-AP, Aalto Bio Reagents). In brief, half area 96-well plates were incubated with coating antibody overnight and then blocked in 5% bovine serum albumin in 1× Tris-buffered saline (TBS). The plates were washed four times in TBS and incubated with standard/sample for 90–360 min at 20 °C. The plates were washed again and incubated with alkaline-phosphatase-conjugated anti-HIV CA-p24 mouse monoclonal antibodies diluted 1:16,000 in 1× TBS, 2% milk, 20% sheep serum and 0.5% Tween-20. After a final wash, detection was performed using Lumiphos Plus reagent (Lumigen) on the Glomax luminometer (BMG Labtechnologies). p24 levels (ng ml^−1^) were normalized to total protein content as determined by bicinchoninic acid assay or Ponceau staining. Levels are expressed as the fold change compared with WT cells.

#### Statistical analyses of HIV-1 production

Statistical significance was assessed using GraphPad Prism software. Wilcoxon matched-pairs signed-rank tests were used for two-group comparisons.

### *CHD1L* KO and infection in primary human macrophages

#### CRISPR–Cas9 RNP production, monocyte isolation and electroporation

Primary human monocytes from healthy donors were isolated from leukoreduction chambers after Trima Apheresis (StemCell). Peripheral blood mononuclear cells were isolated by Ficoll centrifugation. Bulk monocytes were subsequently isolated from peripheral blood mononuclear cells by magnetic negative selection using the EasySep Human Monocyte Isolation Kit (StemCell, according to the manufacturer’s instructions). Isolated monocytes were immediately electroporated. Each electroporation reaction consisted of 1 × 10^6^ monocytes, 3.5 μl RNPs and 20 μl electroporation buffer. RNPs were thawed and allowed to come to room temperature. Immediately before electroporation, cells were centrifuged at 400*g* for 5 min, the supernatant was removed by aspiration, and the pellet was resuspended in 20 μl of room-temperature P3 electroporation buffer (Lonza) per reaction. Then, 20 μl of cell suspension was then gently mixed with each RNP and aliquoted into a 96-well electroporation cuvette for nucleofection with the 4D 96-well shuttle unit (Lonza) using pulse code DK-100. Immediately after electroporation,100 μl of prewarmed medium, Iscove’s Modified Dulbecco’s Medium (IMDM) supplemented with 20% HI Human AB Serum (Valley Biomedical), 50 μg ml^−1^ penicillin–streptomycin (Corning) and 1 mM sodium pyruvate (Corning) was added to each well and cells were allowed to rest for 20 min in a 37 °C cell culture incubator. Cells were subsequently replica plated at a density of 1 × 10^6^ per ml in 48-well flat-bottomed culture plates with 500 μl cell suspension per well. Cells were cultured at 37 °C/5% CO_2_ in a humidified cell culture incubator for a total of 7 days before challenge. The full volume of the medium was exchanged after 24 h, and half the volume of medium was exchanged 5 days after electroporation.

#### Immunoblotting

To collect protein lysates for determination of KO efficiency, the medium was removed from each well, cells were washed with 1× PBS (Corning) and 50 μl 2.5× Laemmli sample buffer was added to each well. Cells in sample buffer were heated to 98 °C for 20 min before storage at −20 °C until immunoblotting. Samples and PageRuler Plus Prestained Protein Ladder were thawed and 10 μl of each was loaded onto 4–20% Criterion Tris-HCl SDS-PAGE protein gels (BioRad). Gels were ran at 150 V over 90 min until the ladder was sufficiently separated. Proteins were transferred to polyvinylidene fluoride (PVDF) membranes by methanol-based electrotransfer (BioRad Criterion Blotter) at 90 V for 2 h. Membranes were blocked in 5% milk in PBS and 0.1% Tween-20 overnight before overnight incubation with primary antibodies against CHD1L (E1I8C, Cell Signaling Technologies) and GAPDH (14C10, Cell Signaling Technologies) or β-actin (8H10D10, Cell Signaling Technologies) as a protein loading control. Anti-rabbit or anti-mouse horseradish peroxidase (HRP)-conjugated secondary antibodies (BioRad) were detected using Hyglo HRP detection reagents (Denville Scientific). Blots were incubated in a 1× PBS, 0.2 M glycine, 1.0% SDS, 1.0% Tween-20, pH 2.2 stripping buffer before reprobing.

#### Preparation of HIV concentrated stocks

Replication-incompetent reporter virus stocks were generated with a co-transfection of a VSV-G-pseudotyping vector and a HIV-1 NL4-3 full molecular clone lacking Env with IRES-GFP inserted behind Nef at a ratio of 7.5:2.5. In brief, 10 μg of DNA total was transfected (PolyJet, SignaGen) into 5 × 10^6^ HEK293T cells (ATCC, CRL-3216) according to the manufacturer’s protocol. In total, 25 ml of supernatant was collected at 48 and 72 h and combined. Virus-containing supernatant was filtered through 0.45 mm PVDF filters (Millipore) and virus precipitated in 8.5% polyethylene glycol (PEG, average Mn 6000, Sigma-Aldrich), 0.3 M sodium chloride for 4 h at 4 °C. The supernatants were centrifuged at 3,500 rpm for 20 min and virus resuspended in 0.5 ml PBS for a 100× effective concentration. Aliquots were stored at −80 °C until use.

#### Production of SIV Vpx-containing particles

Virus-like particles containing Vpx (Vpx-VLPs) were produced by co-transfection (PolyJet, SignaGen) of 7.5 μg of an integration-incompetent SIV3+ construct encoding Gag-Pro-Pol plus accessory proteins and 2.5 μg pMD2.G-VSVg into 5 × 10^6^ HEK293T cells (ATCC, CRL-3216) according to the manufacturer’s protocol. Then, 25 ml supernatant was collected at 48 and 72 h and combined. Virus-containing supernatant was filtered through 0.45 mm PVDF filters (Millipore) and precipitated in 8.5% polyethylene glycol (PEG, average Mn 6000, Sigma-Aldrich), 0.3 M sodium chloride for 4 h at 4 °C. The supernatants were centrifuged at 3,500 rpm for 20 min and virus resuspended in 0.5 ml PBS for a 100× effective concentration. The aliquots were stored at −80 °C until use.

#### HIV infection

Seven days after electroporation, each well of monocyte-derived macrophages were pretreated with 5 μl of concentrated Vpx-VLPs in a 20 μl carrier volume to deplete SAMHD1. After 6 h, 2.5 μl of concentrated HIV-1 dEnv nef:IRES:GFP virus stock was added to each well in a 20 μl carrier volume. Cells were cultured in a dark, humidified incubator at 37 °C/5% CO_2_. On days 2 and 5 after infection, the cell culture medium was removed from the cells and stored at −80 °C for p24 quantification by ELISA. Cells were then washed with 1× PBS and Accutase (Sigma-Aldrich) was used to lift the cells from the culture plate according to the manufacturer’s instructions. Cells were fixed in 1% formaldehyde in 1× PBS and stored at 4 °C before flow cytometry.

#### Flow cytometry and analysis of infection data

Flow cytometry analysis was performed on an Attune NxT Acoustic Focusing Cytometer (Thermo Fisher Scientific), recording all events in a 50 μl sample volume after one 80 μl mixing cycle. Data were exported as FCS3.0 files and analysed with a consistent template on FlowJo. In brief, cells were gated for lymphocytes by light scatter followed by doublet discrimination in both side and forward scatter. Cells with equal fluorescence in the BL-1 (GFP) channel and VL-2 (AmCyan) channels were identified as auto-fluorescent and excluded from the analysis. A consistent gate was then used to quantify the fraction of remaining cells that expressed GFP.

#### p24 quantification in the cell supernatants

p24 in the cell supernatants was quantified using an ELISA for HIV-1 Gag p24 (Biotechne R&D Systems) according to the manufacturer’s directions.

### Reporting summary

Further information on research design is available in the Nature Portfolio Reporting Summary linked to this article.

## Extended Data

**Extended Data Fig. 1:**
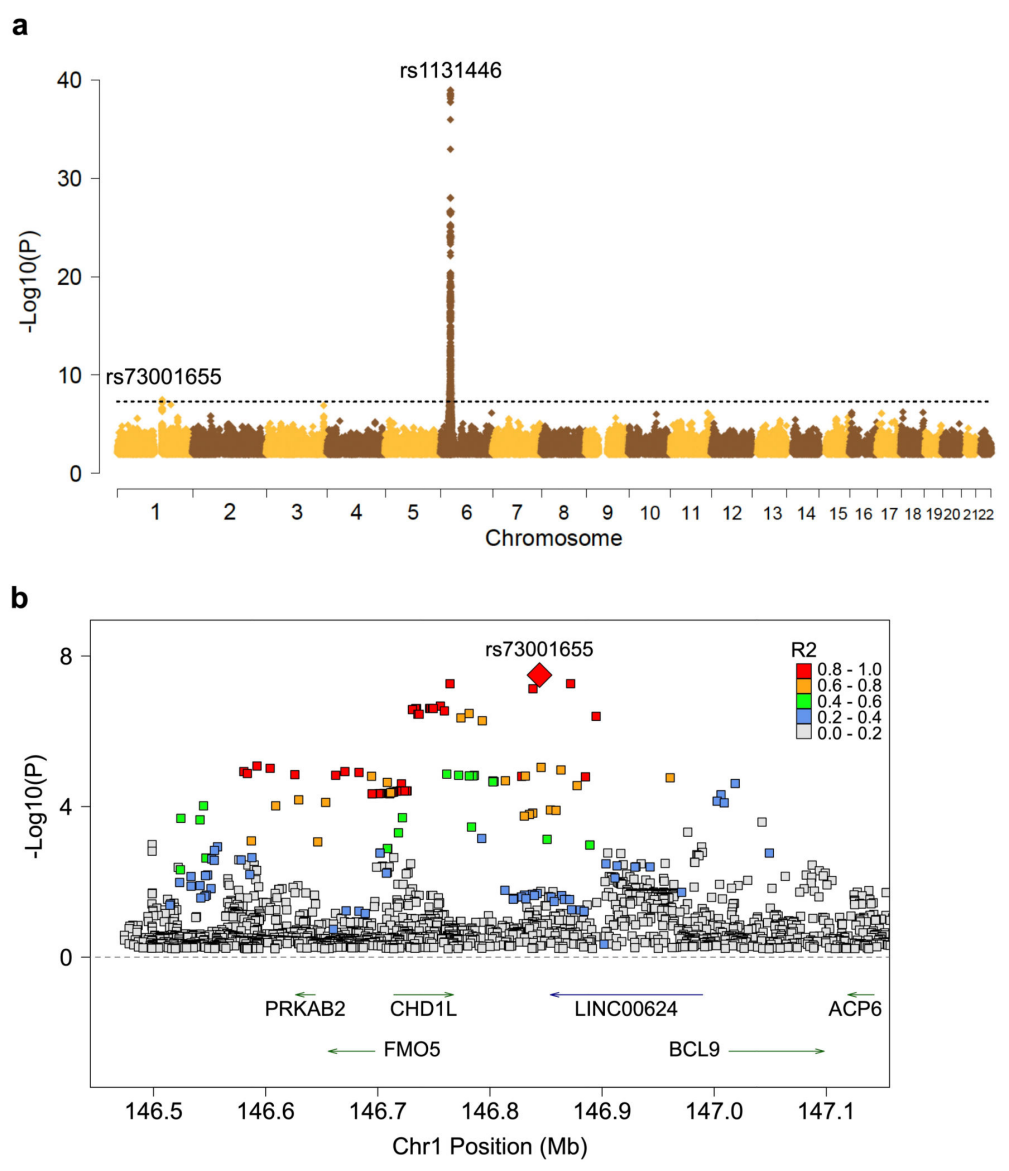
A discovery genome-wide association analysis identifies a potentially novel locus associated with HIV spVL in individuals with African ancestries. **a**, Genome-wide association results of the impact of common polymorphisms on HIV-1 spVL in the discovery set of 2,682 individuals of African ancestry. Genetic variants (yellow/brown diamonds) are plotted by chromosome position (GRCh37, x-axis) and statistical significance (y-axis). The dashed line indicates the screening threshold for significance (*P* < 5 × 10^−8^). Variants in two genomic regions, the HLA region on chromosome 6 and a novel chromosome 1 locus, are significantly associated with spVL. The top associated variant per region is listed above the association peak. **b**, Association results across the newly identified chromosome 1 region in the discovery sample of 2,682 individuals of African ancestry. Variants (boxes and diamond) are plotted by position (GRCh37) and –log_10_(P). The top associated variant, rs73001655 (P = 3.2 × 10^−8^) is represented by the red diamond. Association was calculated per group using linear regression and meta-analysed across groups. Additional variants are coloured by their correlation to rs73001655 calculated from the African subset of the 1000 Genomes Project reference phase 3 sample. Arrows below the dashed line indicate the location and direction of transcription of protein-coding genes (green) and non-coding RNA (blue).

**Extended Data Fig. 2:**
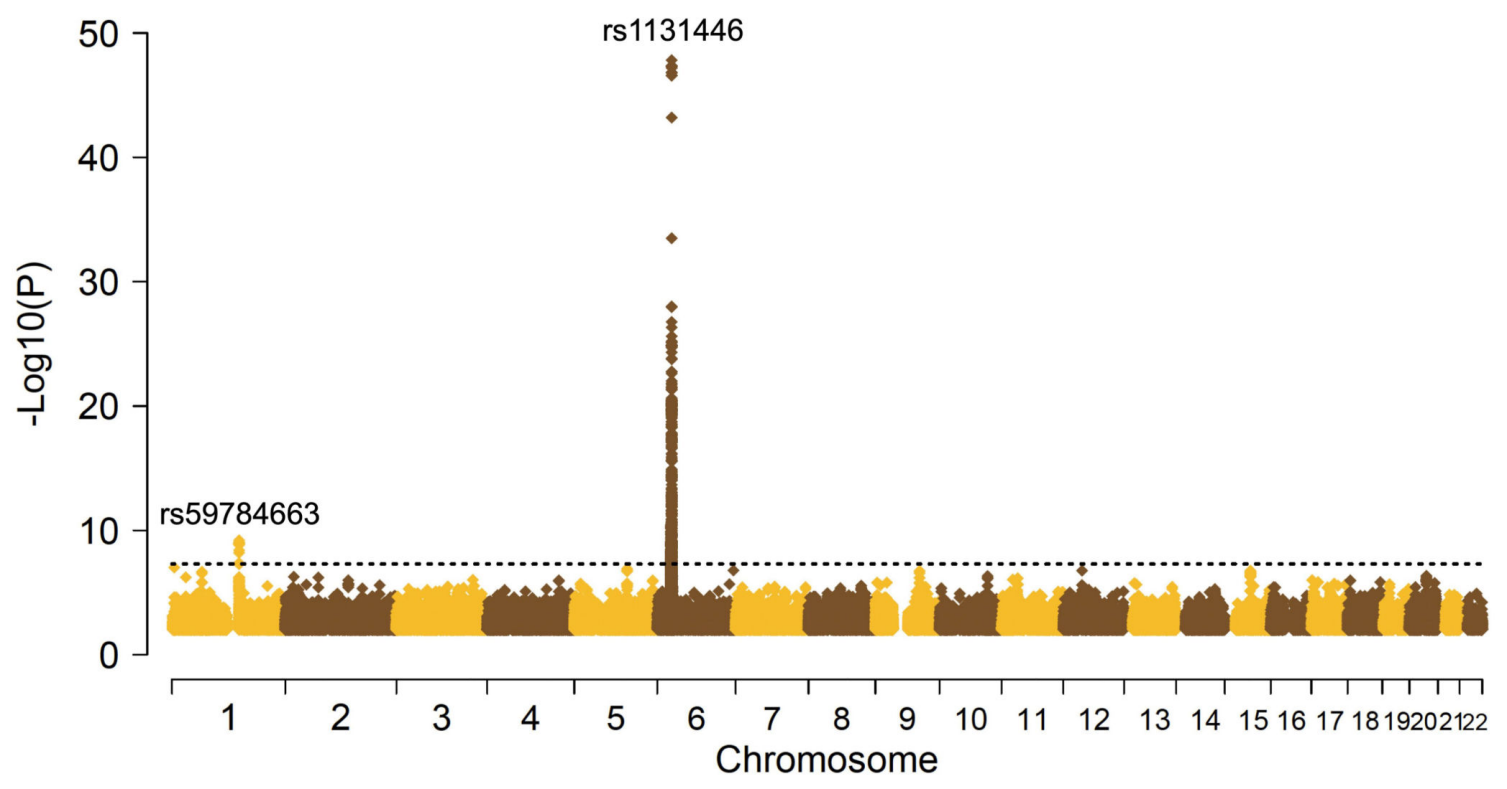
Genome-wide association results of the impact of common polymorphisms on HIV-1 spVL in the combined set of 3,879 individuals with African ancestries. Genetic variants (yellow/brown triangles) are plotted by chromosome position (GRCh37, x-axis) and statistical significance (–log_10_(P), y-axis). The dashed line indicates the threshold for genome-wide significance in samples with African ancestries (*P* < 5 × 10^−9^). Variants in two regions are significantly associated with spVL. The top associated variant per region is listed above the association peak.

**Extended Data Fig. 3:**
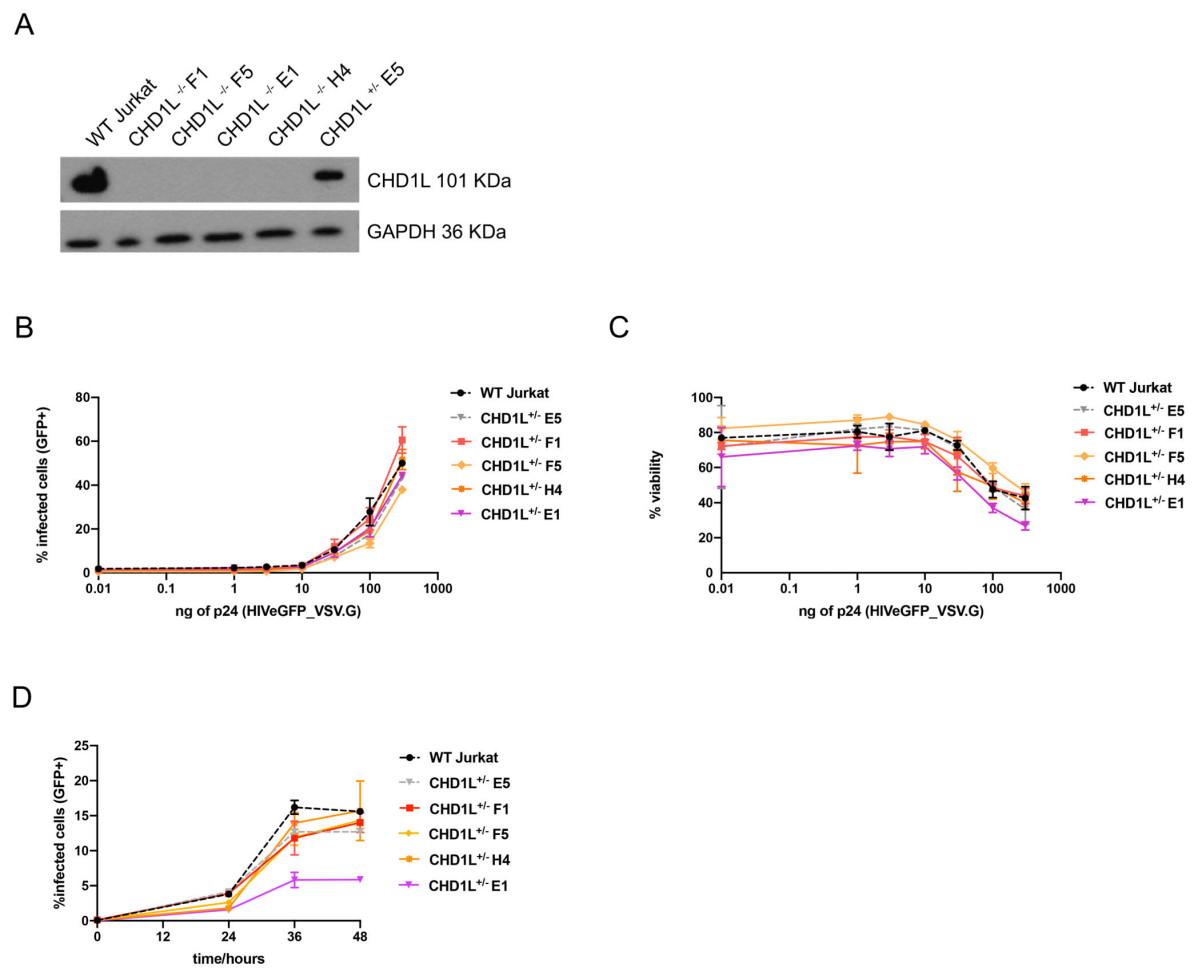
Characterization and infection assays in Jurkat *CHD1L* mono and biallelic knockout mutants. **a**, Western blot for CHD1L shows reduced (E5) and ablated (F1, F5, E1, H4) CHD1L expression, consistent with the respective genotypes. Levels of GAPDH are shown as loading control. **b**,**c**, The percentage of GFP positive cells (**b**) and viable cells (**c**) in *CHD1L* knockout clones was evaluated by flow cytometry at 48 h post-infection with different concentrations of NL4-3-deltaEnv-GFP/VSV-G (0-300 ng of p24). **d**, The percentage of GFP positive cells was evaluated at different time points (24, 36, 48 h) post-transduction with 300ng of p24 NL4-3-deltaEnv-GFP/ VSV-G virus.

**Extended Data Fig. 4:**
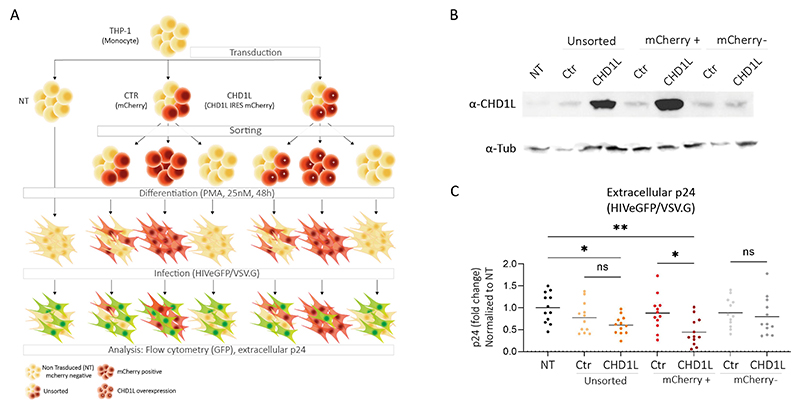
Impact of CHD1L overexpression on HIV replication in THP-1 differentiated cells. **a**, Experimental design. THP-1 were transduced with lentiviral particles encoding, either CHD1L IRES mcherry (CHD1L), or mCherry alone as a control (CTR), or left untreated (NT). Successfully transduced cells were sorted by FACS. The resulting sorted monocyte populations were differentiated into macrophages during 48 h in presence of 25 nM PMA and let recover for 24 h additional hours. Differentiated cell lines were infected with the single-round amphotropic HIVeGFP/VSV.G virus. **b**, Western blot confirming CHD1L overexpression in THP-1 cells transduced with CHD1L-encoding vector. **c**, Extracellular p24 was measured by ELISA at day 3 post-infection (n = 4). Results are normalized to the NT sample at day 3, mean and individual values of at least two experiments in triplicate are plotted. Multiple comparison One-way ANOVA showed statistical significance between CTR and CHD1L overexpressing cells (p < 0.005).

**Extended Data Fig. 5:**
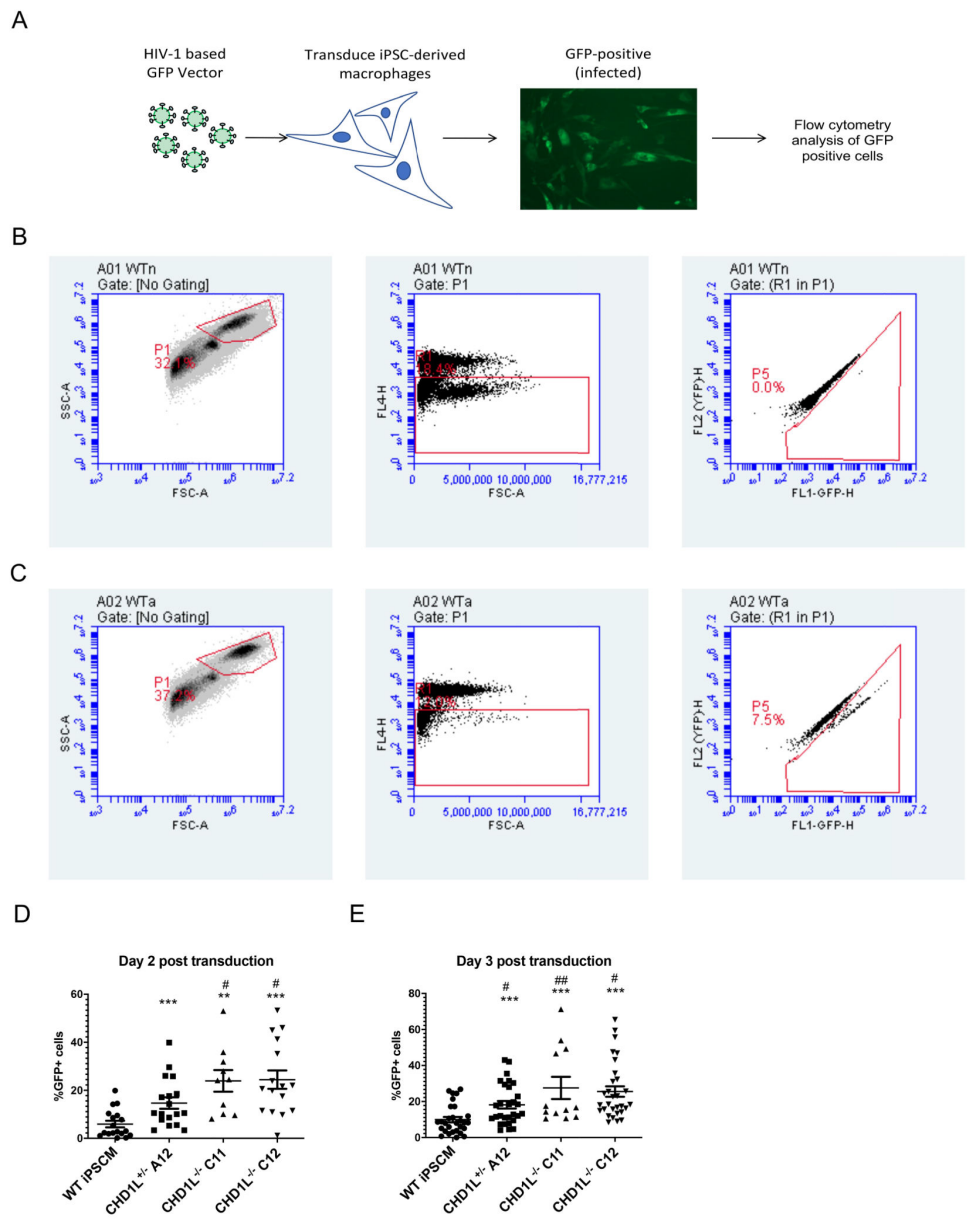
Infection of iPSC-derived macrophages (iPSDMs) with the HIV-1 vector, NL4-3-deltaEnv-GFP/VSV-G. **a**, Experimental design: VSV-G pseudotyped HIV-1 vector was used to infect iPSDMs. Viral activity was assessed by GFP expression through flow cytometry analysis. **b**,**c**, Gating strategy for uninfected (**b**) and infected (**c**) WT cells of a single experiment. Live cells were selected by light scattering exclusion of debris (left panels) and dead cells exclusion by DRAQ-7 staining (middle panels). To circumvent autofluorescence, GFP-positivity was controlled through FL1/FL2 comparison (right panels). **d**,**e**, Raw infection data for WT and CHD1L knockout iPSDMs. Data refer to Fig. 4c and d of the main text. Data from individual wells of each experiment are reported as raw percentage of GFP positive cells. *, ** and *** represent statistically significant differences (p ≤ 0.05, 0.01 and 0.001, respectively) between WT and mutant clones using Wilcoxon matched-pairs signed rank test. ^#, ##^ represent statistically significant differences (p ≤ 0.05 and 0.01, respectively) between the CHD1L+/− A12 clone and the CHD1L−/− C12 and C11 clones using Wilcoxon matched-pairs signed rank test.

**Extended Data Fig. 6:**
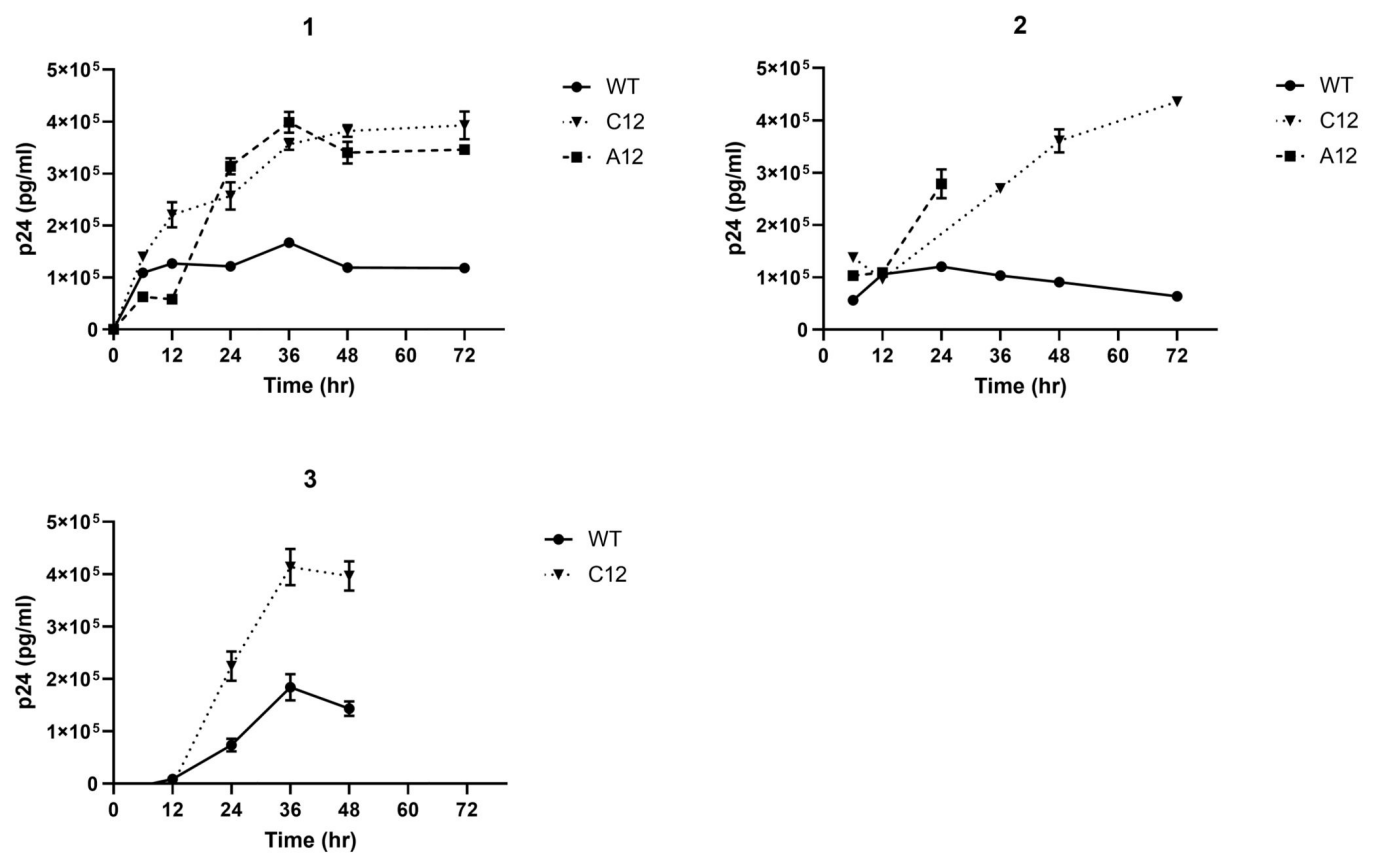
Viral Gag particle release from WT and CHD1L knockout macrophages. Viral Gag particle release was measured by p24 ELISA assay on the culture supernatants at different time points post-transduction. The three graphs show independent biological replicates. A12 cells were not available for all time points. Data are reported as the average and standard deviation of duplicate p24 ELISA readings. In each independent replicate, C12 was significantly different from WT as determined by repeated measures ANOVA (1: F (6, 12) = 188.8, P < 0.0001, 2: F (5, 10) = 503.6, P < 0.0001, 3: F (5, 10) = 81.58, P < 0.0001).

**Extended Data Fig. 7:**
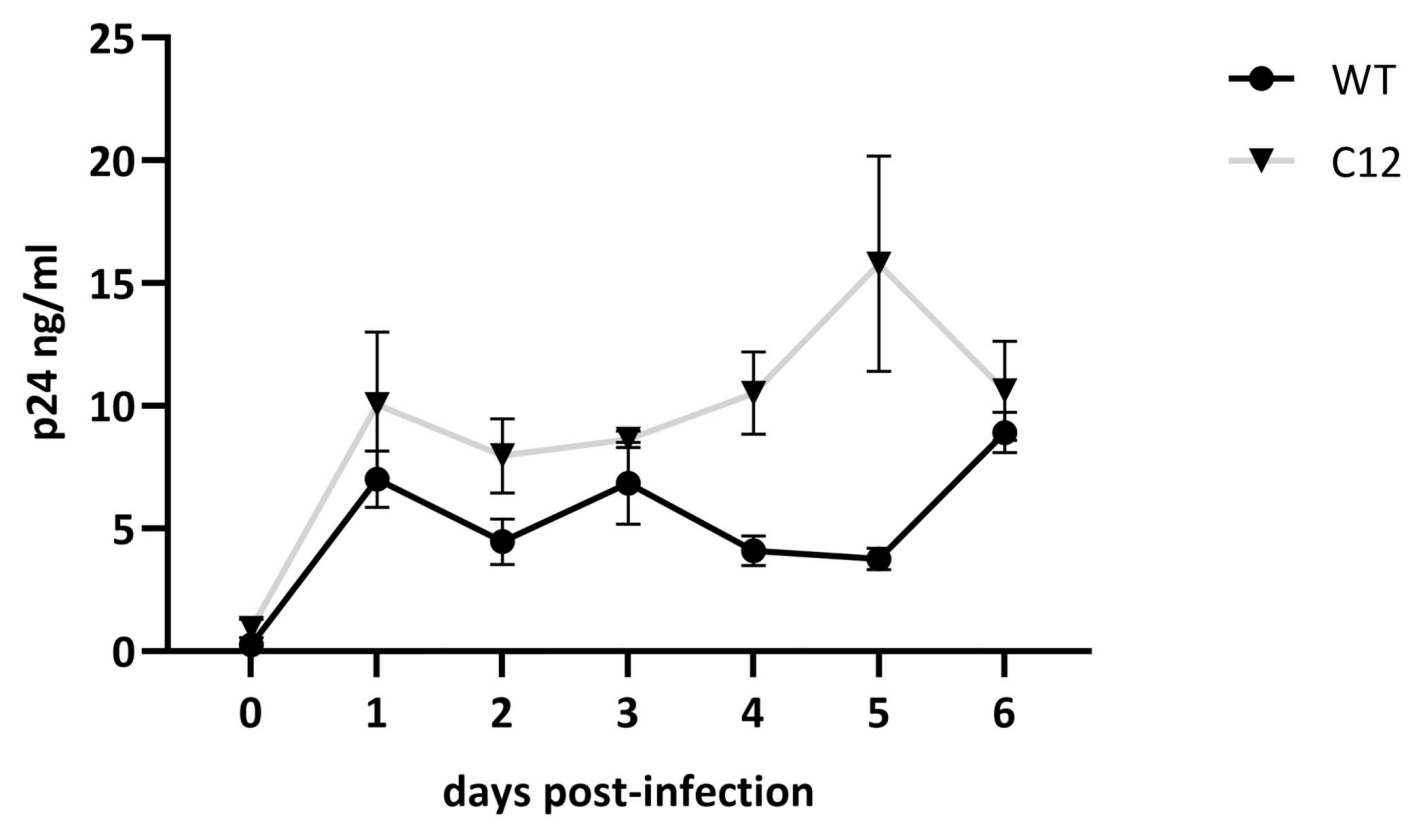
p24 release from CHD1L KO cells infected with replication competent HIV.BE_GIN. Raw supernatant p24 values corresponding to Fig. 4h in the main text.

**Extended Data Fig. 8:**
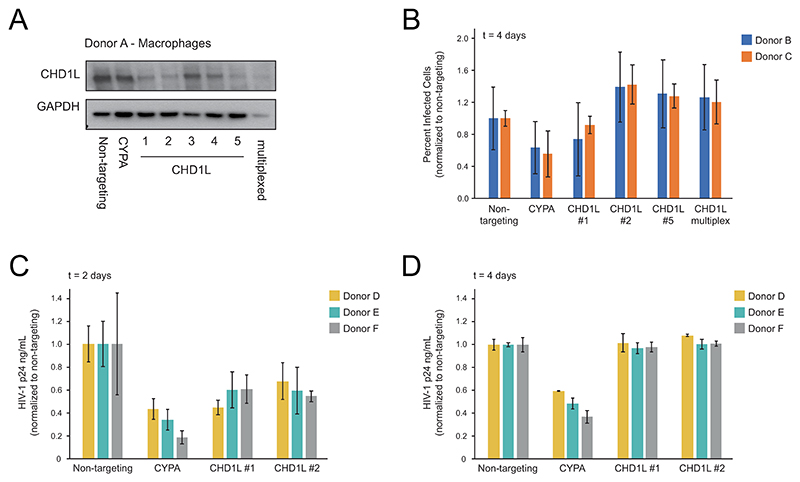
Assessing the impact of CHD1L knock-out in primary monocyte-derived macrophages on HIV infection. **a**, CHD1L was efficiently knocked out in primary MDMs by 3 of 5 crRNP constructs and a combined, multiplexed pool. **b**, Percent infected cells 4 days post-challenge as measured by flow cytometry showed an increase in three of the four CHD1L knockout pools compared to the non-targeting control, but these differences were not statistically significant. **c**,**d**, p24 levels in the culture supernatants as measured by ELISA were lower in CHD1L knockout cell pools 2 days post-infection (**c**), but recovered to the level of the non-targeting control by 4 days post-infection (**d**).

**Extended Data Table 1:** Association result for genome-wide significant variants on chromosome 1 influencing HIV-1 spVL in individuals of African ancestries in the discovery, replication and combined samples

			Discovery (n=2,682)			Replication (n=1,197)			Combined (n=3,879)		
Variant ID	Alt	Ref	β	se	P	β	se	P	β	se	P
rs59784663	G	A	-0.31	0.06	7.4×10^-8^	-0.28	0.09	2.3×10^-3^	-0.30	0.05	6.4×10^-10^
rs72999655	G	A	-0.30	0.06	2.1×10^-7^	-0.30	0.09	1.1×10^-3^	-0.30	0.05	9.1×10^-10^
rs72999634	G	A	-0.29	0.06	2.7×10^-7^	-0.30	0.09	9.9×10^-4^	-0.30	0.05	9.9×10^-10^
rs7526114	A	G	-0.30	0.06	2.5×10^-7^	-0.30	0.09	1.1×10^-3^	-0.30	0.05	1.0×10^-9^
rs72999646	A	T	-0.30	0.06	2.5×10^-7^	-0.30	0.09	1.1×10^-3^	-0.30	0.05	1.0×10^-9^
rs72999637	C	T	-0.30	0.06	2.5×10^-7^	-0.30	0.09	1.1×10^-3^	-0.30	0.05	1.0×10^-9^
rs72999648	A	G	-0.30	0.06	2.5×10^-7^	-0.30	0.09	1.1×10^-3^	-0.30	0.05	1.0×10^-9^
rs7535451	G	A	-0.30	0.06	2.5×10^-7^	-0.30	0.09	1.1×10^-3^	-0.30	0.05	1.0×10^-9^
rs59213667	A	G	-0.30	0.06	2.5×10^-7^	-0.30	0.09	1.1×10^-3^	-0.30	0.05	1.0×10^-9^
rs73001655	A	G	-0.30	0.05	3.2×10^-8^	-0.22	0.08	6.9×10^-3^	-0.28	0.05	1.0×10^-9^
rs72999638	A	T	-0.30	0.06	2.5×10^-7^	-0.30	0.09	1.1×10^-3^	-0.30	0.05	1.0×10^-9^
rs72999656	T	C	-0.30	0.06	2.9×10^-7^	-0.30	0.09	1.1×10^-3^	-0.30	0.05	1.2×10^-9^
rs77029719	G	C	-0.30	0.05	5.4×10^-8^	-0.23	0.08	6.0×10^-3^	-0.28	0.05	1.4×10^-9^
rs72999639	C	T	-0.29	0.06	3.5×10^-7^	-0.30	0.09	1.1×10^-3^	-0.30	0.05	1.4×10^-9^
rs72999640	T	C	-0.29	0.06	3.5×10^-7^	-0.30	0.09	1.1×10^-3^	-0.30	0.05	1.4×10^-9^
rs73004025	T	C	-0.30	0.06	4.0×10^-7^	-0.28	0.09	2.7×10^-3^	-0.29	0.05	4.0×10^-9^

## Supplementary Material

Supplementary Material

## Figures and Tables

**Fig. 1:**
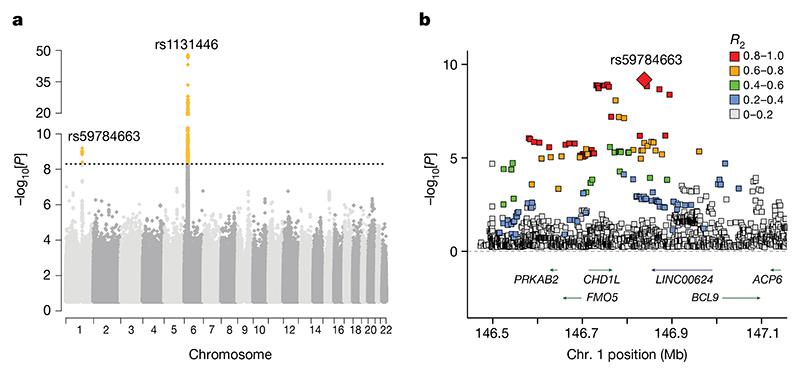
GWAS analysis identifies a locus on chromosome 1 that is associated with HIV spVL in individuals with African ancestries. **a**, Genome-wide association results of the impact of common polymorphisms on HIV-1 spVL in the combined set of 3,879 individuals of African ancestries. Genetic variants were tested for association with spVL per group using linear regression and evidence was combined across groups using inverse-variance weighted meta-analysis in a fixed-effects framework. Genetic variants (grey and yellow diamonds) are plotted by chromosome position (GRCh37, *x* axis) and statistical significance (−log_10_[*P*], *y* axis). The dashed line indicates the threshold for genome-wide significance in populations of African ancestries (*P* < 5 × 10^−9^). Variants in two regions are significantly associated with spVL (yellow diamonds). These include the HLA region on chromosome 6 and a locus on chromosome 1. The top associated variant per region is listed above the association peak. To improve visualization of the chromosome 1 region, the *y* axis was split. Full genome-wide results are presented in Extended Data Fig. 2. **b**, Association results across the newly identified chromosome 1 region. Variants (boxes and diamond) are plotted by position (GRCh37) and –log_10_[*P*]. The most strongly associated variant, rs59784663 (*P* = 6.4 × 10^−10^), is represented by a red diamond. Additional variants are coloured by their correlation with rs59784663 calculated from the African subset of the 1000 Genomes Project reference phase 3 sample. The arrows below the dashed line indicate the location and direction of transcription of protein-coding genes (green) and non-coding RNA (blue).

**Fig. 2:**
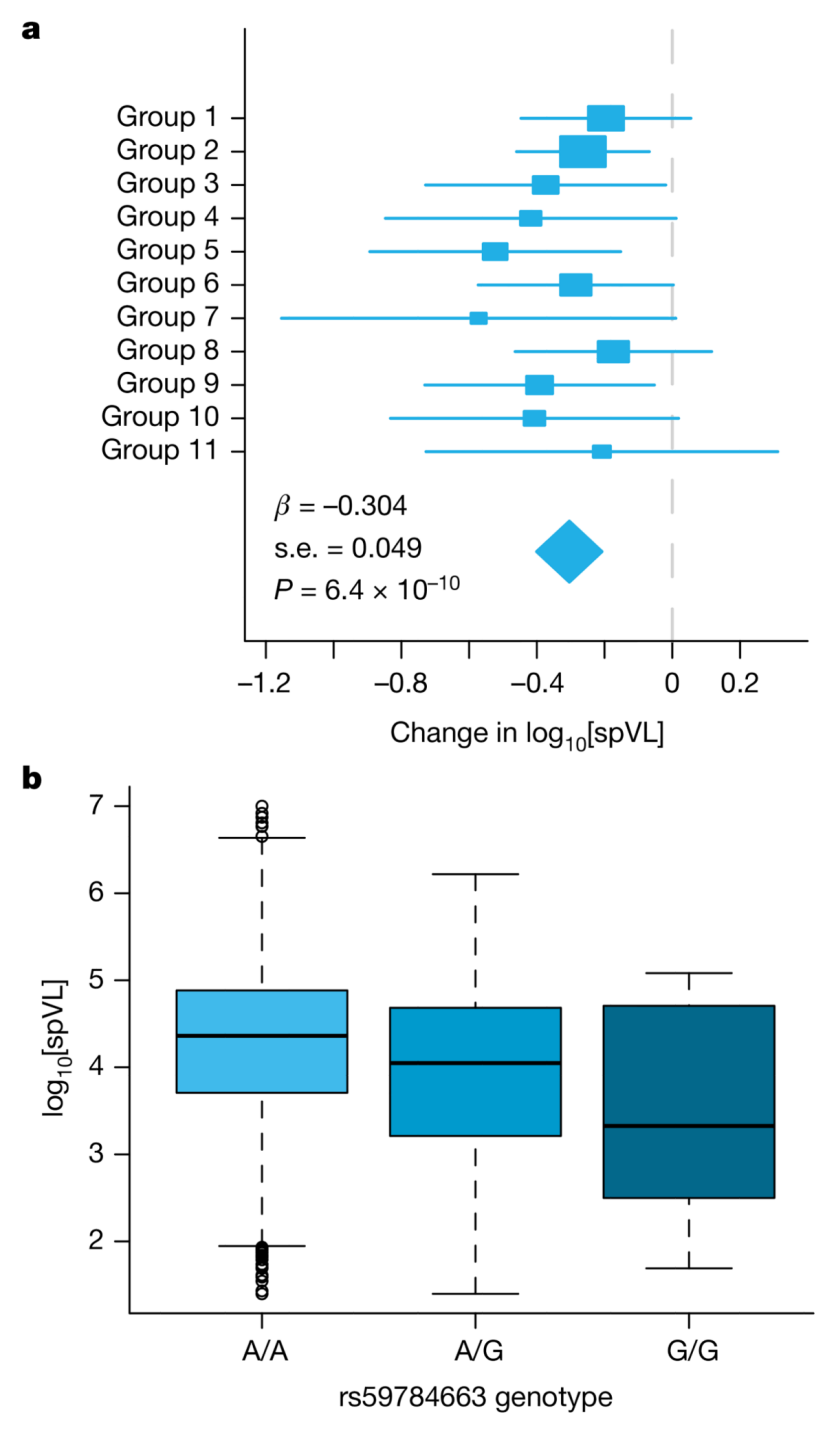
The viral-load-decreasing effect of rs59784663(G). **a**, The per group effect of rs59784663(G) on HIV-1 spVL. The boxes indicate the per-group effect estimate (*β* from linear regression) with s.e. (whiskers). The summary effect (diamond) and *P* value are the result of meta-analysis across groups. Results from the meta-analysis are shown; the *β*, s.e. and *P* value are given at the bottom left. The relationship between group number, cohort/clinical centre and ancestry is presented in Supplementary Table 1. **b**, The additive allelic effect of the rs59784663 genotype on spVL is summarized across all individuals.

**Fig. 3:**
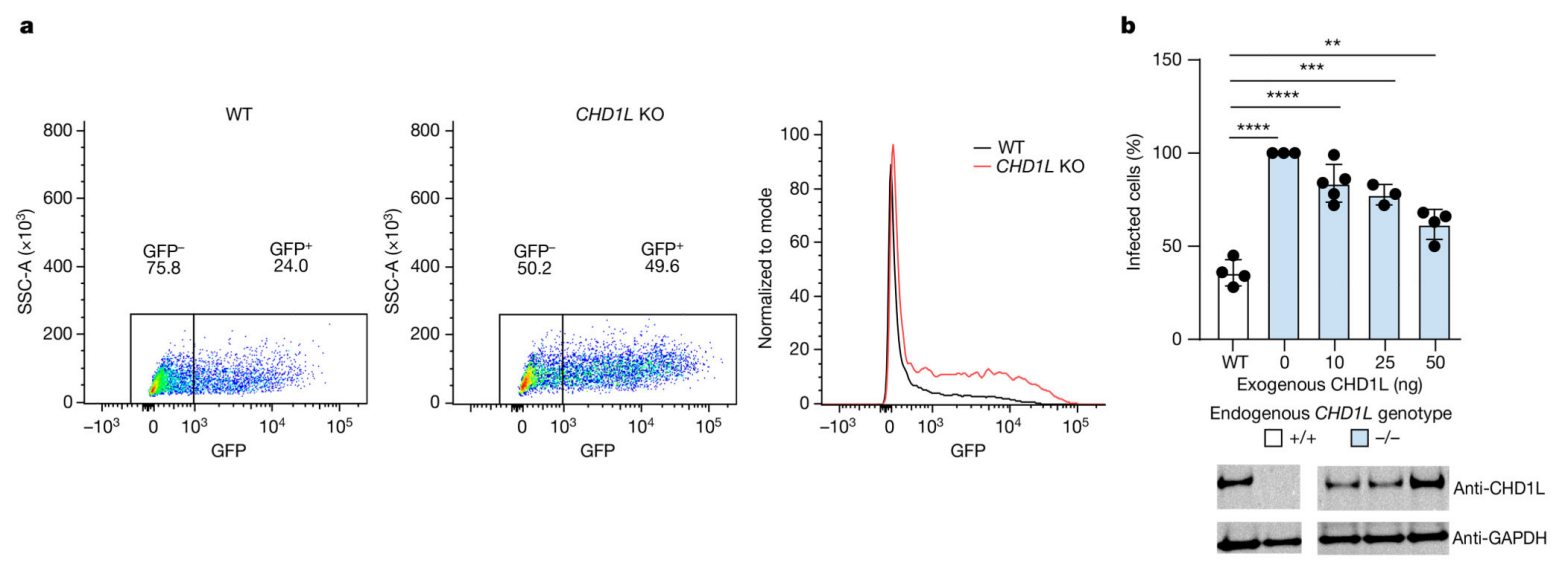
CHD1L expression decreases single-round HIV infection in U2OS cells. **a**, Representative flow cytometry plots of WT and *CHD1L*-KO U2OS cells infected with HIV NL4.3-deltaEnv-GFP/VSV-G. After 48 h, *CHD1L-*WT U2OS cells (left) showed an approximately twofold reduction in number of GFP-positive cells compared with *CHD1L-*KO cells (middle, overlaid on the right). **b**, U2OS *CHD1L*-KO cells were transfected with increasing concentrations (0, 10, 25 or 50 ng) of CHD1L expression plasmid. Then, 24 h after transfection, WT U2OS or transfected KO cells were infected with HIV NL4.3-deltaEnv-GFP/VSV-G and assessed for GFP positivity after 48 h. A representative immunoblot for CHD1L and GAPDH is shown below. *n* ≥ 4. Statistical analysis was performed using unpaired *t*-tests; ****P ≤ 0.0001, ***P ≤ 0.0005, **P ≤ 0.005. The full blot is provided in Supplementary Fig. 8.

**Fig. 4:**
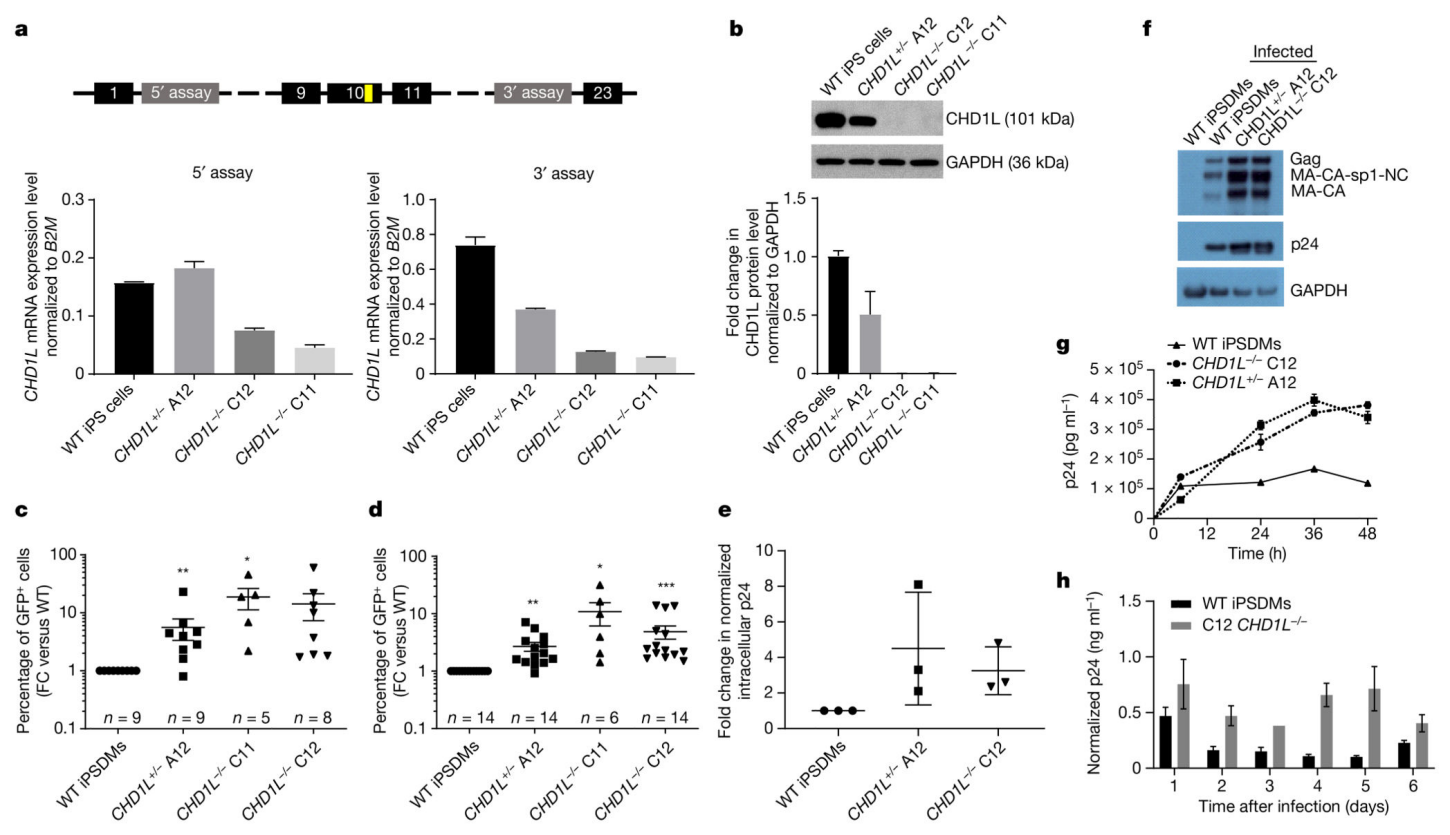
KO of *CHD1L* increases HIV-1 infection in human-iPS cell-derived macrophages. **a**, Quantitative PCR (qPCR) validation of the level of full-length *CHD1L* mRNA after induction of frameshift deletions. The diagram shows the position of the 5′ and 3′ TaqMan assays used to confirm exon 10 KO (puromycin resistance cassette in yellow). The bar plots show the relative expression of *CHD1L* normalized to *B2M* expression for WT and heterozygous and homozygous KO clones. **b**, Immunoblot analysis of CHD1L and GAPDH protein in WT and KO clones. The blot is representative of three replicates (all data are shown in Supplementary Fig. 10). The fold changes in normalized CHD1L levels are given below. *n* = 3. Data are mean ± s.d. **c**,**d**, The percentage of GFP-positive cells in HIV-1-GFP-transduced WT and *CHD1L*-KO clones evaluated 2 days (**c**) and 3 days (**d**) after transduction, normalized to the WT. Each point represents an independent experiment, conducted in duplicate. Statistical analysis was performed using Wilcoxon matched-pairs signed-rank tests, comparing between the WT and mutant clones; *P ≤ 0.05, **P ≤ 0.01, ***P ≤ 0.001. **e**, Intracellular viral capsid p24 protein measured by enzyme-linked immunosorbent assay (ELISA) 2 days after transduction. *n* = 3. **f**, Western blot analyses of intracellular viral capsid p24 2 days after transduction. iPSDMs, iPS cell-derived macrophages. **g**, Time-dependent extracellular viral Gag particle release measured by p24 ELISA. Data are mean ± s.d. of duplicate readings and are representative of three independent experiments (all data are shown in Extended Data Fig. 6). **h**, p24 production after infection of *CHD1L-*WT and *CHD1L*-KO cells with HIV.BE_GIN (BaL Env and GFP-IRES-nef) replication-competent virus. The graphs show p24 (ng ml^−1^) normalized to the GFP-positive cell proportion for 2–4 replicates (except for C12 at day 3, for which *n* = 1). Data are mean ± s.e.m. A significance value of *P* ≤ 0.005 was determined for normalized WT/C12 comparisons using a paired *t*-test. Raw p24 values are shown in Extended Data Fig. 7.

## Data Availability

Access to individual-level genotyping data is restricted to investigators from institutions that join the International Collaboration for the Genomics of HIV (ICGH) by signing the ICGH collaboration agreement, which is obtainable on request (jacques.fellay@epfl.ch). Owing to the highly sensitive nature of the HIV diagnostic of all study participants, the risk associated with potential re-identification was deemed to be very high by the IRBs, preventing broader sharing of individual-level data. The GWAS summary statistics are deposited in the NHGRI-EBI Catalog of human genome-wide association studies (https://www.ebi.ac.uk/gwas/home) under accession number GCST90269914. RNA-seq data are available at NCBI (PRJEB18581) and the eQTL results are available at GitHub (https://github.com/smontgomlab/AFGR).
