# Supplementary material for: Africa-specific human genetic variation near CHD1L associates with HIV-1 load
Source: Nature. Author manuscript; Available in PMC 2025 Jan 22. (PMC10848312; doi:10.1038/s41586-023-06370-4)
Supplement: Supplementary Material [file EMS202086-supplement-Supplementary_Material.pdf]

nature portfolio

https://doi.org/10.1038/s41586-023-06370-4

Supplementary information

Africa-specific human genetic variation near *CHD1L* associates with HIV-1 load

In the format provided by the
authors and unedited

Africa-specific human genetic variation near CHD1L associates with HIV-1 load

Supplementary materials

Supplementary figures S1 to S12

Supplementary Tables S1 to S12

Supplementary Note 1 & 2

Table of Contents

		Page
**Figures**		
Figure S1	Per group principal components plots of the African sample of the International Collaboration for the Genomics of HIV	5
Figure S2	Histograms of spVL per-group and combined	10
Figure S3	Quantile-quantile plots of genome-wide association results per genotype group	12
Figure S4	No association between genetic variants in the chromosome 1 region identified in the African sample and spVL in 6,315 HIV-1 infected individuals of European ancestry	14
Figure S5	Correlation of p-values across the chromosome 1 variants between different models	15
Figure S6	Colocalization plots and colocalization posterior probabilities testing for a relationship between variant impact on spVL and expression of *CHD1L* and *LINC00624* in LCLs	16
Figure S7	Scatterplot of PAINTOR posterior probabilities for variants located in the chromosome 1 associated region	17
Figure S8	Expression of CHD1L in U2OS rescue experiments	18
Figure S9	Detection of CHD1L in U2OS, T and myeloid cell lines	19
Figure S10	Generation of iPSC CHD1L mono and biallelic knockout mutants	20

Figure S11	Boxplot representation of the FPKM values for the full length *CHD1L* transcript for each clone	22
Figure S12	CHD1L knockout efficiency in iPSCs and differentiation into macrophages	23

Tables

Table S1	Studies included in the genome-wide meta-analysis of host control of HIV-1 spVL	25
Table S2	Correlation between rs1131446-T and classical class I HLA alleles and variable amino acid positions	26
Table S3	Frequency of rs73001655-A in the 1000Genomes super populations	27
Table S4	Frequency of rs73001655-A in the 1000Genomes African sub-populations	28
Table S5	Frequency of rs59784663-G in the 1000Genomes African sub-populations	29
Table S6	Frequencies of genome-wide significant chromosome 1 variants in the 1000 Genomes super-populations	30
Table S7	Association statistics for rs59784663-G from sensitivity analyses using linear mixes models and accounting for inferred local ancestry	31
Table S8	Comparison of association results for variants within the chromosome 1 associated region (Mb146.4 – 147.4) between HIV-1 spVL and haematological traits	32

Table S9	Populations and sample sets included in the eQTL analysis. For each population the number variants tested in the Chromosome 1 region is listed	34
Table S10	Variants making up the 95% credible set identified using PAINTOR	35
Table S11	PCR primers and CRISPR sequences utilized in generation and genotyping of CHD1L-/- iPSC mutant clones	37
Table S12	PCR primers and CRISPR sequences utilized in generation and genotyping of CHD1L-/- Jurkat E6.1 mutant clones	38

Supplementary Note

Note S1	Characterization of Jurkat CHD1L-/- clones and infection assays	40
Note S2	Cohorts and Individuals contributing to the International Consortium for the Genomics of HIV	42

Fig S1

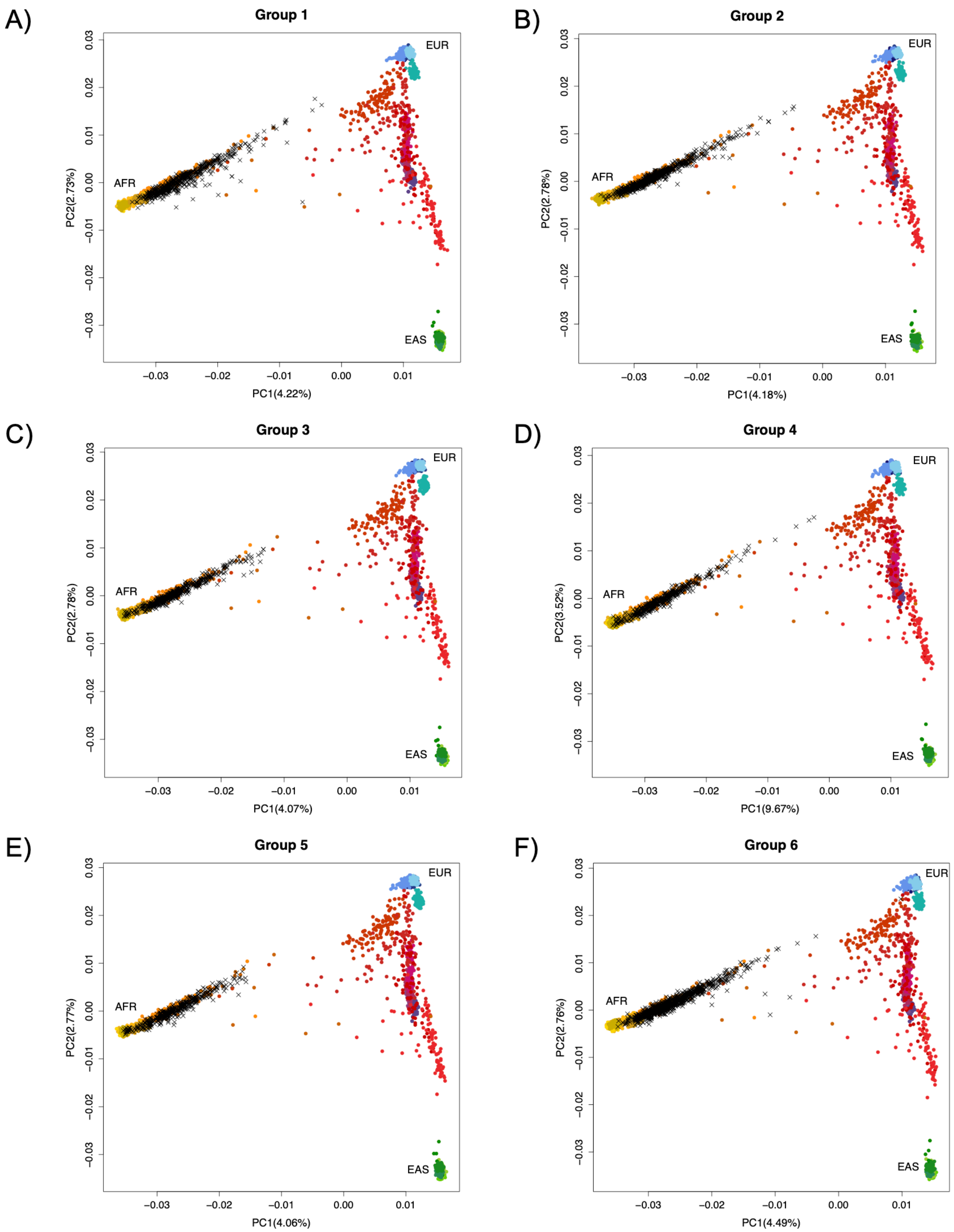
A)
Group 1
PC1(4.22%)
PC2(2.73%)
B)
Group 2
PC1(4.18%)
PC2(2.78%)
C)
Group 3
PC1(4.07%)
PC2(2.78%)
D)
Group 4
PC1(9.67%)
PC2(3.52%)
E)
Group 5
PC1(4.06%)
PC2(2.77%)
F)
Group 6
PC1(4.49%)
PC2(2.76%)
AFR
EUR
EAS
−0.03
−0.02
−0.01
0.00
0.01
0.02
0.03

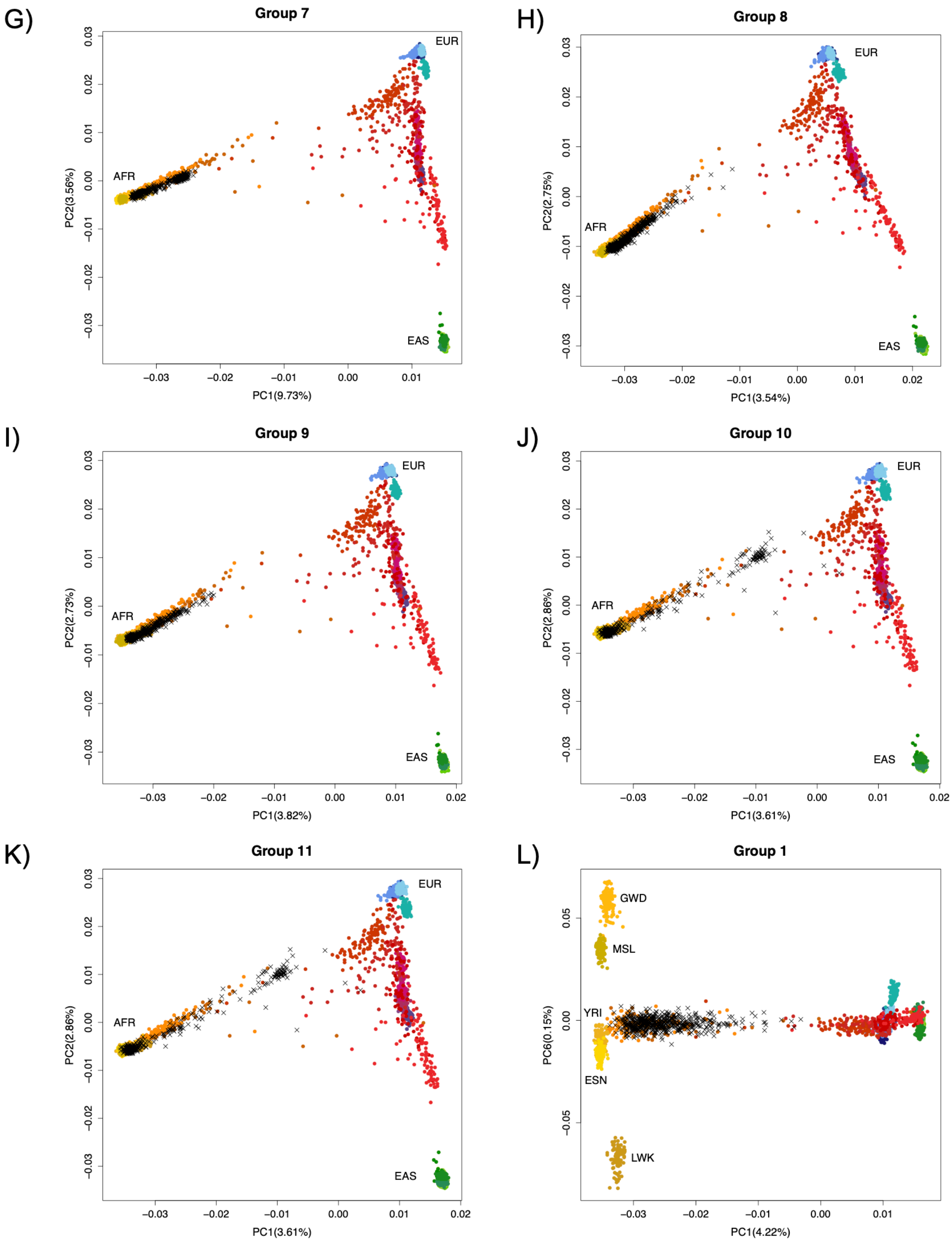
G)
Group 7
EUR
AFR
EAS
PC1(9.73%)
PC2(3.56%)
H)
Group 8
EUR
AFR
EAS
PC1(3.54%)
PC2(2.75%)
I)
Group 9
EUR
AFR
EAS
PC1(3.82%)
PC2(2.73%)
J)
Group 10
EUR
AFR
EAS
PC1(3.61%)
PC2(2.86%)
K)
Group 11
EUR
AFR
EAS
PC1(3.61%)
PC2(2.86%)
L)
Group 1
GWD
MSL
YRI
ESN
LWK
PC1(4.22%)
PC6(0.15%)

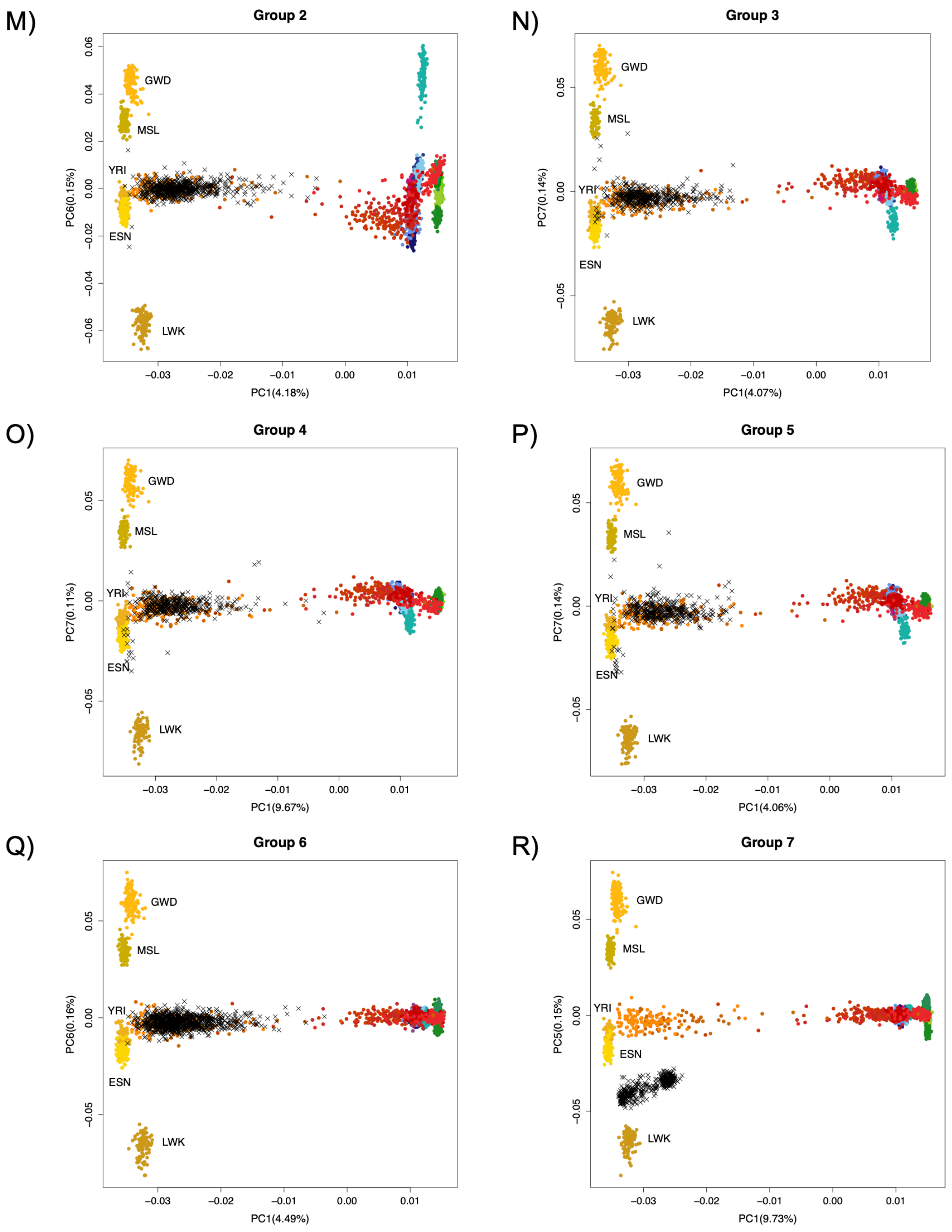
M)
Group 2
GWD
MSL
YRI
ESN
LWK
PC6(0.15%)
PC1(4.18%)
N)
Group 3
GWD
MSL
YRI
ESN
LWK
PC7(0.14%)
PC1(4.07%)
O)
Group 4
GWD
MSL
YRI
ESN
LWK
PC7(0.11%)
PC1(9.67%)
P)
Group 5
GWD
MSL
YRI
ESN
LWK
PC7(0.14%)
PC1(4.06%)
Q)
Group 6
GWD
MSL
YRI
ESN
LWK
PC6(0.16%)
PC1(4.49%)
R)
Group 7
GWD
MSL
YRI
ESN
LWK
PC5(0.15%)
PC1(9.73%)

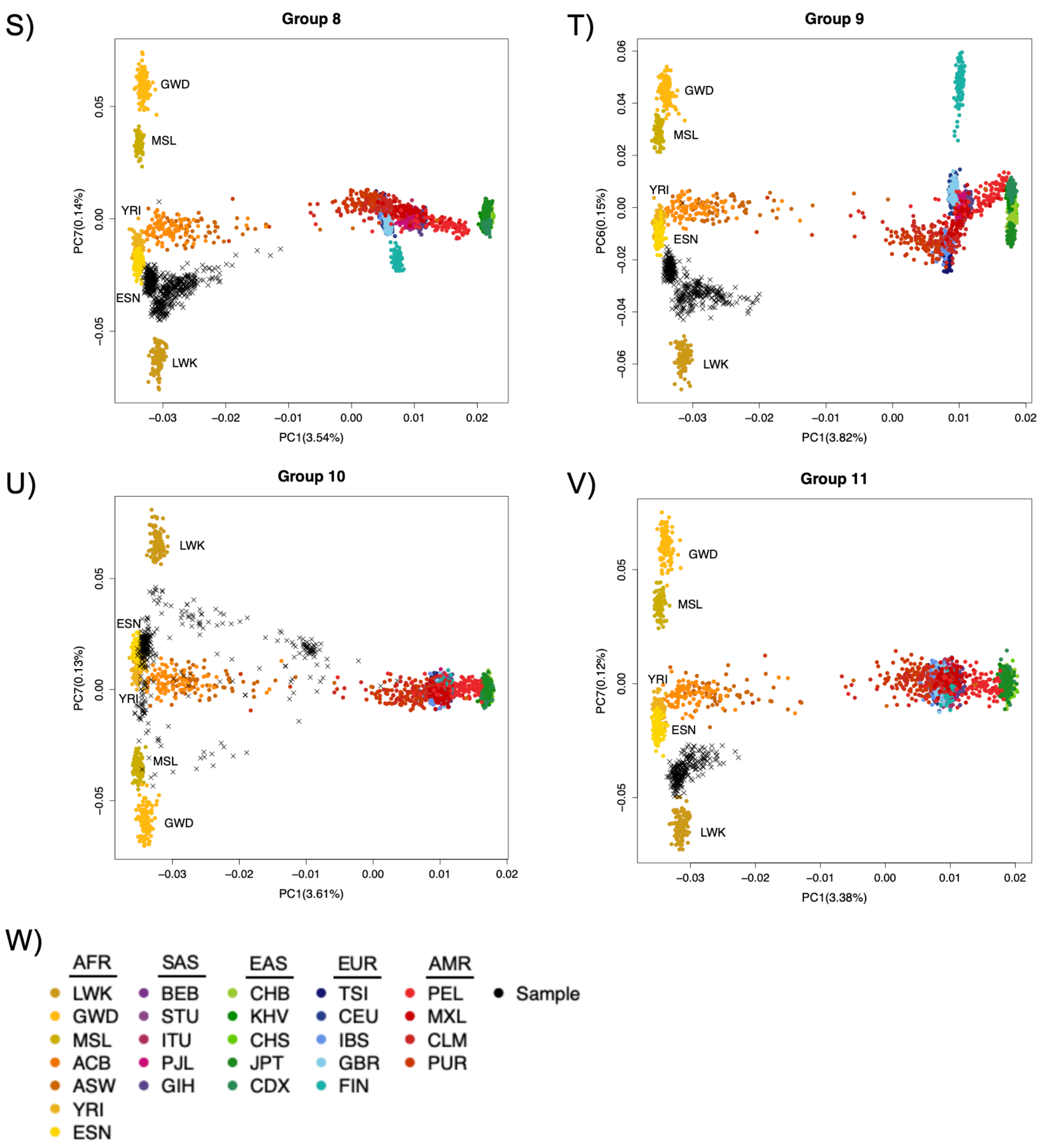

Figure S1. Per group principal components plots of the African sample of the International Collaboration for the Genomics of HIV (black circles) combined with the 1000 Genomes Project reference data (coloured circles). A-K) PC1 vs PC2 shows distribution of samples across the top components. The major continental population

groups (AFR, ASN, EUR) are labeled for clarity. L-V) Shows distribution of samples across the top component (PC1) and the component which separates out the 1000 Genomes African samples per group. The African sub-populations (ESN, GWD, LWK, MSL, YRI) are labeled for clarity. Principal components were calculated combining 1000 Genomes Phase 3 data with the ICGH sample data over a set of high quality shared genetic variants using EIGENSTRAT. Colour legend (W) is grouped by 1000 Genomes super populations, i.e. African (AFR), South Asian (SAS), East Asian (EAS), European (EUR) and Admixed American (AMR). Sub-population abbreviations: Luhya in Webuye, Kenya (LWK), Gambian in Western Divisions in the Gambia (GWD), Mende in Sierra Leone (MSL), African Caribbean's in Barbados (ACB), Americans of African Ancestry in South West USA (ASW), Yoruba in Ibadan, Nigeria (YRI), Esan in Nigeria (ESN), Bengali from Bangladesh (BEB), Sri Lankan Tamil from the UK (STU), Indian Telugu from the UK (ITU), Punjabi from Lahore, Pakistan (PJL), Gujarati Indian from Houston, Texas (GIH), Han Chinese in Beijing, China (CHB), Kinh in Ho Chi Minh City, Vietnam (KHV), Southern Han Chinese (CHS), Japanese in Tokyo, Japan (JPT), Chinese Dai in Xishuangbanna, China (CDX), Toscani in Italia (TSI), Utah Residents (CEPH) with Northern and Western European Ancestry (CEU), Iberian Population in Spain (IBS), British in England and Scotland (GBR), Finnish in Finland (FIN), Peruvians from Lima, Peru (PEL), Mexican Ancestry from Los Angeles USA (MXL), Colombians from Medellin, Colombia (CLM), Puerto Ricans from Puerto Rico (PUR).

Fig S2

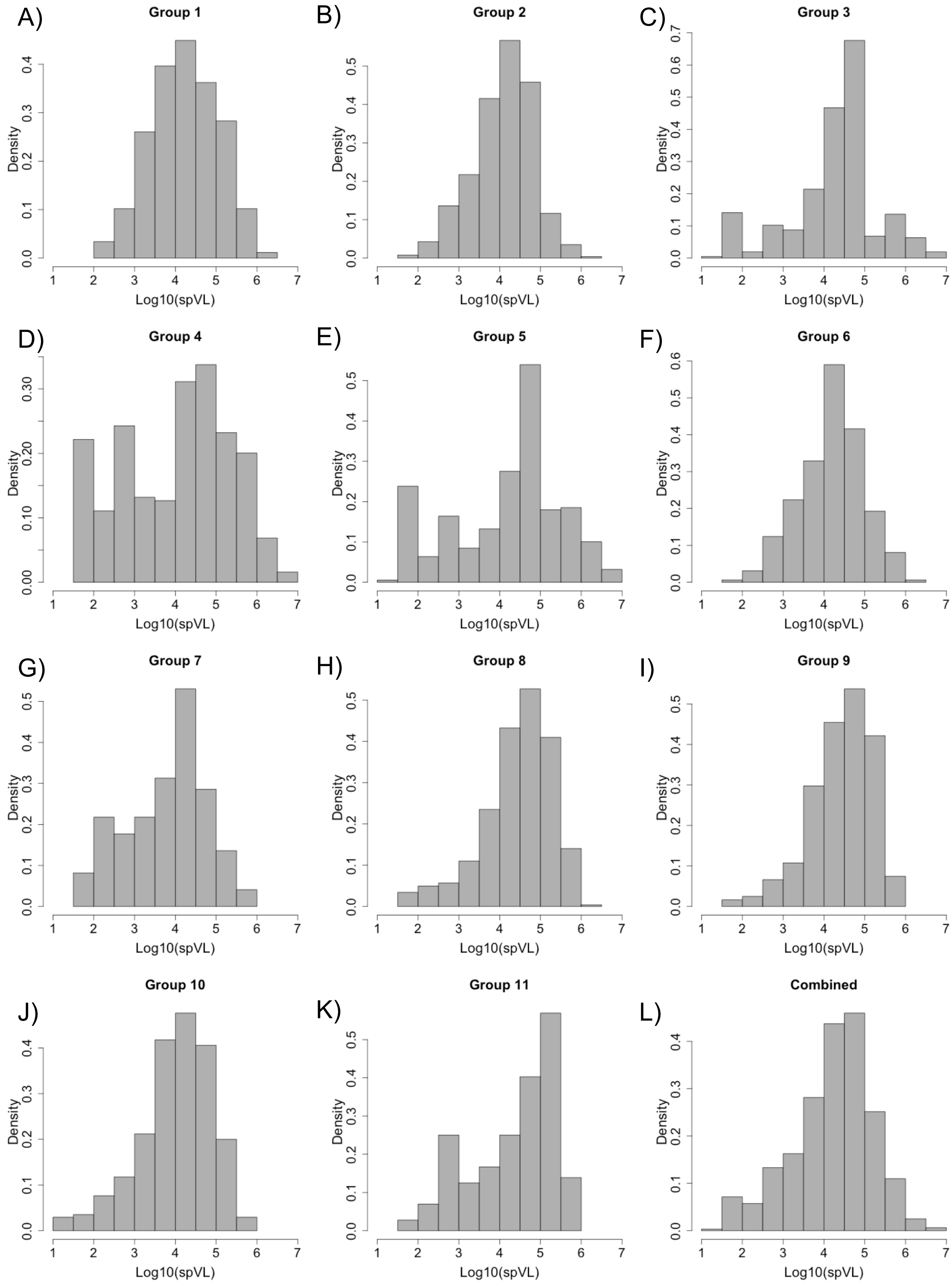
A)
Group 1
B)
Group 2
C)
Group 3
D)
Group 4
E)
Group 5
F)
Group 6
G)
Group 7
H)
Group 8
I)
Group 9
J)
Group 10
K)
Group 11
L)
Combined
Density
Log10(spVL)

Figure S2. Histograms of spVL per-group (A-K) and combined (L). Set point viral load, defined as mean $\log_{10}$(HIV-1 RNA copies/ml of plasma) during chronic infection is a reliable and established correlate of disease progression.

Fig S3

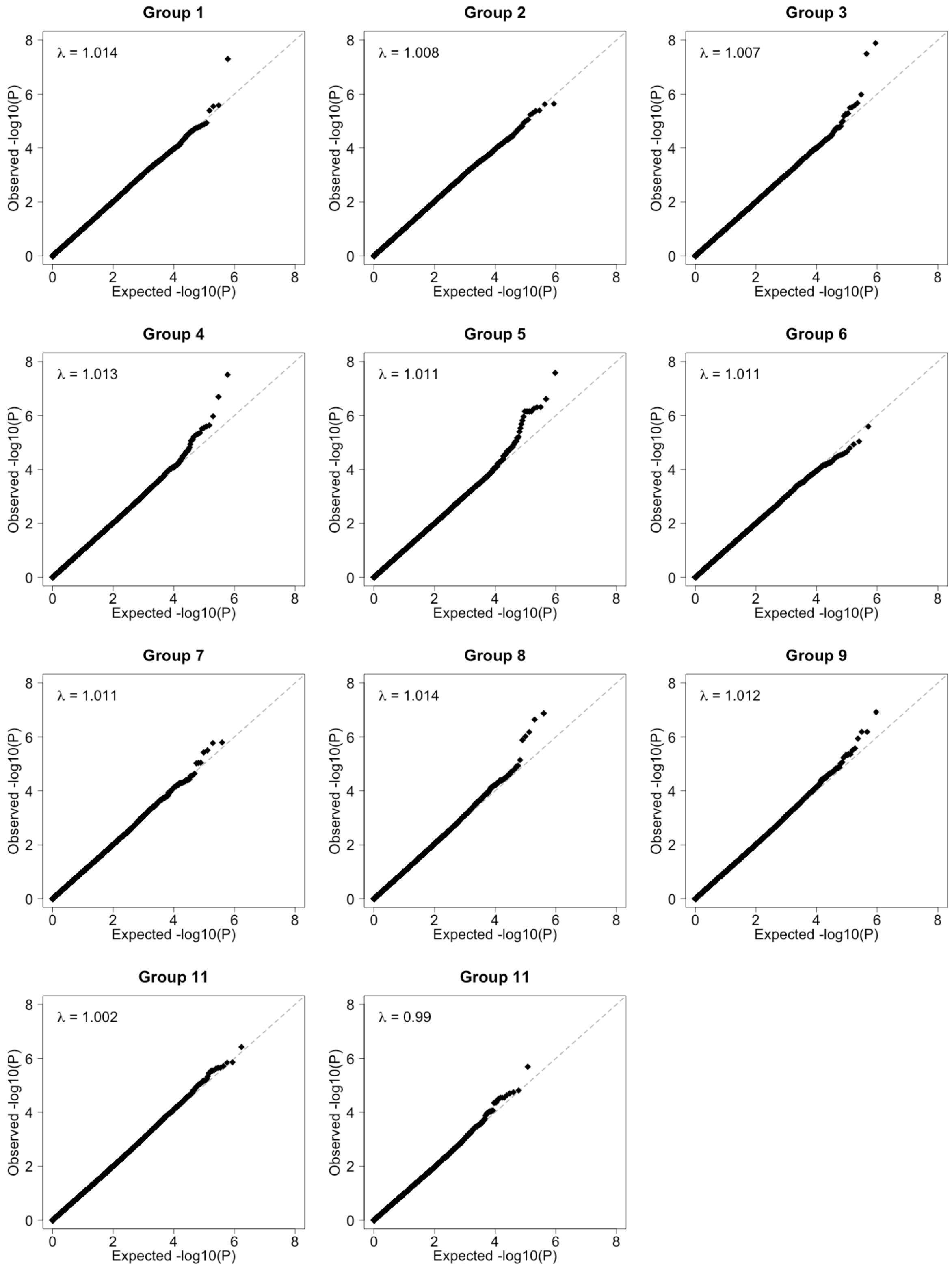
Group 1
λ = 1.014
Group 2
λ = 1.008
Group 3
λ = 1.007
Group 4
λ = 1.013
Group 5
λ = 1.011
Group 6
λ = 1.011
Group 7
λ = 1.011
Group 8
λ = 1.014
Group 9
λ = 1.012
Group 11
λ = 1.002
Group 11
λ = 0.99
Observed -log10(P)
Expected -log10(P)

Figure S3. Quantile-quantile plots of genome-wide association results per genotype group. Observed -$\log_{10}$(P) (black diamonds, y-axis) is plotted against a null expectation of no association (x-axis, dashed line). Results displayed are from association testing between spVL and directly genotyped variants per group. Association between variant dosage and spVL was tested using linear regression including principal components to correct for population structure. Principal components (PCs) were calculated per-group using EIGENSTRAT on high-quality variants pruned for LD (see methods). PCs associating with spVL ($p<0.05$) were included for each group. This method was sufficient to control genomic inflation for each group ($\lambda \sim 1$).

Fig S4

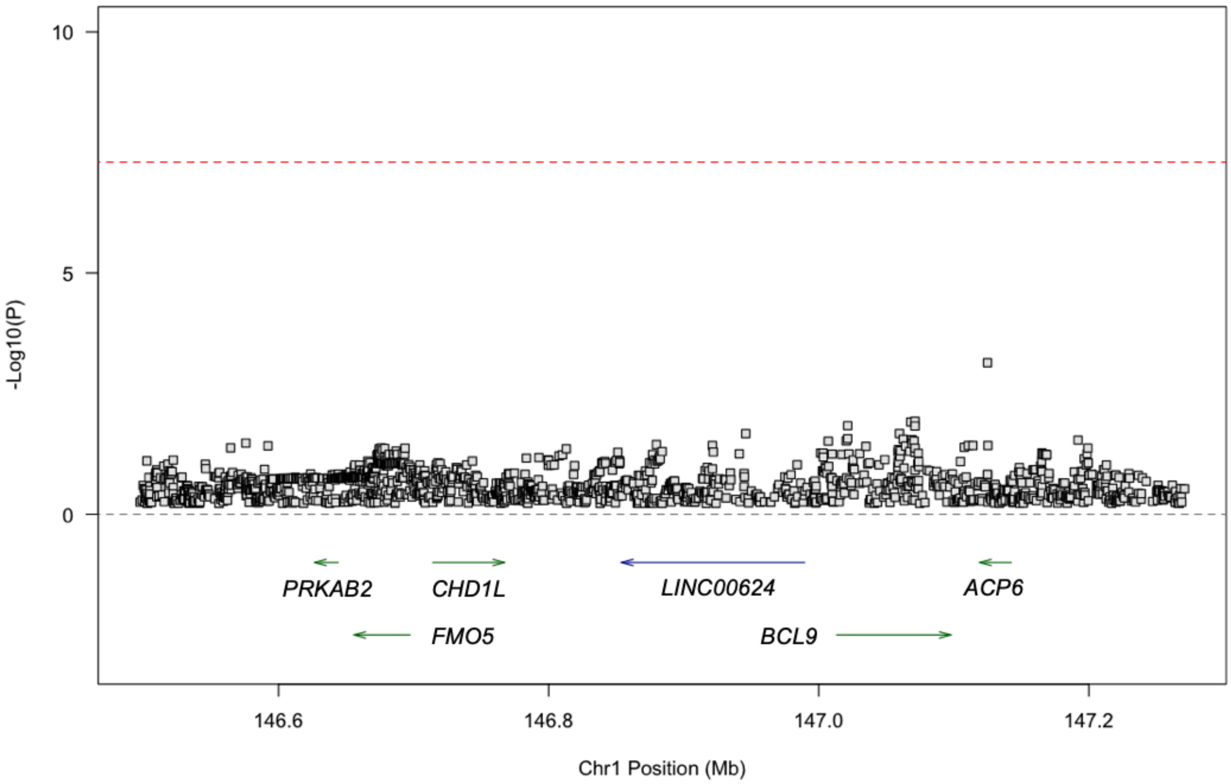

Figure S4. No association between genetic variants in the chromosome 1 region identified in the African sample and spVL in 6,315 HIV-1 infected individuals of European ancestry. *P*-values for all variants on chromosome 1 from Mb 146.5 to 147.3, corresponding to the associated region in the African sample, were extracted from our previous meta-analysis[9]. Each variant (grey box) is plotted by its genomic position (x-axis) and statistical significance ($-\log_{10}(P)$, y-axis). The red dashed line indicates the screening threshold for significance ($P<5x10^{-8}$). Arrows below the grey dashed line indicate the location and direction of transcription of protein-coding genes (green) and non-coding RNA (blue).

Fig S5

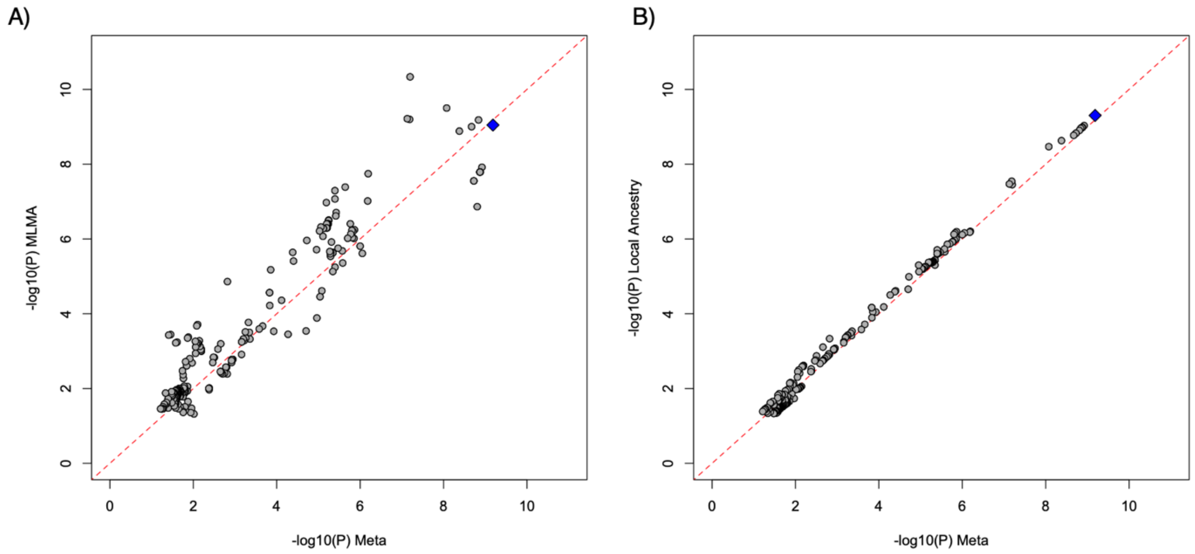

Figure S5. Correlation of p-values across the chromosome 1 variants between different models. -log10 P-values are plotted for all variants in the chromosome 1 region (Mb 146.5 – 147.1) with P<0.05 in at least 1 model. Results obtained using inverse variance weighted meta-analysis (Meta, x-axis) are highly consistent with results from linear mixed models (panel A, MLMA) and in models correcting for local ancestry (panel B, Local Ancestry). The dashed red line indicates the diagonal (i.e. a=b) and the blue diamond shows the significance of rs59784663.

Fig S6

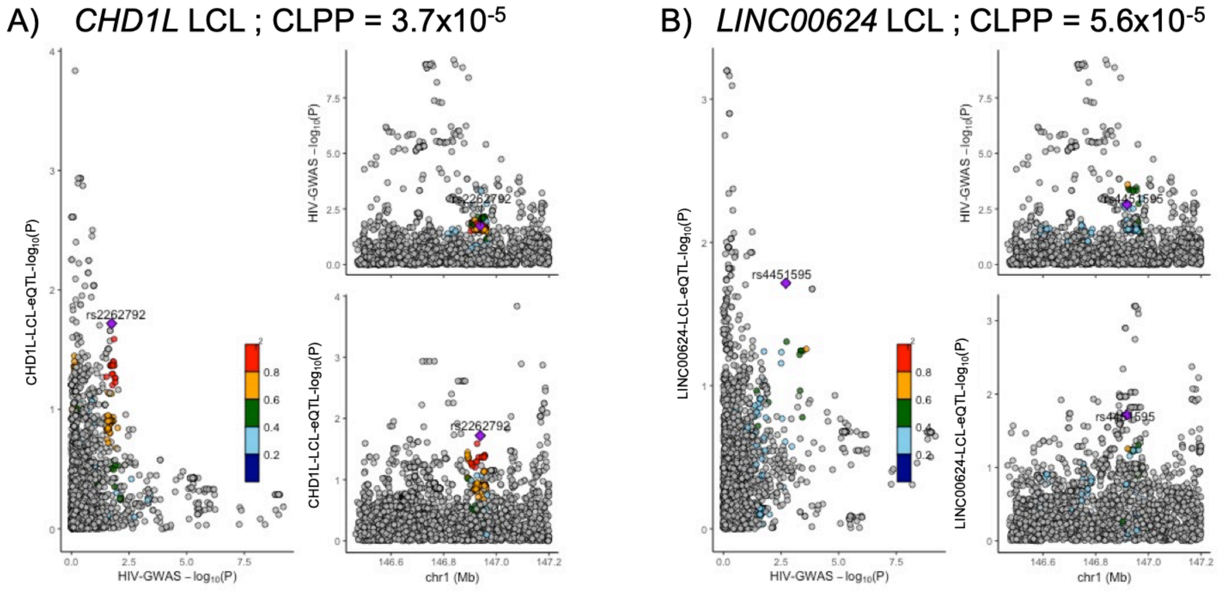

Figure S6. Colocalization plots and colocalization posterior probabilities (CLPP) testing for a relationship between variant impact on spVL and expression of *CHD1L* (A) and *LINC00624* (B) in LCLs. For each gene, the large left panel represents a scatterplot comparing GWAS -$\log_{10}$(p-value) (x-axis) from the African spVL analysis and eQTL -$\log_{10}$(p-value) (y-axis) in LCLs of the 1000Genomes AFR populations. Inset plots of -$\log_{10}$(GWAS P) and -$\log_{10}$(eQTL P) for variants in the chr1 region (position, x-axis) are shown in the top and bottom respectively. CLPP were calculated using eCAVIARv2.1. No significant colocalization was observed for the genes tested.

Fig S7

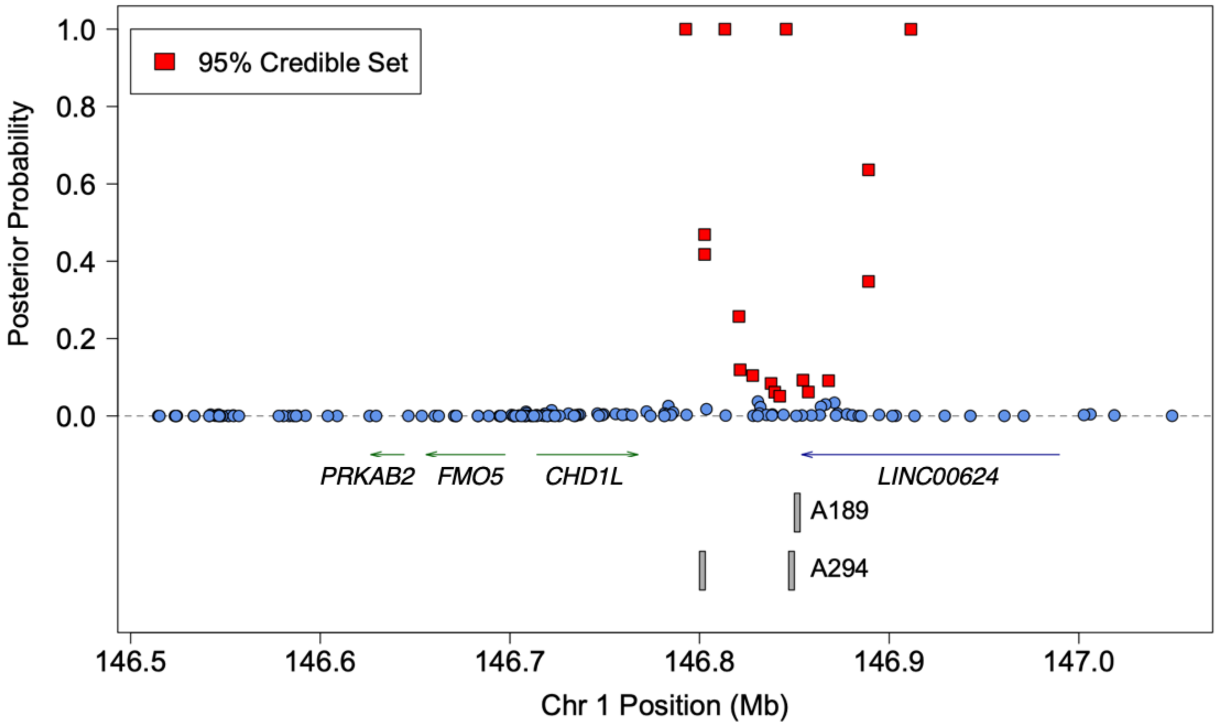

Figure S7. Scatterplot of PAINTOR posterior probabilities (y-axis) for variants located in the chromosome 1 associated region (position, x-axis) and significant functional annotations determined by likelihood ratio testing and Chi-square analysis. All variants in LD (R2 ≥ 0.2) with rs59784663 were included in the analysis. Variants making up the 95% credible set are coloured in red. Arrows indicate boundaries of protein coding genes (green) and transcribed sequences (blue). Grey rectangles indicate mapping locations of functional annotations significantly influencing causal variant prioritization; A189 – ZNF274 binding, A294 - H3K27 acetylation in naïve primary CD4+ T cells.

Fig S8

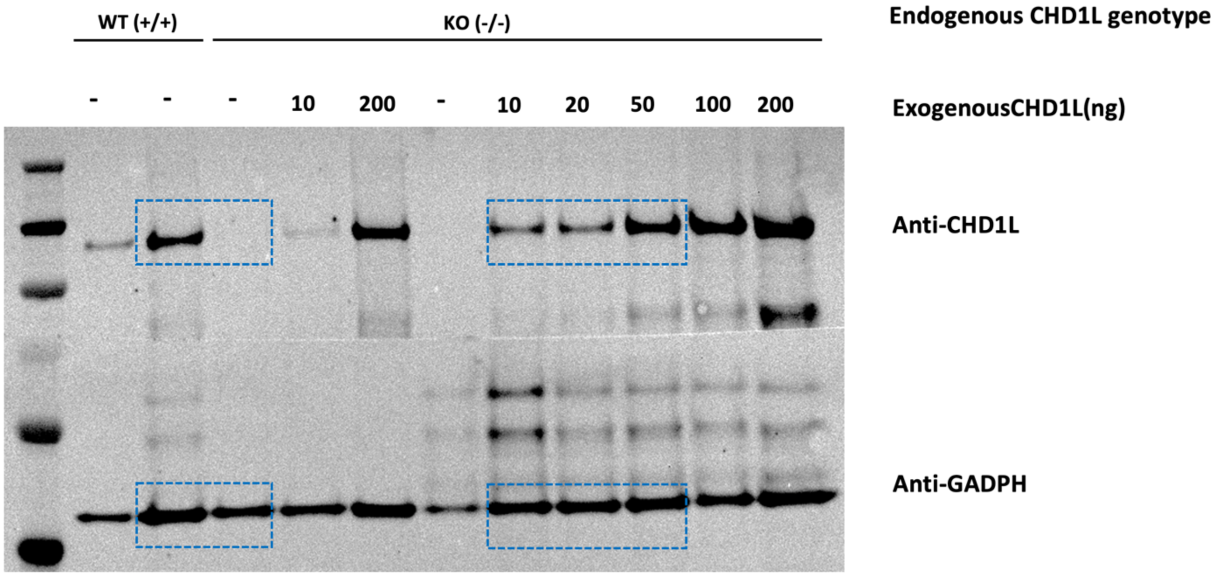

Figure S8. Expression of CHD1L in U2OS rescue experiments. Whole blot corresponding to Fig. 3 is shown. Bands cropped from this figure are outlined in blue.

Fig S9

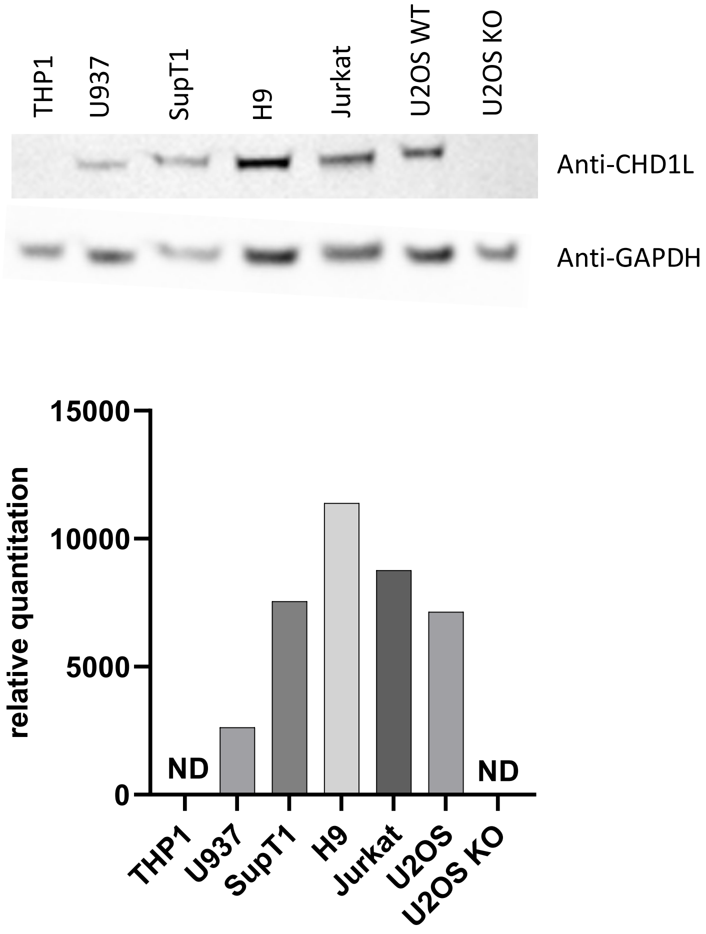

Figure S9. Detection of CHD1L in U2OS, T and myeloid cell lines. The indicated cell lines were lysed in RIPA buffer and quantity of CHD1L analysed by immunoblot relative to GAPDH using ImageJ. THP1, U937 (myeloid), SupT1 and H9 (T cell) lines were also analysed for comparison. THP1 and U937 were differentiated with PMA prior to lysis. ND = not detected. Note that CHD1L can be detected in THP1 in our hands but was below the limit of detection in this experiment.

Fig S10

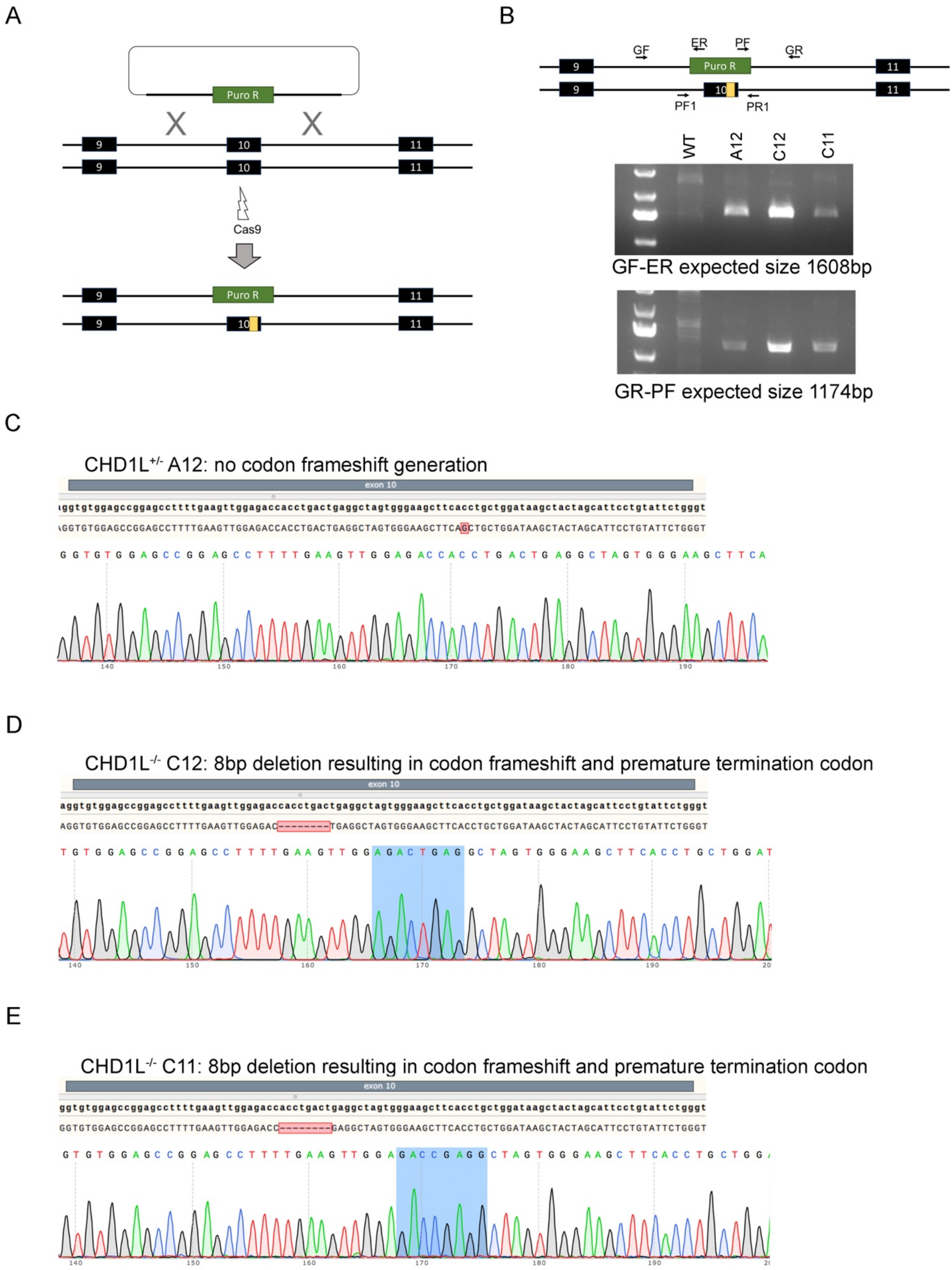
A
Puro R
9
10
11
Cas9
B
GF
ER
PF
GR
PF1
PR1
WT
A12
C12
C11
GF-ER expected size 1608bp
GR-PF expected size 1174bp
C
CHD1L+/- A12: no codon frameshift generation
exon 10
D
CHD1L-/- C12: 8bp deletion resulting in codon frameshift and premature termination codon
exon 10
E
CHD1L-/- C11: 8bp deletion resulting in codon frameshift and premature termination codon
exon 10

Figure S10. Generation of iPSC *CHD1L* mono and biallelic knockout mutants. A) Strategy for the generation of knockouts consists of replacing exon 10 of one allele with a drug selection cassette by HDR and disrupting the second allele by error-prone NHEJ. B) PCR-based validation of the puromycin cassette integration in the correct locus on the targeted allele. PCR products of indicated sizes were generated by gene specific (GF and GR) and cassette specific (ER and PF) primers for both the 5′ and 3′ end. C- E) Sanger sequencing of the non-targeted allele for A12 (C), C12 (D) and C11 (E). PCR products were generated around the site using gene specific primers (PF1 and PR1) reported in B before Sanger sequencing. Below each sequence, the relative electropherogram is reported. Mutation sites are highlighted.

Fig S11

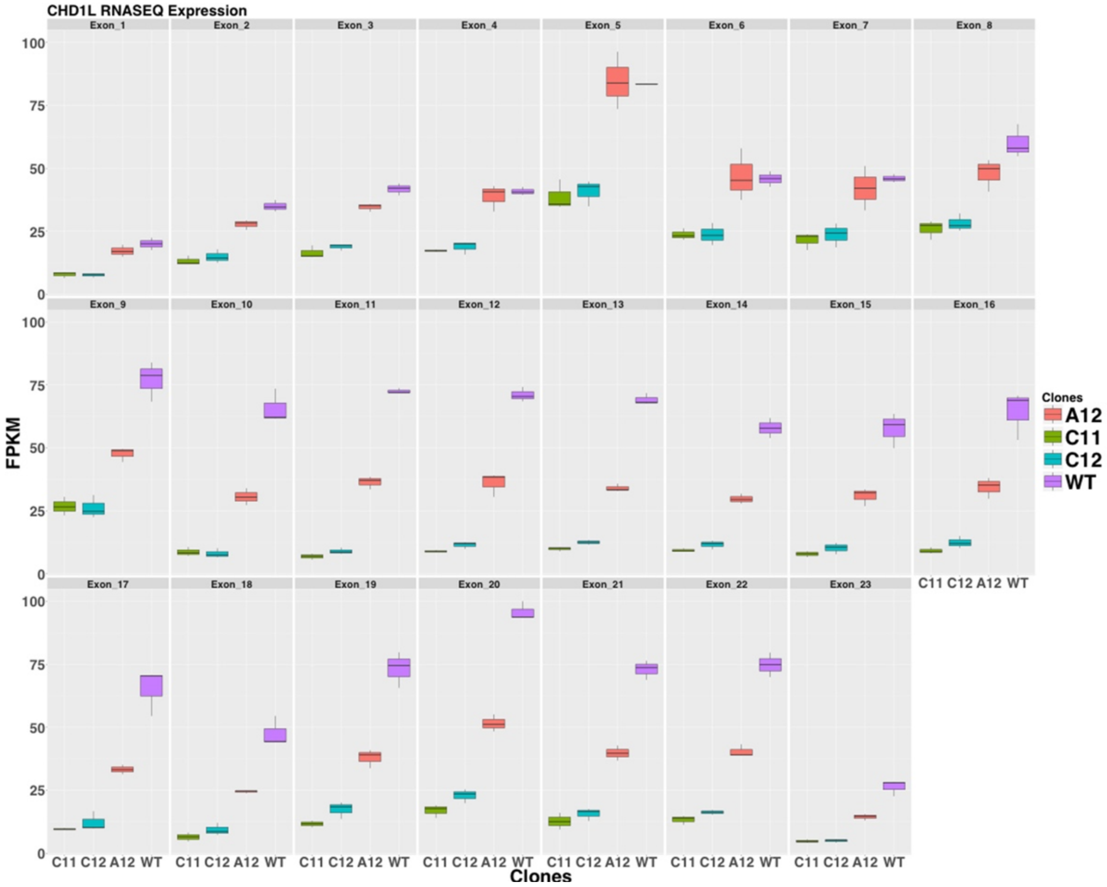

Figure S11. Boxplot representation of the FPKM (Fragments per kilobase of transcript per million) values for the full length *CHD1L* transcript for each clone. FPKM was used to normalize the gene expression data between the clones and represents the expression levels of the exons.

Fig S12

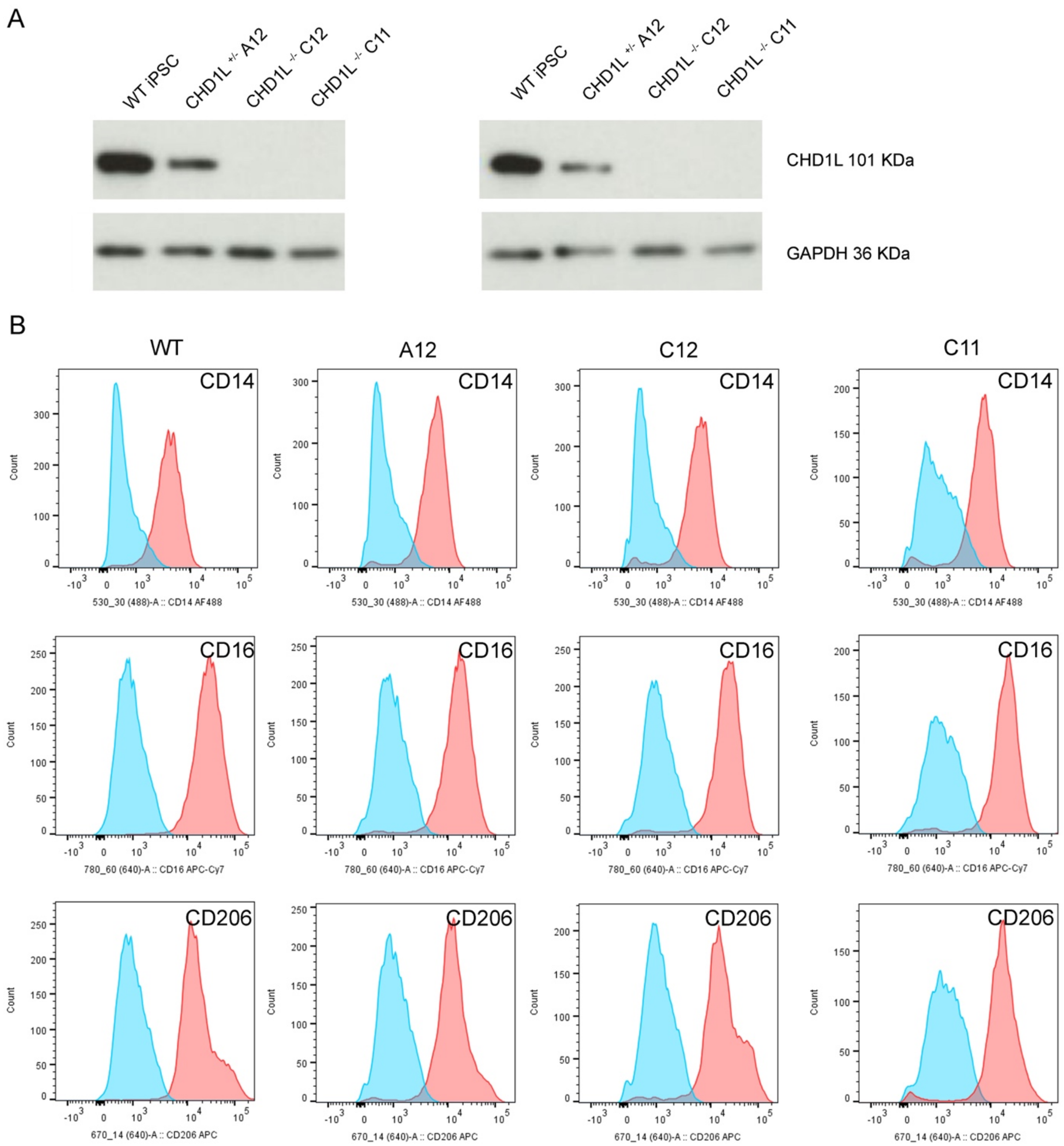

Figure S12. CHD1L knockout efficiency in iPSCs and differentiation into macrophages. (A) Western blot of CHD1L protein in A12, C12 and C11 iPSCs in comparison to WT. These represent two replicates from independent lysates and

were used for the densitometry analysis reported in Fig. 4b. Levels of GAPDH were determined as a loading control. (B) Flow cytometry analysis shows that all the three knockouts were able to differentiate into macrophages and expressed the macrophage markers CD14, CD16 and CD206. Blue and red histograms represent unstained and cells stained with relevant antibody, respectively.

Table S1

Table S1. Studies included in the genome-wide meta-analysis of host control of HIV-1 spVL

Group	N	Genotyping Platform	Country	Contributing center	Analysis stage
1	530	Illumina 550	USA (African American)	Research Triangle Institute	Discovery
2	515	Illumina 1M Duo	USA (African American)	Multicenter AIDS Cohort Study, Department of Defense Human Immunodeficiency Virus Natural History Study	
3	411	Illumina 1M Duo	USA (African American)	International HIV Controllers Study, AIDS Clinical Trials Group	
4	379	Illumina 650Y			
5	378	Illumina 1M Duo			
6	322	Affymetrix 6.0	USA (African American)	AIDS Linked to the Intravenous Experience, DC Gays, Hemophilia Growth and Development Study, Multicenter Hemophilia Cohort Study, San Francisco City Clinic	
7	147	Affymetrix 5.0	Kenya	University of Manitoba/University of Nairobi	
8	496	Illumina 1M Duo	Kenya, Rwanda, South Africa, Uganda, Zambia	International AIDS Vaccine Initiative	Replication
9	242	Illumina 1M Duo			
10	333	Illumina H3Africa array	African ancestry living in Switzerland	Swiss HIV Cohort Study	
11	126	Illumina 1M Duo	Uganda	Wellcome Trust Sanger Institute	

Table S2

Table S2. Correlation between rs1131446-T and classical class I HLA alleles and variable amino acid positions

**Gene**	**Allele**	**R^2 with rs1131446**
HLA-B	Pos 97(V)	0.8109
HLA-B	*57:03	0.6405
HLA-B	Pos 45(M)	0.5279
HLA-B	Pos 46(A)	0.5279
HLA-B	Pos 62(G)	0.3868
HLA-B	Pos 65(R)	0.3271
HLA-B	Pos 66(N)	0.3271
HLA-B	Pos 67(M)	0.3271
HLA-B	Pos 70(S)	0.3271
HLA-C	*18:01	0.1766

Pos: Position within the HLA protein. Amino acid residue carried at the given position is listed in parentheses.

Only alleles with $R^2 > 0.15$ are shown

Table S3

Table S3. Frequency of rs73001655-A in the 1000Genomes super populations

Population	Abbreviation	N	Frequency
African	AFR	661	0.068
Admixed American	AMR	347	0.006
East Asian	EAS	504	0.000
European	EUR	503	0.000
South Asian	SAS	489	0.000
Combined	ALL	2,504	0.019

Table S4

Table S4. Frequency of rs73001655-A in the 1000Genomes African sub-populations

**Population**	**Abbreviation**	**N**	**Frequency**
African Caribbean in Barbados	ACB	96	0.036
African Ancestry in Southwest US	ASW	61	0.057
Esan in Nigeria	ESN	97	0.126
Gambian in Western Division, The Gambia	GWD	112	0.075
Luhya in Webuye, Kenya	LWK	99	0.061
Mende in Sierra Leone	MSL	85	0.065
Yoruba in Ibadan, Nigeria	YRI	108	0.051

Table S5

Table S5. Frequency of rs59784663-G in the 1000Genomes African sub-populations

Population	Abbreviation	N	Frequency
African Caribbean in Barbados	ACB	96	0.036
African Ancestry in Southwest US	ASW	61	0.057
Esan in Nigeria	ESN	97	0.121
Gambian in Western Division, The Gambia	GWD	112	0.075
Luhya in Webuye, Kenya	LWK	99	0.056
Mende in Sierra Leone	MSL	85	0.059
Yoruba in Ibadan, Nigeria	YRI	108	0.051

Table S6

Table S6. Frequencies of genome-wide significant chromosome 1 variants in the 1000 Genomes super-populations

Variant	Alt	Ref	AFR	EUR	AMR	EAS	SAS
rs59784663	G	A	0.065	0	0.006	0	0
rs72999655	G	A	0.065	0	0.006	0	0
rs72999634	G	A	0.065	0	0.006	0	0
rs7526114	A	G	0.065	0	0.006	0	0
rs72999646	A	T	0.065	0	0.006	0	0
rs72999637	C	T	0.065	0	0.006	0	0
rs72999648	A	G	0.065	0	0.006	0	0
rs7535451	G	A	0.065	0	0.006	0	0
rs59213667	A	G	0.065	0	0.007	0	0
rs73001655	A	G	0.068	0	0.006	0	0
rs72999638	A	T	0.065	0	0.006	0	0
rs72999656	T	C	0.064	0	0.006	0	0
rs77029719	G	C	0.068	0	0.006	0	0
rs72999639	C	T	0.065	0	0.006	0	0
rs72999640	T	C	0.065	0	0.006	0	0
rs73004025	T	C	0.065	0	0.006	0	0

Alt: Alternate allele

Ref: Reference allele

AFR African; EUR European; AMR Admixed American; EAS East Asian; SAS South Asian. Frequencies are reported for the Alt allele

Table S7

Table S7. Association statistics for rs59784663-G from sensitivity analyses using linear mixes models and accounting for inferred local ancestry

**Model**	**β**	**SE**	**P**
Linear regression and meta-analysis	-0.304	0.049	$6.4x10^{-10}$
Linear mixed models (GCTA)	-0.335	0.055	$9.0x10^{-10}$
Linear regression stratified by local ancestry (BMIX)	-0.338	0.053	$2.3x10^{-10}$

Table S8

<u>**Table S8**. Comparison of association results for variants within the chromosome 1 associated region (Mb146.4 – 147.4) between HIV-1 spVL and haematological traits</u>

**Trait**	**Unit**	**Sample no.**	**rs59784663(*P*)**	**Top Var**	**Top Var Trait(*P*)**	**Top Var spVL(*P*)**
WBC	x10^9/l	1625	2.7E-1	rs114116212	2.3E-3	9.0E-1
RBC	x10^9/l	1625	7.8E-1	rs78725746	4.6E-4	8.3E-1
MCV	fl	1625	9.7E-1	rs35298140	5.4E-4	3.2E-1
MCH	pg/cell	1625	2.5E-1	rs35298140	8.1E-4	3.2E-1
MCHC	g/L	1625	1.3E-2	rs75556492	1.9E-3	3.7E-1
RDW	%	1625	3.1E-1	rs17160779	1.0E-4	8.4E-1
Haemoglobin	g/L	1625	8.2E-1	rs4950461	9.6E-4	9.9E-1
PLT	x10^9/l	1625	8.9E-1	rs143937319	1.7E-3	3.9E-2
Lymphocytes	x10^9/l	1565	9.9E-1	rs80180615	5.03E-4	1.9E-01
Monocytes	x10^9/l	1565	4.5E-1	rs61812508	7.8E-4	1.6E-2
Basophils	x10^9/l	1565	6.4E-1	rs74550663	8.1E-4	8.9E-1
Neutrophils	x10^9/l	1555	2.4E-1	rs115559671	4.5E-4	3.0E-2
Eosinophils	x10^9/l	1555	6.6E-1	rs12755420	3.5E-4	6.2E-1

WBC, White blood cell count; RBC, Red blood cell count; MCV, mean corpuscular volume; MCH, mean corpuscular haemoglobin; MCHC, mean corpuscular haemoglobin concentration; RDW, red blood cell distribution width; PLT, platelet count.

rs59784663(*P*): Association *P*-value for rs59784663 and the listed trait

Top Var: The variant within the chr1 Mb146.4 – 147.4 region with the lowest haematological trait-associated *P*-value

Top Var Trait(*P*): Association *P*-value for the top variant and the listed haematological trait

Top Var spVL(*P*): Association *P* -value for the top haematological trait associated variant and HIV-1 spVL

Table S9

Table S9. Populations and sample sets included in the eQTL analysis. For each population the number variants tested in the Chromosome 1 region is listed

**Country**	**Ethnolinguistic group**	**Count**	**Variants**	**Genotype Source**	**RNA Expression Source**	**RNA Expression Location**
Nigeria	Esan (ESN), Volta-Niger	99	5,108	1000G	WTSI	WTSI
Gambia	Mandinka (GWD) Mande	112	5,745	1000G	WTSI	WTSI
Kenya	Luhya (LWK), Bantu	97	6,018	1000G	WTSI	WTSI
Kenya	Maasai (MKK), Kinyawa	164	6,975	WTSI	WTSI	WTSI
Sierra Leone	Mende(MSL) Mande	83	5,431	1000G	WTSI	WTSI
Nigeria	Yoruba(YRI) Volta-Niger	41	4,869	1000G	WTSI	WTSI

WTSI: Wellcome Trust Sanger Institute

Table S10

Table S10. Variants making up the 95% credible set identified using PAINTOR

Variant ID	ALT	REF	P value	β	SE	Posterior probability	R^2 with rs59784663
rs7520841	C	T	7.65E-05	0.108	0.027	0.99998	0.348
rs2353984	T	C	1.39E-04	-0.104	0.027	0.99998	0.346
rs72694728	A	T	2.28E-06	0.169	0.036	0.99998	0.672
rs6675942	A	G	5.74E-03	-0.070	0.025	0.99975	0.272
rs59987487	A	C	1.48E-04	0.129	0.034	0.63633	0.527
rs66486822	A	T	5.03E-06	0.138	0.030	0.46897	0.481
rs72692962	A	C	5.34E-06	0.137	0.030	0.41762	0.481
rs7417503	G	C	1.49E-04	0.129	0.034	0.34761	0.523
rs11239997	A	G	1.21E-03	-0.081	0.025	0.25712	0.257
rs4314933	C	T	1.12E-03	-0.082	0.025	0.11908	0.255
rs10900350	G	T	1.19E-03	-0.081	0.025	0.10437	0.255
rs4950423	C	T	1.97E-03	-0.077	0.025	0.09238	0.252
rs10793682	G	T	2.20E-03	-0.077	0.025	0.09112	0.253
rs2353975	A	C	1.18E-03	-0.081	0.025	0.08402	0.254
rs4295925	C	T	6.98E-04	-0.083	0.024	0.06187	0.238

rs2353977	T	G	1.66E-03	-0.079	0.025	0.06143	0.253
rs7534705	C	T	1.54E-03	-0.079	0.025	0.05099	0.251

P value, β and standard error are results of linear regression and meta-analysis testing for association between variant dosage and HIV spVL

Posterior probabilities are likelihoods of variants being in the causal set as calculated by PAINTOR

Table S11

Table S11. PCR primers and CRISPR sequences utilized in generation and genotyping of *CHD1L*$^{-/-}$ Jurkat E6.1 mutant clones.

**CRISPR target sites (PAM site underlined)**	
G2 CRISPR	CCTGCTGGATAAGCTACTAGCAT
G4 CRISPR	AGTTGGAGACCACCTGACTGAGG
**Gene specific PCR primers for amplification of the target locus**	
CHD1L_2F	CACAGATGTTTATTAGGCTTGTTGGATGTGC
CHD1L_2R	GTACAAATTCAGGAAACGAAAGAATGATTGTGGAAG
**Gene-specific PCR primer for Sanger sequencing**	
CHD1L_F1	GGCTGTGTAATTGGTGTTAAGCAGGAC

Table S12

Table S12. PCR primers and CRISPR sequences utilized in generation and genotyping of CHD1L$^{-/-}$ iPSC mutant clones

**CRISPR target sites (PAM site underlined)**	
Left CRISPR	CCACCTGACTGAGGCTAGTGGGA
Right CRISPR	CTACTAGCATTCCTGTATTCTGG
**Gibson primers for CHD1L exon 10 5' and 3' arms amplification (append sequence underlined)**	
CHD1L_5F	AACGACGGCCAGTGAATTCGATTATTTGTTTGATGGTGAGACAGTGC
CHD1L_5R	TATCGTTATGCGCCTTGATTCTCAGTCCTGCTTAACACCAATTAC
CHD1L_3F	CTGAGCTAGCCATCAGTGATTTCTTAACACTGATTCAGGTTCTGCC
CHD1L_3R	CCATGATTACGCCAAGCTTGATATGGCAGATATACGAGAACCAATG
**Universal sequencing primers, Intermediate vector**	
p19F	AACTGTTGGGAAGGGCGATC
ZP1	GAAGTCGTCCTCCACGAAGT
ZP2	GAACTGTGGTTACGCGAATG
p19R	GTTAGCTCACTCATTAGGCAC
**Universal sequencing primers, Final vector**	
p19F	AACTGTTGGGAAGGGCGATC
EF1aR1	CTCTGGGTTCTACGTTAGTG

pA_R	CGCGTCGAGAAGTTCCTATTC
p19R	GTTAGCTCACTCATTAGGCAC
**Cassette PCR primers for genotyping targeted allele**	
ER	CTCTGGGTTCTACGTTAGTG
PF	ATCCGGGGGTACCGCGTCGAG
**Gene specific PCR primers for genotyping targeted allele**	
CHD1L_GF	GGGTTTTGGTTTCTGTTTTGTTCAG
CHD1L_GR	AGAAGGCATTTATAGGTCCTTCTCC
**Gene-specific PCR primers for genotyping non-target allele**	
CHD1L_PR1	AAGAGTCCTTCAAGTTCCCCTTAAG
CHD1L_PF1	CTCTGCCAAATAGTGCTAAGAACTG
**Gene-specific sequencing primers for genotyping non-targeted allele**	
CHD1L_SR1	AGCAGATTACGGCAGCACAT
CHD1L_SF1	GCTTGTTGGATGTGCTAGGC

Supplementary Note 1: Characterization of Jurkat CHD1L-/- clones and infection assays

To understand the role of *CHD1L* in other cellular models relevant to HIV biology, we generated *CHD1L* knockouts in Jurkat E6.1 cells (ATCC, TIB-152), a human immortalized T-lymphocytic cell line commonly used in HIV research. The *CHD1L* gene was disrupted by the CRISPR/Cas9 technology, using two gRNAs targeting the exon 10 (G2 and G4 CRISPR in table S12 leading to frameshift indels and premature stop codons. Sanger sequencing was used to identify one heterozygote knockout clone (one WT and one mutated allele, generated with G2 CRISPR) and 4 homozygous clones (with frameshift indels on both alleles, one generated with G4 CRISPR and the others with G2 CRISPR). Western blot verified the loss of functional CHD1L protein in the knockout clones (Extended Data Fig. 3a). CHD1L was undetectable in all homozygous knockout clones while its level was reduced in the heterozygote.

CHD1L knockout clones were infected with different concentrations of NL4-3-deltaEnv-GFP/VSV-G virus (0-300 ng of p24) and the percentage of infected cells (GFP-positive) monitored at 48 hours post-infection. No differences where observed between WT and *CHD1L*-depleted cells in the infection rate, nor in viability (Extended Data Fig. 3b,c). To rule out a possible time-related effect, we monitored the

percentage of infected cells at different time points (24, 36 and 48 hours) post-infection with 300 ng of p24 NL4-3-deltaEnv-GFP/VSV-G virus. However, no differences were observed between WT and *CHD1L* knockout clones at the analysed time points (Extended Data Fig. 3d). These findings were confirmed in Jurkat knock-down experiments using CHD1L specific shRNAs (SMARTvector Inducible Lentiviral shRNA - Dharmacon) (data not shown). HIV replication assays in this T-cell line consistently show the lack of a viral restriction phenotype associated to CHD1L suggesting that it is not active in this cellular context. This could be explained by multiple hypotheses: i) the observed *CHD1L* knockout HIV phenotype is macrophage-specific ii) the genetic background and eventual genomic alterations of the immortalized cell line could diminish the effect of *CHD1L* on HIV infection. Indeed, the Jurkat cell line was isolated from the blood of a patient with Acute Lymphoblastic Leukemia and as such harbors many mutations in cancer-associated genes involved in the maintenance of genome stability and also in immunity-related genes important for T-lymphocyte function[63].

Supplementary Note 2: Cohorts and Individuals contributing to the International Consortium for the Genomics of HIV

The AIDS Clinical Trials Group (ACTG)

DESCRIPTION:

The AIDS Clinical Trials Group (ACTG) was initially established in 1987 to broaden the scope of the AIDS research effort of the US National Institute of Allergy and Infectious Diseases (NIAID). The ACTG established and supports the largest Network of expert clinical and translational investigators and therapeutic clinical trials units in the world, including sites in resource-limited countries. These investigators and units serve as the major resource for HIV/AIDS research, treatment, care, and training/education in their communities.

MEMBERS:

Eric S. Daar, Roy M. Gulick, David W. Haas, Richard Haubrich, Daniel R. Kuritzkes, Heather J. Ribaudo, Sharon Riddler, Gregory K. Robbins, Paul E. Sax, Robert W. Shafer, Cecilia M. Shikuma

FUNDING:

Research reported in this publication was supported by the National Institute of Allergy and Infectious Diseases of the National Institutes of Health under Award Number UM1 AI068634, UM1 AI068636 and UM1 AI106701. The content is solely the responsibility of the authors and does not necessarily represent the official views of the National Institutes of Health. Grants to Clinical Research Sites that participated in ACTG protocols and collected DNA under protocol A5128 included: AI069477, AI027675, AI073961, AI069474, AI069432, AI069513, AI069423, AI050410, AI069452, AI69450, AI054907, AI069428, AI045008, AI069495, AI069415, AI069556, AI069484, AI069424, AI069532, AI069419, AI069471, AI025859, AI069418, AI050409, AI069501, AI069502, AI069511, AI069434, AI069465, AI069494, AI069472, AI069470, AI046376, AI072626, AI027661, AI034853, AI069447, AI032782, AI027658, AI27666, AI058740, AI046370, TR002243, RR00051, RR00046, RR025747, RR025777, RR024160, RR024996, and RR024156. Abbott Laboratories, Boehringer-Ingelheim, Bristol-Myers Squibb, Gilead Sciences, and GlaxoSmithKline provided study medications. DWH is also supported by NIH grants AI077505 and AI110527.

Ethics:

This study was approved by the Institutional Review Board at Vanderbilt University, Nashville USA.

The AIDS Linked to the IntraVenous Experience (ALIVE) Cohort

DESCRIPTION:

The AIDS Linked to the IntraVenous Experience (ALIVE) study is a natural history study following the incidence and progression of HIV infection among intravenous drug users (IDUs) in Baltimore, MD.

MEMBERS:

Gregory D. Kirk, Shruti H. Mehta, Kenrad Nelson, Steffanie Strathdee, David Vlahov

FUNDING:

National Institutes of Health grants: R01-DA-04334 and R01-DA-12568

Ethics:

This study was approved by the Institutional Review Board of the Johns Hopkins School of Medicine, Baltimore USA.

The Center for Cancer Research, National Cancer Institute

DESCRIPTION:

The Center for Cancer Research (CCR) is home to more than 250 scientists and clinicians working in intramural research at NCI. CCR's investigators are basic, clinical, and translational scientists who work together to advance knowledge of cancer and AIDS and to develop new therapies against these diseases.

MEMBERS:

Ping An, Sher Hendrickson, Randall Johnson, Bailey Kessing, James Lautenberger, Carl McIntosh, George Nelson, Stephen O'Brien, Efe Szegin, Jennifer Troyer, and Cheryl Winkler

FUNDING:

This research was supported by the Intramural Research Program of NIH, Frederick National Laboratory, Center for Cancer Research, National Cancer Institute and also funded in whole or in part with Federal funds from the Frederick National Laboratory for Cancer Research, National Institutes of Health, under contract HHSN261200800001E.

Ethics:

Genetic studies of this cohorts has been granted by the NIH Office of Human Subjects Research.

The Center for HIV/AIDS Vaccine Immunology (CHAVI)

DESCRIPTION:

The Center for HIV/AIDS Vaccine Immunology (CHAVI) is a consortium of universities and academic medical centers that was established by the National Institute of Allergy and Infectious Diseases (NIAID) from 2005-2012. CHAVI's goal was to solve the major problems in HIV vaccine development and design.

MEMBERS:

CHAVI is led by Barton Haynes (Duke University, Durham, NC, USA). Its Host Genetics Core is led by David Goldstein (Duke University, Durham, NC, USA).

FUNDING:

Funding for research by CHAVI was provided by NIH NIAID grant AI067854

Ethics:

All participating centers provided local institutional review board approval

The Hemophilia Growth and Development Study (HGDS)

DESCRIPTION:

The HGDS is a multicenter study of hemophilia and its complications that was established in 1988. Data were prospectively collected in 14 U.S. hemophilia treatment centers through 1996/97. A total of 333 children and adolescents were enrolled. The HGDS has investigated the effects of hemophilia and HIV on physical growth and maturation; immunological, neurological, and neuropsychological functioning; and the pathophysiology of HIV and hepatitis C.

MEMBERS:

Eric Daar, Sharyne Donfield, Edward Gomperts, Margaret Hilgartner, W. Keith Hoots, Henry Lynn, Anne Willoughby, Cheryl Winkler

FUNDING:

National Institutes of Health, National Institute of Child Health and Human Development, R01-HD-41224

Ethics:

Genetic studies of this cohorts has been granted by the NIH Office of Human Subjects Research.

The International HIV Controllers Study

DESCRIPTION:

The International HIV Controllers Study is a collaborative effort among scientists, healthcare professionals and the community to study HIV infected people who have been able to maintain low viral loads without the use of medications.

MEMBERS:

HIV controllers recruitment and sample management: Florencia Pereyra, Alicja Piechocka-Trocha, Emily Cutrell, Rachel Rosenberg, Kristin L. Moss, Ildiko Toth, Brian Block, Brett Baker, Alissa Rothchild, Jeffrey Lian, Jacqueline Proudfoot, Marylyn M. Addo, Bruce D. Walker

HIV controllers referral team: Brian Agan, Shanu Agarwal, Richard L. Ahern, Brady L. Allen, Sherly Altidor, Eric L. Altschuler, Sujata Ambardar, Kathryn Anastos, Val Anderson, Ushan Andrady, Diana Antoniskis, David Bangsberg, Daniel Barbaro, William Barrie, J. Bartczak, Simon Barton, Patricia Basden, Nesli Basgoz, Nicholaos C.

Bellos, Judith Berger, Nicole F. Bernard, Annette M. Bernard, Stanley J. Bodner, Robert K. Bolan, Emilie T. Boudreaux, James F. Braun, Jon E. Brndjar, J. Brown, Sheldon T. Brown, Jedidiah Burack, Larry M. Bush, Virginia Cafaro, John Campbell, Robert H. Carlson, J. Kevin Carmichael, Kathleen K. Casey, Chris Cavacuiti, Gregory Celestin, Steven T. Chambers, Nancy Chez, Lisa M. Chirch, Paul J. Cimoch, Daniel Cohen, Lillian E. Cohn, Brian Conway, David A. Cooper, Brian Cornelson, David T. Cox, Michael V. Cristofano, George Cuchural Jr., Julie L. Czartoski, Joseph M. Dahman, Jennifer S. Daly, Benjamin T. Davis, Kristine Davis, Sheila M. Davod, Steven G. Deeks, Edwin DeJesus, Craig A. Dietz, Eleanor Dunham, Michael E. Dunn, Todd B. Ellerin, Joseph J. Eron, John J.W. Fangman, Helen Ferlazzo, Sarah Fidler, Anita Fleenor-Ford, Renee Frankel, Kenneth A. Freedberg, Neel K. French, Jonathan D. Fuchs, Jon D. Fuller, Jonna Gaberman, Joel E. Gallant, Rajesh T. Gandhi, Efrain Garcia, Donald Garmon, Joseph C. Gathe Jr, Cyril R. Gaultier, Wondwoosen Gebre, Frank D. Gilman, Ian Gilson, Paul A. Goepfert, Michael S. Gottlieb, Claudia Goulston, Richard K. Groger, T. Douglas Gurley, Stuart Haber, Robin Hardwicke, W. David Hardy, P. Richard Harrigan, Trevor N. Hawkins, Sonya Heath, Frederick M. Hecht, W. Keith Henry, Melissa Hladek, Robert P. Hoffman, James M. Horton, Ricky K. Hsu, Gregory D. Huhn, Peter Hunt, Mark L. Illeman, Hans Jaeger, Robert M. Jellinger, Mina John, Jennifer A. Johnson, Kristin L. Johnson, Heather Johnson, Kay Johnson, Jennifer Joly, Wilbert C. Jordan, Carol A. Kauffman, Homayoon Khanlou, Arthur Y.

Kim, David D. Kim, Clifford A. Kinder, Laura Kogelman, Erna Milunka Kojic, Neeltje A. Kootstra, P. Todd Korthuis, Wayne Kurisu, Douglas S. Kwon, Melissa LaMar, Harry Lampiris, Michael M. Lederman, David M. Lee, Marah J. Lee, Edward T.Y. Lee, Janice Lemoine, Jay A. Levy, Josep M. Llibre, Michael A. Liguori, Susan J. Little, Anne Y. Liu, Alvaro J. Lopez, Mono R. Loutfy, Dawn Loy, Debbie Y. Mohammed, Alan Man, Michael K. Mansour, Vincent C. Marconi, Martin Markowitz, Jeffrey N. Martin, Harold L. Martin Jr., Kenneth Hugh Mayer, M. Juliana McElrath, Theresa A. McGhee, Barbara H. McGovern, Katherine McGowan, Dawn McIntyre, Gavin X. McLeod, Prema Menezes, Greg Mesa, Craig E. Metroka, Dirk Meyer-Olson, Andy O. Miller, Kate Montgomery, Karam C. Mounzer, Iris Nagin, Ronald G. Nahass, Craig Nielsen, David L. Norene, David H. O'Connor, Jason Okulicz, Edward C. Oldfield III, Susan A. Olender, Mario Ostrowski, William F. Owen Jr., Jeffrey Parsonnet, Andrew M. Pavlatos, Aaron M. Perlmutter, Jonathan M. Pincus, Leandro Pisani, Lawrence Jay Price, Laurie Proia, Richard C. Prokesch, Heather Calderon Pujet, Moti Ramgopal, Michael Rausch, J. Ravishankar, Frank S. Rhame, Constance Shamuyarira Richards, Douglas D. Richman, Gregory K. Robbins, Berta Rodes, Milagros Rodriguez, Richard C. Rose III, Eric S. Rosenberg, Daniel Rosenthal, Polly E. Ross, David S. Rubin, Elease Rumbaugh, Luis Saenz, Michelle R. Salvaggio, William C. Sanchez, Veeraf M. Sanjana, Steven Santiago, Wolfgang Schmidt, Philip M. Sestak, Peter Shalit, William Shay, Vivian N. Shirvani, Vanessa I. Silebi, James M. Sizemore Jr., Paul R. Skolnik, Marcia

Sokol-Anderson, James M. Sosman, Paul Stabile, Jack T. Stapleton, Francine Stein, Hans-Jurgen Stellbrink, F. Lisa Sterman, Valerie E. Stone, David R. Stone, Giuseppe Tambussi, Randy A. Taplitz, Ellen M. Tedaldi, Amalio Telenti, Richard Torres, Lorraine Tosiello, Cecile Tremblay, Marc A. Tribble, Phuong D. Trinh, Anthony Vaccaro, Emilia Valadas, Thanes J. Vanig, Isabel Vecino, Wenoah Veikley, Barbara H. Wade, Charles Walworth, Chingchai Wanidworanun, Douglas J. Ward, Robert D. Weber, Duncan Webster, Steve Weis, David A. Wheeler, David J. White, Ed Wilkins, Alan Winston, Clifford G. Wlodaver, David P. Wright, Otto O. Yang, David L. Yurdin, Brandon W. Zabukovic, Kimon C. Zachary, Beth Zeeman, Meng Zhao

FUNDING:

The International HIV Controllers Study was made possible through a generous donation from the Mark and Lisa Schwartz Foundation and a subsequent award from the Collaboration for AIDS Vaccine Discovery (CAVD) of the Bill and Melinda Gates Foundation. This work was also supported in part by the Harvard University Center for AIDS Research (P-30- AI060354), UCSF CFAR (P-30 AI27763), UCSF CTSI (UL1 RR024131), CNICS (R24 AI067039), NIH grants AI28568, AI030914 (B.D.W.); AI087145, K24AI069994 (S.G.D.)

Ethics:

HIV controllers were recruited through local outpatient clinics affiliated with the Ragon Institute of MGH, MIT and Harvard and collaborations with 335 health care providers and scientists across the US, Canada, Western Europe and Australia. The respective institutional review boards approved the study, and all subjects gave written informed consent.

The Multicenter AIDS Cohort Study (MACS)

DESCRIPTION:

The Multicenter AIDS Cohort Study (MACS) is an ongoing prospective study of the natural and treated histories of HIV-1 infection in homosexual and bisexual men conducted by sites located in Baltimore, Chicago, Pittsburgh and Los Angeles. A total of 6,972 men have been enrolled.

MEMBERS:

Baltimore: The Johns Hopkins University Bloomberg School of Public Health: Joseph B. Margolick (Principal Investigator), Barbara Crain, Adrian Dobs, Homayoon Farzadegan, Joel Gallant, Lisette Johnson, Shenghan Lai, Ned Sacktor, Ola Selnes, James Shepherd, Chloe Thio.

Chicago: Howard Brown Health Center, Feinberg School of Medicine, Northwestern University, and Cook County Bureau of Health Services: John P. Phair and Steven Wolinsky (Multiple Principal Investigators), Sheila Badri, Bruce Cohen, Craig Conover, Maurice O'Gorman, David Ostrow, Frank Palella.

Los Angeles: University of California, UCLA Schools of Public Health and Medicine: Roger Detels (Principal Investigator), Barbara R. Visscher (Co-Principal Investigator), Aaron Aronow, Robert Bolan, Elizabeth Breen, Anthony Butch, Thomas Coates, Rita Effros, John Fahey, Beth Jamieson, Otoniel Martínez-Maza, Eric N. Miller, John Oishi, Paul Satz, Harry Vinters, Dorothy Wiley, Mallory Witt, Otto Yang, Stephen Young, Zuo Feng Zhang.

Pittsburgh: University of Pittsburgh, Graduate School of Public Health: Charles R. Rinaldo (Principal Investigator), Lawrence Kingsley (Co-Principal Investigator), James T. Becker, Robert W. Evans, John Mellors, Sharon Riddler, Anthony Silvestre.

Data Coordinating Center: The Johns Hopkins University Bloomberg School of Public Health: Lisa P. Jacobson (Principal Investigator), Alvaro Muñoz (Co-Principal Investigator), Keri Althoff, Christopher Cox, Gypsyamber D'Souza, Stephen J. Gange, Elizabeth Golub, Janet Schollenberger, Eric C. Seaberg, Sol Su.

FUNDING:

NIH: National Institute of Allergy and Infectious Diseases: Robin E. Huebner; National Cancer Institute: Geraldina Dominguez; National Heart, Lung and Blood Institute: Cheryl McDonald. UO1-AI-35042, 5-M01-RR-00052 (GCRC), UO1-AI-35043, UO1-AI-37984, UO1-AI-35039, UO1-AI-35040, UO1-AI-37613, and UO1-AI-35041. This work was also supported in part by NIH grants R37 AI47734 (J.I.M.); T32 AI07140 (J.T.H.), and the University of Washington Center for AIDS Research, an NIH funded program (P30 AI027757), which is supported by the following NIH Institutes and Centers (NIAID, NCI, NIMH, NIDA, NICHD, NHLBI, NIA).

Ethics:

This study was approved by the Institutional Review Boards at Northwestern University, Chicago USA; University of California at Los Angeles, Los Angele USA; University of Pittsburgh, Pittsburgh USA; and Johns Hopkins University, Baltimore USA.

The Multicenter Hemophilia Cohort Studies (MHCS)

DESCRIPTION:

The first Multicenter Hemophilia Cohort Study (MHCS-I) evaluated and prospectively followed patients with hemophilia or a related coagulation disorder. Initiated in 1982,

this study particularly sought to understand the cause and natural history of HIV infection and AIDS in this population, which was at high risk for development of AIDS.

MEMBER:

James J. Goedert.

FUNDING:

Intramural Research Program, National Cancer Institute, National Institutes of Health

Ethics:

Genetic studies of this cohorts has been granted by the NIH Office of Human Subjects Research.

The Pumwani Sex Workers Cohort

DESCRIPTION:

The Nairobi (Pumwani) commercial sex worker cohort was established in 1985. Despite repeated exposures to HIV-1, a number of women in this cohort have remained HIV-1 uninfected for long periods of time and have been epidemiologically defined as HIV resistant.

MEMBERS:

Francis A. Plummer, Terry Blake Ball, Keith Fowke, Joshua Kimani, Larry Gelmon, Ma Luo, Elizabeth Ngugi

FUNDING:

The Pumwani Sex Workers Cohort has been supported by various grants from Canadian Institute of Health Research, Canadian International Development Agency, National Institute of Health (R01 AI56980), USA and Bill and Melinda Gates Foundation since 1985. This work was supported by a grant from the Bill and Melinda Gates Foundation and the Canadian Institutes of Health Research (HOP-43135) through the Grand Challenges in Global Health Initiative.

Ethics:

This study was approved by the Institutional Review Board of the University of Manitoba, Winnipeg Canada and Kenyatta National Hospital, Nairobi, Kenya.

The San Francisco City Clinic Cohort (SFCCC)

DESCRIPTION:

The San Francisco City Clinic Cohort (SFCCC) consists of approximately 6700 homosexual men recruited between 1978 and 1980 from a clinic for sexually transmitted diseases.

MEMBERS:

Susan Buchbinder

FUNDING:

This was supported by cooperative agreement (No U62/CCU900523) from the Centers for Disease Control, Atlanta, Georgia

Ethics:

This study was approved by the Institutional Review Board of the State of California-Health and Human Services Agency Committee for the Protection of Human Subjects

The Swiss HIV Cohort Study (SHCS)

DESCRIPTION:

The Swiss HIV Cohort Study (SHCS) is an ongoing multi-center research project dealing with HIV infected adults aged 16 years or older. Since it was established in

1988, the SHCS has recruited and followed more than 17,000 patients in seven centers. The data are gathered by the Five Swiss University Hospitals, two Cantonal Hospitals, 15 affiliated hospitals and 36 private physicians (listed in http://www.shcs.ch/180-health-care-providers).

MEMBERS:

Aebi-Popp K, Anagnostopoulos A, Battegay M, Bernasconi E, Böni J, Braun DL, Bucher HC, Calmy A, Cavassini M, Ciuffi A, Dollenmaier G, Egger M, Elzi L, Fehr J, Fellay J, Furrer H, Fux CA, Günthard HF (President of the SHCS), Haerry D (deputy of "Positive Council"), Hasse B, Hirsch HH, Hoffmann M, Hösli I, Huber M, Kahlert CR (Chairman of the Mother & Child Substudy), Kaiser L, Keiser O, Klimkait T, Kouyos RD, Kovari H, Ledergerber B, Martinetti G, Martinez de Tejada B, Marzolini C, Metzner KJ, Müller N, Nicca D, Paioni P, Pantaleo G, Perreau M, Rauch A (Chairman of the Scientific Board), Rudin C, Scherrer AU (Head of Data Centre), Schmid P, Speck R, Stöckle M (Chairman of the Clinical and Laboratory Committee), Tarr P, Trkola A, Vernazza P, Wandeler G, Weber R, Yerly S.

FUNDING:

This study has been financed within the framework of the Swiss HIV Cohort Study, supported by the Swiss National Science Foundation (grant #201369), by SHCS

project #841 and by the SHCS research foundation. The data are gathered by the Five Swiss University Hospitals, two Cantonal Hospitals, 15 affiliated hospitals and 36 private physicians (listed in http://www.shcs.ch/180-health-care-providers).

Ethics:

This study was approved by the Institutional Review Board of the participating centres: Ethikkommission beider Basel; Kantonale Ethikkommission Bern; Comité départemental d'éthique des spécialités médicales et de médecine communautaire et de premier recours, Hôpitaux Universitaires de Genève; Commission cantonale d'éthique de la recherche sur l'être humain, Canton de Vaud; Comitato etico cantonale, Repubblica e Cantone Ticino; Ethikkommission des Kantons St. Gallen; Kantonale Ethikkommission Zürich

Urban Health Study: Genetics Cohort (UHSGC)

DESCRIPTION:

The Urban Health Study was a serial, cross-sectional sero-epidemiological study. Data were collected every 6 months in communities with a high prevalence of injection drug use: 35 semi-annual "waves" across 19 years of data collection in the San Francisco Bay Area CA U.S. (1986-2005). Eligibility criteria included having injected

drugs in the past 30 days, as confirmed by visual inspection. From the UHS we have banked biospecimens from about 15,000 people who injected drugs (PWIDs). From these UHS participants we developed the UHSGC, genotyping selected HIV+ cases and exposure-matched HIV- controls (total N = 3,136).

MEMBERS:

Laura J. Bierut, Nathan C. Gaddis, Cristie Glasheen, Dana B. Hancock, Eric O. Johnson (PI: UHSGC), Alex H. Kral (PI: UHS), Joshua L. Levy, Grier Page, Nancy L. Saccone

FUNDING:

U.S. National Institute on Drug Abuse (grants R01DA026141, R01DA038632).

Ethics:

This study was approved by the Research Triangle Institute (RTI) Institutional Review Board under the RTI International Office of Research Protection, North Carolina USA.

The US military HIV Natural History Study (NHS)

DESCRIPTION:

The US military HIV Natural History Study is a longitudinal epidemiological, observational, open cohort study collecting retrospective and prospective data in the U.S. Military active duty and Department of Defense (DoD) health care beneficiary HIV infected population.

MEMBERS:

Brian Agan, Mary Bavaro, Helen Chun, Nancy Crum-Cianflone, Cathy Decker, Connor Eggleston, Tomas Ferguson, Susan Fraser, Anuradha Ganesan, Joshua Hartzell, Joshua Hawley, Gunther Hsue, Arthur Johnson, Mark Kortepeter, Tahaniyat Lalani, Grace Macalino, Scott Merritt, Robert O'Connell, Jason Okulicz, Sheila Peel, Michael Polis, John Powers, Rose Ressner, Edmund Tramont, Tyler Warkentien, Amy Weintrob, Timothy Whitman, Michael Zapor

Ethics:

This study was approved by the Institutional Review Board of the Uniformed Services University of the Health Sciences, Bethesda USA

FUNDING:

Support for this work was provided by the Infectious Disease Clinical Research Program (IDCRP), a Department of Defense (DoD) program executed through the

Uniformed Services University of the Health Sciences. This project has been funded in whole, or in part, with federal funds from the National Institute of Allergy and Infectious Diseases, National Institutes of Health (NIH), under Inter-Agency Agreement Y1-AI-5072. The content of this publication is the sole responsibility of the authors and does not necessarily reflect the views or policies of the NIH or the Department of Health and Human Services, the DoD or the Departments of the Army, Navy or Air Force. Mention of trade names, commercial products, or organizations does not imply endorsement by the U.S. Government.

The Rural clinical cohort (RCC)

DESCRIPTION:

The Rural clinical cohort (RCC) is an open clinical cohort established in 1990 by the Medical Research Council (MRC), UK in collaboration with the Uganda Virus Research Institute (UVRI), to study the natural history of HIV infection and later the impact of ART after its introduction in 2004. Full details of the cohort structure and the annual HIV survey have been previously published.

MEMBERS:

Anatoli Kamali (formerly UVRI), Pontiano Kaleebu (UVRI), Manjinder Sandhu (WSI)

FUNDING:

Support for this work was provide by the UK Medical Research Council (grants G0901213-92157, G0801566, and MR/K013491/1), core funding of the collaboration between MRC and UVRI as well as Wellcome (WT098051),

Ethics:

This study was approved by the Uganda Virus Research Institute Science Ethics Committee and the IRB of the Uganda National Council for Science and Technology.

International AIDS Vaccine Initiative (IAVI)

DESCRIPTION:

The International AIDS Vaccine Initiative (IAVI) is the nonprofit organization dedicated to accelerating development of vaccines to prevent AIDS.

MEMBERS:

Matthew A Price (IAVI/UCSF)

FUNDING:

This work was funded in part by IAVI with the generous support of the United States Agency for International Development (USAID) and other donors. The full list of IAVI donors is available at http://www.iavi.org. The contents of this manuscript are the responsibility of IAVI and co-authors and do not necessarily reflect the views of USAID or the US Government.

Ethics:

This study was approved by the following Institutional Review Boards: the Kenya Medical Research Institute Ethical Review Committee, the Kenyatta National Hospital Ethical Review Committee of the University of Nairobi, the Rwanda National Ethics Committee, the Uganda Virus Research Institute Science and Ethics Committee, the Uganda National Council of Science and Technology, the University of Cape Town Health Science Research and Ethics Committee, the University of Zambia Research Ethics Committee, the Bio-Medical Research Ethics Committee at the University of KwaZulu Natal, and the Emory University Institutional Review Board.

The African Transcriptome Resource (ATR)

DESCRIPTION:

The African Transcriptome Resource (ATR) has been established to develop a transcriptomics resource across diverse populations in Africa and to facilitate a better understanding of the genetic diversity and functional consequences on gene expression across African populations. To date this resource includes data generated from 1000 Genomes Project populations.

MEMBERS:

Manjinder Sandhu (Wellcome Sanger Institute), Paul Flicek (European Bioinformatics Institute), Jacques Fellay (École Polytechnique Fédérale de Lausanne), Paul McLaren (University of Manitoba), Stephen Montgomery (Stanford University)

FUNDING:

This work was funded by Wellcome (WT206194), IAVI's USAID Cooperative Agreement (AID-OAA-A-16-00032), the European Molecular Biology Laboratory, the École Polytechnique Fédérale de Lausanne, the Public Health Agency of Canada and Stanford University.
